# Characterization of the Mel1c melatoninergic receptor in platypus (*Ornithorhynchus anatinus*)

**DOI:** 10.1371/journal.pone.0191904

**Published:** 2018-03-12

**Authors:** Célia Gautier, Sophie-Penelope Guenin, Isabelle Riest-Fery, Tahlia Jade Perry, Céline Legros, Olivier Nosjean, Valerie Simonneaux, Frank Grützner, Jean A. Boutin

**Affiliations:** 1 PEX Biotechnologie Chimie & Biologie, Institut de Recherches Servier, Croissy sur Seine, France; 2 Institut des Neurosciences Cellulaires et Intégratives, Strasbourg, France; 3 School of Biological Sciences, The University of Adelaide, Adelaide, Australia; 4 Institut de Recherches Internationales Servier, Suresnes, France; Senckenberg am Meer Deutsches Zentrum fur Marine Biodiversitatsforschung, GERMANY

## Abstract

Melatonin is a neurohormone produced in both animals and plants. It binds at least three G-protein-coupled receptors: MT_1_ and MT_2_, and Mel1cGPR. Mammalian GPR50 evolved from the reptilian/avian Mel1c and lost its capacity to bind melatonin in all the therian mammal species that have been tested. In order to determine if binding is lost in the oldest surviving mammalian lineage of monotremes we investigated whether the melatonin receptor has the ability to bind melatonin in the platypus (*Ornithorhynchus anatinus*), and evaluated its pharmacological profile. Sequence and phylogenetic analysis showed that platypus has in fact retained the ancestral Mel1c and has the capacity to bind melatonin similar to other mammalian melatonin receptors (MT_1_ and MT_2_), with an affinity in the 1 nM range. We also investigated the binding of a set of melatoninergic ligands used previously to characterize the molecular pharmacology of the melatonin receptors from sheep, rats, mice, and humans and found that the general profiles of these compounds make Mel1c resemble human MT_1_ more than MT_2_. This work shows that the loss of GPR50 binding evolved after the divergence of monotremes less than 190MYA in therian mammals.

## Introduction

Melatonin is a neurohormone produced by the pineal gland and maintains the circadian information through diffusion in the blood circulation to peripheral organs. Melatonin exists in almost all living organisms, and its actions are mediated by binding to two membrane receptors (MT_1_ and MT_2_) [1], a nuclear receptor [2] (although this observation has been challenged [3]), or a protein (quinone reductase 2; QR2) [4]. If the MT_1_ and MT_2_ receptors bind melatonin with an affinity of 1 nM and below, QR2 has an affinity for melatonin in the 10 μM range. In mammals, a third receptor exists: the melatonin-related receptor (MRR), recently renamed GPR50 [5,6], that does not bind melatonin but could regulate the activity, specificity, and signaling pathways of MT_1_ via dimerization [1]. GPR50 is thus an orphan receptor because its ligand, if any, is unknown. Mel1c has been described as a melatonin-binding receptor in amphibians, birds, and fish [5]. Dufourny *et al*. [7] demonstrated that the melatonin-related receptor or GPR50 in mammals is likely the protein that evolved from Mel1c on the basis of phylogenetic and sequence analyses. However, this discovery raised the question of why this receptor no longer binds melatonin in mammals.

Although the melatonin binding capacity of Mel1c and pharmacological profiles (e.g., in chicken) have been known for quite a long time [8–10], in depth comparative molecular pharmacological evidence is lacking. In fact, the few data available on the activity of the Mel1c receptor, particularly from *Xenopus laevis*, are functional ones, with some compounds using a cell-based assay, pigment aggregation in melanophores [9,11–14]. Further to this, some data is available on binding recorded using chicken brain membranes (see for example, Sugden [15] or Pegurier *et al*. [16]).

Genes involved in reproduction undergo accelerated evolution [17–19], and it seems that the evolution of genes, when fast, is often linked to reproduction. In this sense, we assumed that the passage from eggs to viviparity could be accompanied by many different changes, including from a melatonin-binding Mel1c (in amphibians and birds) to a sequence-related non-melatonin binding GPR50. In that sense, we wanted to better understand the evolution of Mel1c to GPR50 binding in mammals by investigating the oldest surviving monotreme lineage. Monotremes (platypus, echidna) are the oldest surviving mammals and provide unprecedented insight into mammalian evolution regarding, for example, the evolution of sex chromosomes [20,21].

Here we analyzed the evolution and function of the melatonin receptor Mel1c/GPR50 in the iconic egg-laying platypus. Interestingly, we found that the platypus Mel1c receptor shares high similarity with bird and other vertebrate Mel1c, while the therian ortholog GPR50 is more diverged. Furthermore, we found that platypus Mel1c binds melatonin while this binding has been lost in other mammals. Further, we analyzed if this change resulted in other pharmacological responses, which is not the case. Overall this work provides novel information about the functional evolution of this enigmatic receptor.

## Materials and methods

### Reagents and ligands

The radioligand 2-[^125^I]-iodomelatonin (specific activity: 2200 Ci/mmol) was purchased from Perkin Elmer (Courtaboeuf, France). Melatonin was obtained from Sigma (St Louis, MO) and 4-phenyl-2-propionamidotetraline (4P-PDOT) and 2-benzyl-N-acetyltryptamine (luzindole) from Tocris (Bristol, UK). Compounds were dissolved in dimethylsulfoxide (DMSO) at a stock concentration of 10 mM and stored at –20°C.

### RNA extraction and cDNA synthesis

Platypus specimens were killed with an intraperitoneal injection of pentobarbitone sodium (Nembutal) or pentobarbital (Lethabarb) at a dose of 0.1mg g-1. Tissue samples were snap-frozen. RNA was extracted from whole brain tissue using TRIzol (Invitrogen, USA) according to the manufacturer’s instructions. The RNA was resuspended in nuclease-free water and stored at -80°C. The cDNA was synthesized from 3 μg RNA using Superscript III Reverse Transcriptase (Invitrogen) following the manufacturer’s instructions. Briefly, RNA was treated with DNase I (Roche) to remove genomic DNA, and then incubated with 50 ng of random hexamers and 0.5 μl of 10 mM dNTPs for 5 min at 65°C. After incubation, 2 μl of 5× First-strand RT buffer, 0.5 μl of 0.1 M dithiothreitol (DTT), 0.5 μl of RNaseOUT™ (40 U/μl), and 0.5 μl of SuperScript III Reverse Transcriptase (200 U/μl) were added and the mixture incubated at 25°C for 10 min. This was followed by incubation at 50°C for 50 min and the final termination at 85°C for 5 min. Finally, 0.2 μl of RNase H (Biolabs, 5 U/μl) was added to each tube and incubated at 37°C for 20 min. The cDNA was stored at -20°C. All experimental approaches were performed according to the University of Adelaide biosafety and ethics committee regulations (Institutional Biosafety Committee, Dealing ID 12713) concerning this and the previous experiments of Dr. Grützner’s laboratory. Platypus samples were collected in 2002 (AEEC permit R.CG.07.03, Environment ACT permit LI 2002 270, NPWS permit A193) and 2008 (AEC permit N°. S-49-2006) at the Upper Barnard River (New South Wales, Australia).

### Cloning of platypus Mel1c receptor

PCR reactions were performed in a final volume of 50 μl using Q5 High-Fidelity DNA Polymerase (NEB, Ipswich, MA, USA) following the manufacturer’s protocol with 2 μl of platypus brain cDNA using the primers described in Table 1. The PCR conditions were as follows: 98°C for 3 min, followed by 10 cycles of 98°C for 10s, 65°C−1°C/cycle for 30s, 72°C for 30 s, and then 30 cycles of 98°C for 10s, X°C for 30s, 72°C for 30 s, and finally 2 min at 72°C.

**Table 1:** Primer sequences used in this study.

Amplicons	Forward primer sequences	Reverse primer sequences	Annealing temperature
	(5’–3’)	(3’–5’)	(°C)
Exon 1	ATGCCGGGAGCCGGGAAC GGGACCTGCCCGGGCT	CGTTGCGGAGCTTTCTGTT CCTCAGC	60
Exon 2	GCAACGCCGGGAACATCTT TGTGATCAG	TTACACATGGATTTCAGCTT GATTTTTCTTTG	58

Amplicons were separated on 1% agarose gels stained with ethidium bromide and the gel bands revealed with U Genius (Syngene, Frederick, USA). PCR products were purified using the High Pure Purification kit (Roche Mannhein Germany). Eluted DNA were inserted into the blunt pJET vector using the CloneJET PCR Cloning Kit (Thermo Fisher Scientific, Waltham, USA) and transformed into DH10β chemically competent *Escherichia coli* cells (NEB, Ipswich, MA, USA). Forward and reverse sequencing reactions were performed using the Big Dye Terminator Cycle Sequencing Ready Reaction Kit (Applied Biosystems, Life Technologies Corporation, Carlsbad, CA, USA) and pJET1.2 Sequencing Primers. Sequencing products were purified using the BigDye XTerminator® Purification Kit (Thermo Fisher Scientific, Waltham, USA) and run on an ABI 3730 XL automated sequencer (Applied Biosystems). Data were analyzed by Sequencher® version 5.4.6 DNA sequence analysis software (Gene Codes Corporation, Ann Arbor, MI USA).

### Phylogenetic analysis

All phylogenetic analyses were undertaken using Geneious V10.0.2 (http://www.geneious.com, [22]). Platypus (*O*. *anatinus*), chicken (*G*. *gallus*) and the clawed frog (*X*. *laevis*) nucleotide sequences were imported into Geneious and translated to protein sequences. Nucleotide sequences for a range of therian mammals (group consisting of eutherians and marsupials) were downloaded from NCBI (National Center for Biotechnology Information); names and GenBank accession numbers are shown in Table 2. Introns were removed and coding regions were translated according to their individual start and stop positions. DNA and protein alignments were undergone using ClustalW algorithm. Phylogenetic tree was built using Neighbour-Joining method, with Jukes-Cantor substitution model, 1000 bootstrap replicates and no outgroup was selected.

**Table 2:** Therian mammals used for phylogenetic analysis.

Scientific Name	Common Name	GenBank Accession Number
*Pan troglodytes*	Chimpanzee	736267
*Bos Taurus*	Cow	530065
*Sus scrofa*	Pig	100620104
*Canis lupus familiaris*	Dog	492213
*Dipodomys ordii*	Ord's kangaroo rat	105992838
*Monodelphis domestica*	Gray short-tailed opossum	100025945
*Phascolarctos cinereus*	Koala	110199994
*Sarcophilus harrisii*	Tasmanian devil	100913368

### Establishment of the transient COS7/3HA-Mel1c cell line

Coding sequences for the platypus (XM 001512887), *X*. *laevis* (U67880), and *G*. *gallus* (NM 205361.1) Mel1c melatonin receptors were synthesized by Genecust (Dudelange, Luxemburg) and subcloned into the pCIneo expression vector (Promega Madison, Wisconsin, USA). COS7 cells obtained from the American Type Culture Collection were maintained in DMEM GlutaMAX (Thermo Fisher Scientific, Waltham, USA) supplemented with 10% (v/v) fetal calf serum. Nucleofection of COS7 cells was performed according to the manufacturer's instructions using the Nucleofector machine (Amaxa, Cologne, Germany). Adherent COS7 cells were washed in phosphate-buffered saline (PBS), trypsinized, and gently resuspended in V nucleofection solution to a final concentration of 7 × 10^6^ cells/100 μl. Next, 5 μg of pCIneo/Mel1c vector was mixed with 100 μl of the COS7 cell suspension, transferred to a 2.0 ml electroporation cuvette, and nucleofected using program A024 and an Amaxa Nucleofector apparatus. Just after the nucleofection, 500 μl of media supplemented with 20% (v/v) fetal calf serum was added to the cell suspension and cultured in a humidified 37°C, 5% CO_2_ incubator for 20 minutes. Fourteen million cells were transferred to a T225 flask containing 50 ml of media supplemented with 20% (v/v) fetal calf serum and cultured in a humidified 37°C, 5% CO_2_ incubator. Two days after transfection, the cells were harvested in PBS, pelleted, and stored at -80°C until use.

### Indirect immunofluorescence

COS7 cells transiently expressing platypus (*O*. *anatinus*), *X*. *laevis*, or *G*. *gallus* Mel1c receptors were seeded at 50,000 cells per well in eight-well Lab-Tek chamber slides (Nunc, Naperville, IL) in 0.5 ml of medium. Cells were fixed by treatment with 4% formaldehyde in PBS for 15 min then incubated with glycine (100 mM) in PBS for 10 min at room temperature (RT). The cells were blocked with 0.2% bovine serum albumin (BSA) in PBS for 5 min at RT. For visualization of HA epitope-tagged Mel1c receptors, cell surface receptors were stained using a mouse monoclonal anti-HA antibody (0.25 μg/ml) for 30 min at 37°C in PBS containing 0.2% BSA. After washing in PBS supplemented with 0.2% BSA, cells were blocked with a solution of 0.2% BSA and 10% fetal calf serum. The cells were then incubated in a humidified chamber for 60 min at 37°C with secondary antibody (1 μg/ml Alexa fluor 488-conjugated goat anti-mouse antibody; St Louis, MO). The cells were washed twice with PBS/0.2% BSA and coverslips applied using Vectashield® Mounting medium containing 4′, 6′-diamidino-2-phenylindole to stain nuclei (Vector Lab, Burlingame, CA). Fluorescence microscopy was performed using a Zeiss Axiovert 200M microscope (Zeiss, Oberkochen, Germany). Immunofluorescent images were captured by an AxioCam MR3 camera and AxioVision software (Zeiss, Oberkochen, Germany).

### Establishment of stable CHO-FlpIn Mel1c cell lines

CHO-Flp-In cells (Thermo Fisher Scientific, Waltham, USA) were cultivated in the presence of zeocin (0.1 mg/ml). The cells were cotransfected with either platypus (cloned sequence that will be submitted to GenBank) Mel1c/pcDNA5-FRT (platypus Mel1c) or *X*. *laevis* (NM 001087919.1) Mel1c/pcDNA5-FRT (clawed frog Mel1c) and Flp recombinase expression pOG44 plasmid using PEIpro (Invitrogen, Carlsbad, CA). CHO cells stably expressing platypus Mel1c or clawed frog Mel1c were selected using hygromycin (0.6 mg/ml). CHO-Flp-In-platypus Mel1c cells were grown in HAM F12 with 2 mM glutamine and supplemented with 10% fetal calf serum. Cells were selected using antibiotic pressure (hygromycin) for 72 h. The resistant cells were pooled and the presence of the transgene confirmed by RT-PCR (not shown) using 500,000 cells. After confirmation of the presence of the transgene, the cells were amplified up to 2 x 10^9^ cells. This material was used to prepare the cell membranes used in further pharmacological experiments.

### Membrane preparation

This standard procedure was described in a previous experiment with CHO cells expressing the sheep MT_2_ receptor [23]. Briefly, CHO cells expressing either the platypus or clawed frog Mel1c melatonin receptor were grown to confluence, harvested in phosphate buffer containing 2 mM EDTA, and centrifuged at 1000*g* for 5 min at 4°C. The resulting pellet was suspended in 5 mM Tris/HCl (pH 7.4) containing 2 mM EDTA and homogenized using a Kinematica polytron. The homogenate was then centrifuged (20,000*g*, 30 min, 4°C) and the resulting pellet suspended in 75 mM Tris/HCl (pH 7.4) containing 2 mM EDTA and 12.5 mM MgCl_2_. The protein content was determined according to the method of Lowry *et al*. [24] using the Bio-Rad kit (Bio-Rad SA, Ivry-sur-Seine, France). Aliquots of membrane preparations were stored in binding buffer (50 mM Tris/HCl, pH 7.4 containing 5 mM MgCl_2_ and 1 mM EDTA) at –80°C until use.

### 2-[^125^I]-melatonin binding assay

This approach has been described very often by our laboratory and others. Briefly, membranes were incubated for 2 hours at 37°C in a final volume of 250 μl of binding buffer containing 50 pM 2-[^125^I]-melatonin for competition experiments. The results were expressed as Ki, taking into account the concentration of radioligand used in each experiment. Non-specific binding was defined with 10 μM melatonin. The reaction was stopped by rapid filtration through GF/B Unifilters, followed by three successive washes with ice-cold buffer. Data were analyzed by using the program PRISM (GraphPad Software Inc., San Diego, CA). For saturation assays, the density of binding sites (B_max_) and the dissociation constant of the radioligand (K_D_) were calculated according to the Scatchard method. For competition experiments, inhibition constants (K_i_) were calculated according to the Cheng-Prusoff equation: K_i_ = IC_50_/[1+(L/K_D_)], where IC_50_ is the measured 50% inhibitory concentration, L is the concentration of 2-[^125^I]-iodomelatonin, and K_D_ is the dissociation constant. A similar protocol was used when the binding was performed on whole cells, as described by Legros *et al*. [25]. As per Plos One policy, all the raw data–listings from radioactivity counter are attached as raw files (Supplementary Tables A to M in S1 Data).

## Results

### Cloning of platypus Mel1c receptor

**Fig 1:**
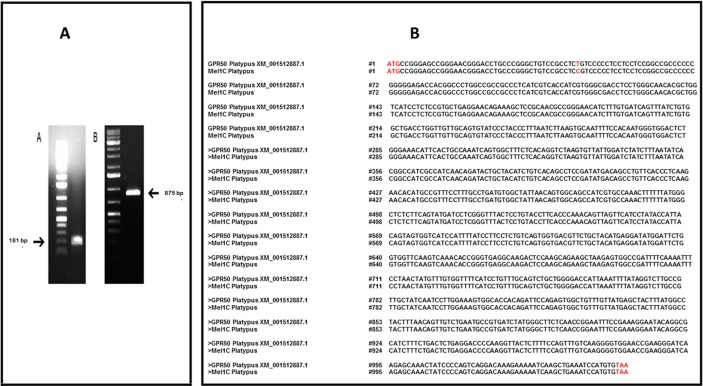
Cloning of Mel1c from platypus brain and comparison of the actual and predicted sequences. The cloning was performed using the predicted sequence XM_001512887. A, lane 1: Exon 1 amplicon, 181 bp; lane 2: Exon 2 amplicon, 875 bp. B, Comparison of the obtained sequence. In red: ATG starting codon and TAA stop codon. At position 44, a single mutation from the predicted sequence changes one amino acid in the protein sequence at position 15 (L>P).

The two amplicons isolated from exons 1 and 2 are depicted in Fig 1A. They were fully sequenced. Fig 1B shows the complete nucleotide sequence of the gene. The alignment between the predicted and experimental sequences is shown. The platypus Mel1c sequence fits the predicted sequence XM 001512887 (www.ncbi.nlm.nih.gov/nuccore/XM/001512887) except for a single mutation in position 44 (T>C). This mutation changes the nature of the amino acid encoded by this triplet at position 15 (L>P).

### Gene organization and phylogenetic analysis

Platypus Mel1c is organized in a similar way as any melatonin receptor described in other vertebrates. Platypus Mel1c contains two exons [separated by a long intron (~30,000 bp) (Panel A in S1 Fig)] that encode the transmembrane domain 1 and the other six transmembrane domains. Protein alignment with chicken and the clawed frog shows that Mel1c in platypus is well conserved at the amino acid level. From Thr44 to Ala352, amino acid sequence identity was comprised between 72 and 78% for the 3 receptors. (see Table 3 and Panel B in S1 Fig). Interestingly, the Mel1c family has much more variation at the nucleotide level throughout these species (Panel C in S1 Fig).

**Table 3:** Comparison of Mel1c sequences in platypus, xenopus and chicken to the human GPR50.

	*Gallus gallus* Mel1c	*Homo sapiens* GPR50	*Ornithorynchus anatinus* Mel1c	*Xenopus laevis* Mel1c
*Gallus gallus* Mel1c		46.10%	78.40%	78.70%
*Homo sapiens* GPR50	46.10%		48.60%	39.10%
*Ornithorynchus anatinus* Mel1c	78.40%	48.60%		72.30%
*Xenopus laevis* Mel1c	78.70%	39.10%	72.30%	

**Fig 2:**
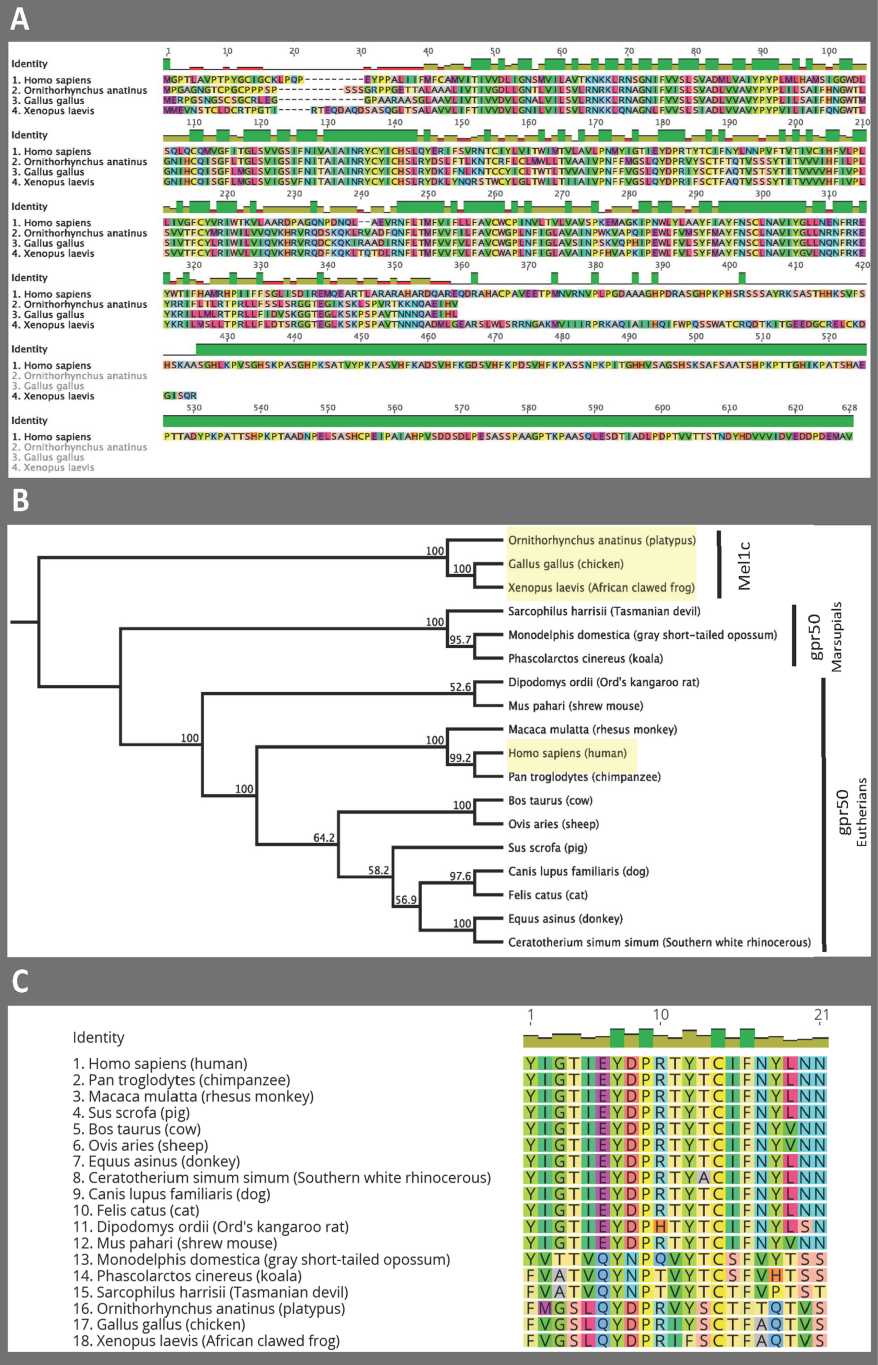
Platypus has retained ancestral Mel1c showing that the ortholog GPR50 evolved after the divergence of monotremes from therian mammals. A) Protein alignment of human (*Homo sapiens*) GPR50 with platypus (*Ornithorhynchus anatinus*), chicken (*Gallus gallus*) and the clawed frog (*Xenopus laevis*) Mel1c. Each amino acid is depicted by its single letter symbol and an associated colour. Identity bar above alignment denotes the similarity at each amino acid position between the four species: green = all homologous; yellow = three species homologous; red = two species homologous; no bar = no homology. Every 50 amino acid positions are labeled. B) Neighbour-Joining phylogenetic tree showing platypus clustering together with chicken and the clawed frog Mel1c while marsupial GPR50 and eutherian GPR50 form their own distinct clusters. Bootstrap support values are shown for each node. The four species from protein alignment in panel A are highlighted in yellow. C) Amino acid sequence of the second extracellular loop (E2) of Mel1c and GPR50 for all species used in the phylogenetic tree from panel B. Each amino acid is depicted by its single letter symbol and an associated colour. Identity bar above alignment denotes the similarity at each amino acid position between all species: green = all homologous; yellow = some species homologous. Every 10 amino acid positions are labeled.

When Mel1c is aligned with the human sequence of GPR50 (the therian ortholog of Mel1c), the divergence between the two is apparent (Fig 2A). Phylogenetic analysis shows platypus clustering with chicken and the clawed frog Mel1c to the exclusion of an array of therian mammal GPR50 (Fig 2B), showing here that platypus has retained the ancestral Mel1c gene and its ortholog GPR50 evolved after the divergence of monotremes from therian mammals. Although GPR50 has lost its capacity to bind melatonin, the sequence has been conserved in mammals (Table 3), with a major difference being that the sequence of human GPR50 has 270 additional amino acids compared to the platypus (Fig 2A), rendering the C-terminus of GPR50 one of the longest known G-protein-coupled receptors (GPCRs).

A recent study has highlighted the importance of the second extracellular domain (E2) of melatonin receptors in their ability to bind to melatonin [26]. Therefore, we also looked at this region between Mel1c and GPR50 in all species used for phylogenetic analyses. Here we found that platypus, chicken and the clawed frog contained a Gln (Q) at position 18 of E2 (Fig 2C), which was shown by Clement *et al*. [26] to be important for the binding function of melatonin receptors. However, all eutherian species contained a Thr (Y) at this position instead, while the marsupials had Thr (Y; opossum), His (H; koala) or Pro (P; Tasmanian devil) (Fig 2C). If this Gln in E2 is in fact important for binding in melatonin receptors, then the lack of it in all therian mammals tested here supports that hypothesis and also suggests the transition from Mel1c to GPR50 occurred between the divergence of monotremes and therian mammals– 190 to 160 million years ago (MYA).

### Expression and subcellular localization of recombinant platypus, clawed frog and chicken Mel1c receptors in COS7 cells

**Fig 3:**
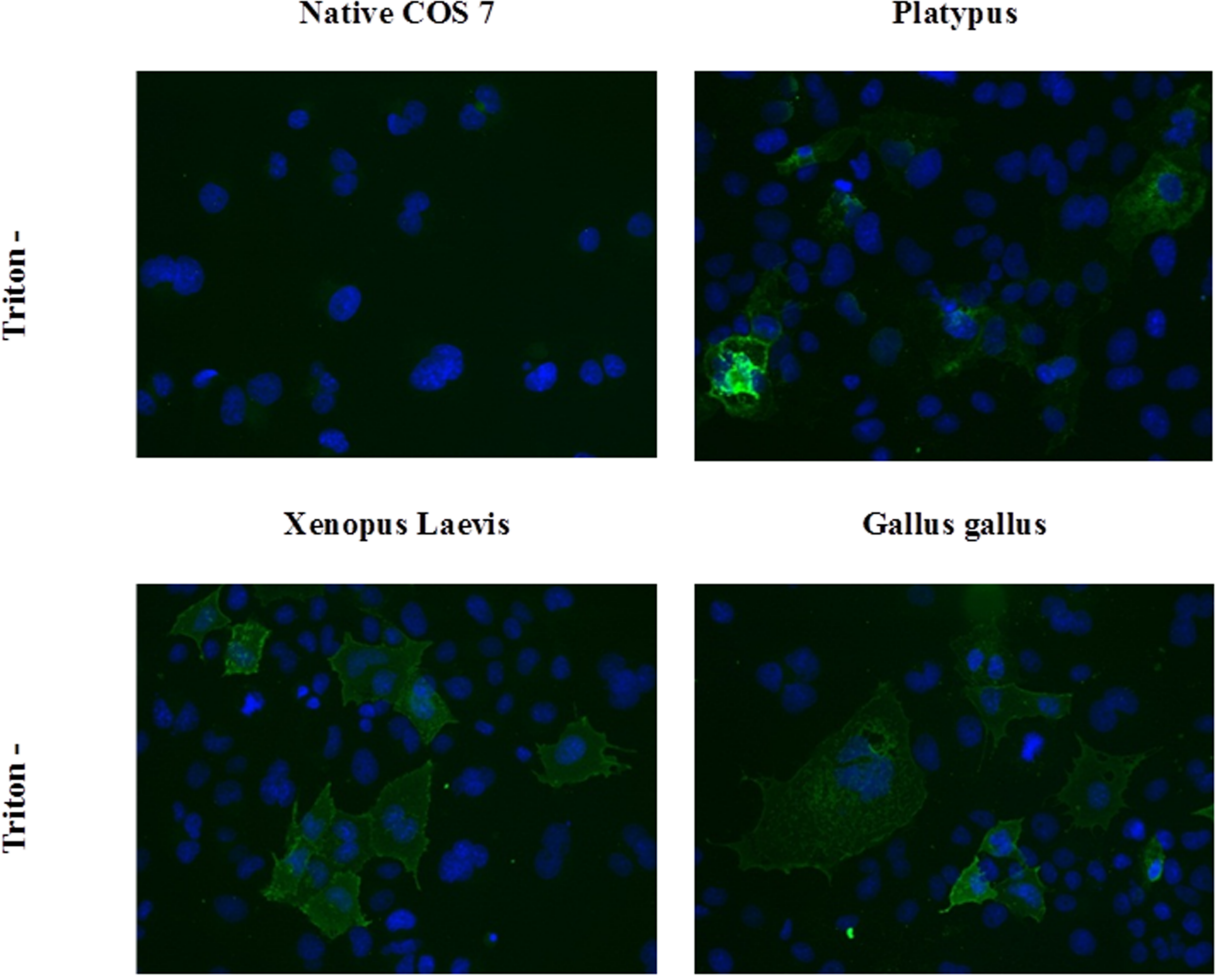
Expression of Mel1c receptors in COS7 cells. Subcellular localization of HA epitope-tagged Mel1c receptors. Immunofluorescence studies were performed with transfected COS7cells. COS7 cells and COS7 expressing 3HA-platypus Mel1C or 3HA-X. laevis Mel1C or 3HA-G. gallus Mel1C receptors were probed with mouse monoclonal anti-HA antibody directed against the N-terminal epitope tag present on recombinant receptor. Experiments were carried out with paraformaldehyde-fixed and non-permeabilized cells. The fluorescence images were obtained by using Alexa 488-conjugated goat anti- mouse IgG secondary antibody. COS7 cell nuclei were stained with 4′, 6′-diamidino-2-phenylindole. Native COS7 cells were used as a negative control.

Mel1c from *G*. *gallus* and *X*. *laevis* was synthesized according to the reported predicted sequences and built in the same expression vector as platypus Mel1c. The first step was to determine whether they are expressed and active in a cellular model. We started by fusing the N-terminus of the three sequences to a 3HA flag, for which potent antibodies exist. For this particular purpose, the transition expression of the receptors into COS7 cells led to the images in Fig 3. Immune staining of non-permeabilized COS7/3HA-Mel1c cells with a fluorescent anti-HA antibody revealed major expression of platypus, *X*. *laevis*, and *G*. *gallus* Mel1c receptors at the plasma membrane. No fluorescent signal was detected from native COS7 cells.

### Melatonin binding

**Fig 4:**
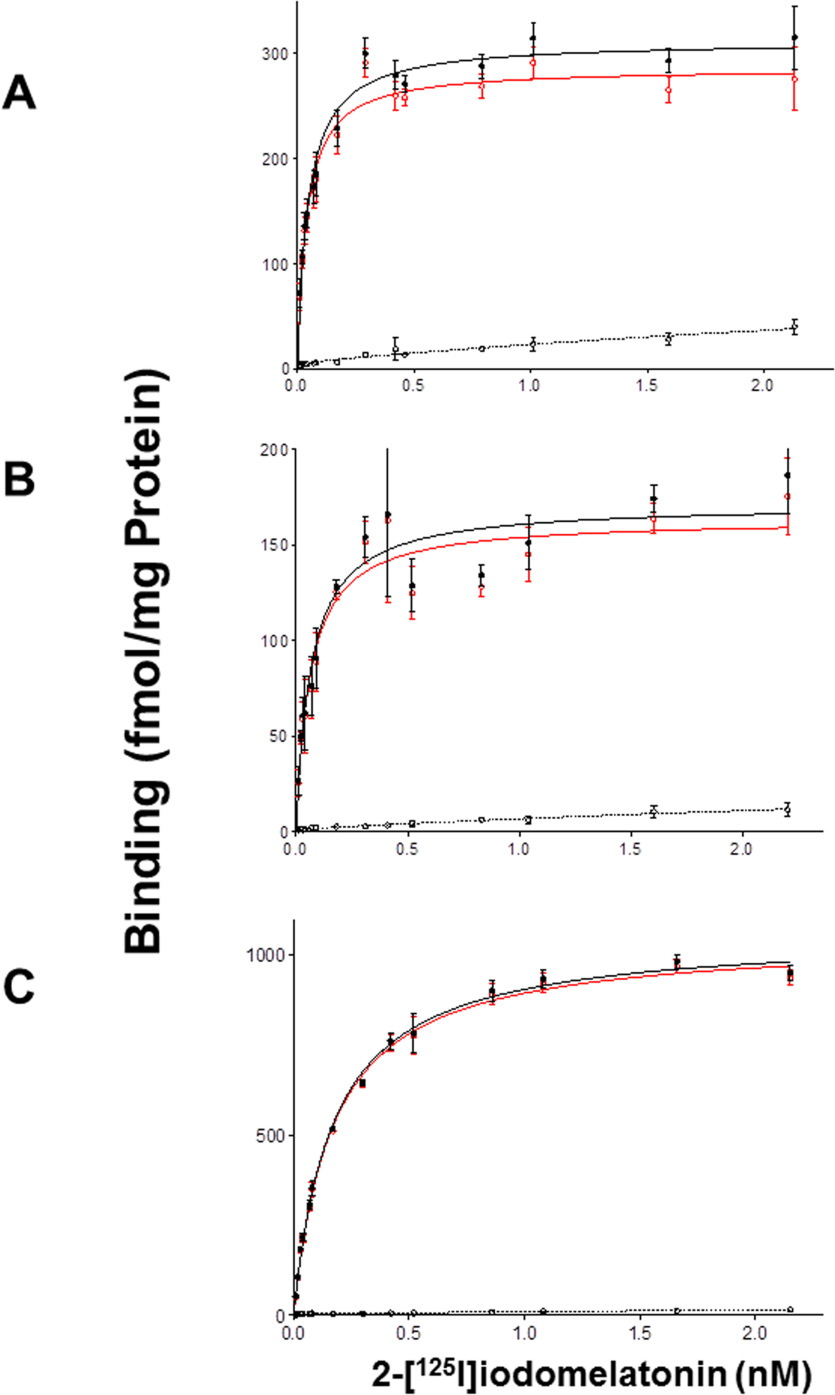
Saturation binding experiments for 2-[125I]-iodomelatonin. Membranes from COS7 cells transfected with platypus Mel1c (A), clawed frog Mel1c (B), and chicken Mel1c (C) were used to measure the binding at Mel1c receptors. Red line represents specific binding, black line represents total binding and dotted line represents non-specific binding.

Next, we prepared membranes for clawed frog, platypus, and chicken from cells overexpressing their respective Mel1c receptors and attempted to measure the binding of 2[^125^I]-iodomelatonin. Saturations were obtained and showed specific bindings for all species tested. This was the first time that the Mel1c receptor from a mammal was shown to bind 2[^125^I]-iodomelatonin. The saturation curves (Fig 4) for the three species were similar to those obtained under similar conditions for the melatonin receptors that have been cloned, expressed, and characterized in our laboratory. The expression levels of the Mel1c receptors from these species stably expressed in CHO cells were 18 fmol/mg of protein for clawed frog and 36 fmol/mg of protein for the platypus. These levels are in the lower range of expression for melatonin receptors (MT_1_ and MT_2_ receptors) from more classical species we generated previously, with expression levels ranging from 80 to 2650 fmol/mg of protein in rats and humans [27] and 300 to 700 fmol/mg of protein for mice [28]. In contrast, the K_D_ of the two Mel1c receptors for melatonin was 133 pM for clawed frog and 67 pM for platypus.

### Molecular pharmacology

To obtain stable expression of the receptor for future studies, a preliminary step involving transient expression of the receptor was performed. The Mel1c receptors from the three species were expressed in transiently transfected COS7 cells. A limited number of compounds were tested in order to gain a preliminary perception of the characteristics of Mel1c from these species. The profiles for chicken, clawed frog, and platypus are reported here for the first time. The comparative data are given in Table 4. The profiles comprise the binding affinities of a dozen compounds for the receptors of the three species. These profiles were quite similar, which was not surprising for the chicken and clawed frog, despite their evolutionary distance. However, the similarity with pharmacological profiles of the monotreme Mel1c was somewhat unexpected. Simple linear correlations between the sets of data (taken 2 by 2) led to R^2^ > 0.95. These correlations translate a view of the proximity of the receptor affinities to some compounds from a pure biochemical point of view.

**Table 4:** Comparison of the molecular pharmacology of platypus, xenopus and chicken melatonin Mel1c receptors.

	pKi *Ornithorhyncus anatinus*	pKi *Xenopus laevis*	pKi *Gallus gallus*
Melatonin	8.6	8.8	8.6
2-Iodomelatonin	10.2	10.0	9.8
S 70254	6.6	6.8	6.6
4P-P-DOT	6.7	6.7	6.4
Agolmelatin/S 20098	9.6	9.2	8.9
S 22153	7.6	7.0	6.7
Ramelteon/ FLN68	10.1	10.1	9.8
Luzindole	5.9	5.7	5.4

Binding experiments were performed with 2-[^125^I]-iodomelatonin as the radioligand using COS7 cells transiently transfected with the respective Mel1c gene. Experiments using two different transfections were performed separately. Each point was the mean of a triplicate determination. Concentration isotherm curves were obtained using 10 concentrations of each product from 10^−13^ to 10^−4^ M. S70254, 2-iodo-N-2-[5-methoxy-2-(naphthalen-1-yl)-1H-pyrrolo[3,2-b]pyridine-3-yl]) acetamide; 4P-P-DOT, N-[(2S,4S)-4-phenyl-1,2,3,4-tetrahydronaphthalen-2-yl]propanamide; Agomelatin^®^ (S20098), N-(2-(7-methoxynaphthalen-1-yl)ethyl)acetamide; S22153, N-[2-(5-ethylbenzothiophen-3-yl)ethyl]acetamide; Ramelteon^®^ (FLN68), (S)-N-(2-(1,6,7,8-tetrahydro-2H-indeno-(5,4)furan-8-yl)ethyl)propionamide; Luzindole, N-acetyl-2-benzyl-tryptamine.

**Fig 5:**
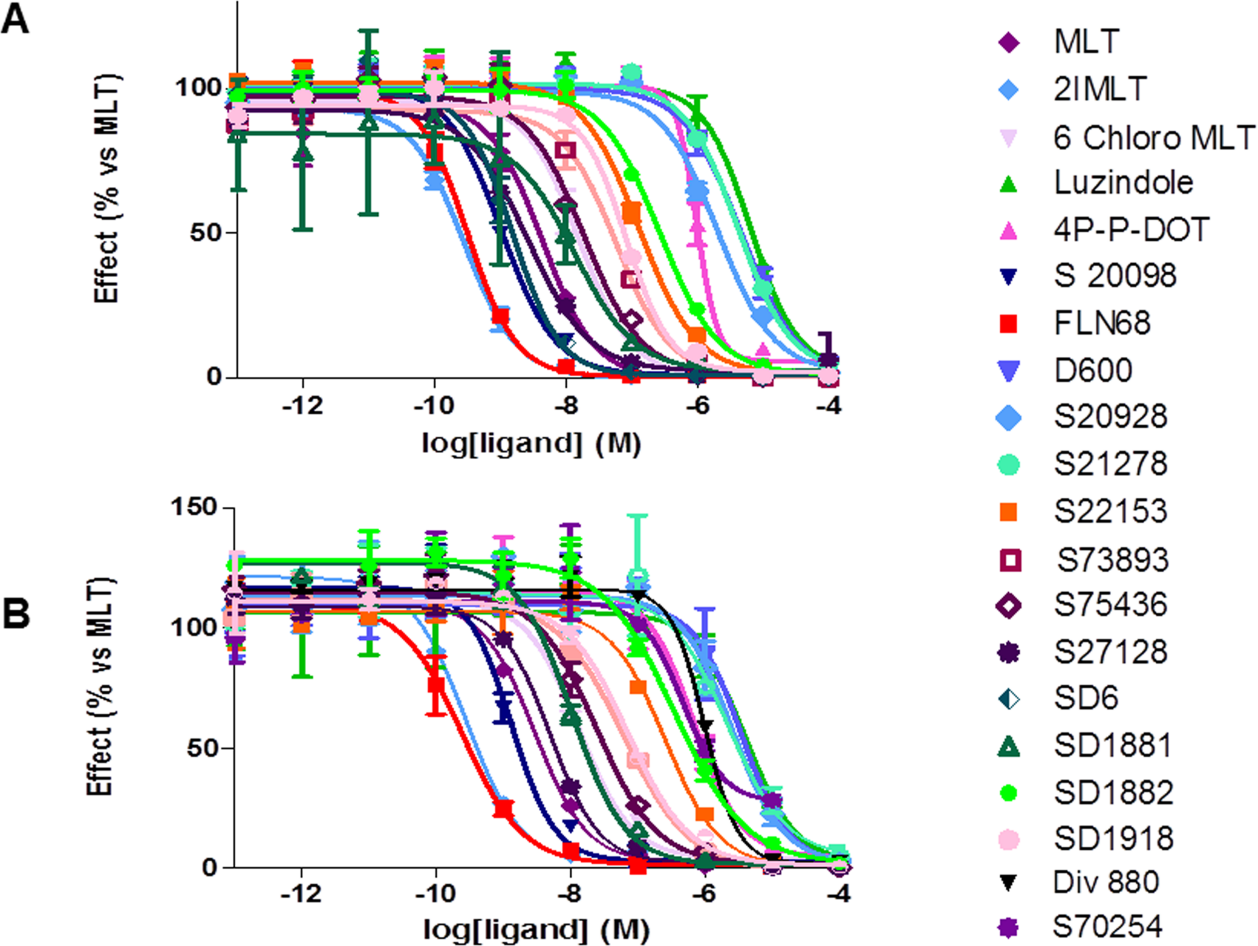
**Molecular pharmacology of the Mel1c receptors from platypus (A) and clawed frog (B).** The ligand was 2-[^125^I]iodomelatonin. Independent experiments were performed at least twice using different batches of membranes from stably transfected CHO cells and each point was obtained in triplicate. SD6, N-[2-(5-methoxy-1H-indol-3-yl)ethyl]iodoacetamide; 2IMLT, 2-iodomelatonin; 6-Cl- Chloro MLT, 6-chloromelatonin; Luzindole, N-acetyl-2-Br-MLT; 2-bromomelatonin, benzyltryptamine; 4P-P-DOT, N-[(2S,4S)-4-phenyl-1,2,3,4-tetrahydronaphthalen-2-yl]propanamide; Agomelatin^®^ (S20098), N-(2-(7-methoxynaphthalen-1-yl)ethyl)acetamide; Ramelteon^®^ (FLN68), (S)-N-(2-(1,6,7,8-tetrahydro-2H-indeno-(5,4)furan-8-yl)ethyl)propionamide; D600, methoxyverapamil; 5HT, 5-hydroxytryptamine; S20928, (N-[2-(1-naphthyl)ethyl] cyclobutanecarboxamide); S21278, N-[2-(6-methoxybenzimidazol-1-yl)ethyl]acetamide; S22153, N-[2-(5-ethylbenzothiophen-3-yl)ethyl]acetamide; S27128-1, N-[2-(2-iodo-5-methoxy-6-nitro-1H-indol-3-yl)ethyl]acetamide; S73893, N-[3-methoxy-2-(7-methoxy-1-naphthyl)propyl]acetamide; S75436, 2-fluoro-N-[3-hydroxy-2-(7-methoxy-1-naphthyl) propyl]acetamide; S77834S27128, N-[(8-[2-(2-iodo-5-methoxy-10,11-dihydro-5H-dibenzo[a,d][7] annulen-106-nitro-1H-indol-3-yl)methylethyl]acetamide; S77840, 1-[(8-methoxy-10,11-dihydro-5H-dibenzo[a,d][7]annulen-101H-indol-3-yl)methyl]urea ethyl]iodoacetamide; SD1881, N-[2-(6-iodo-5-methoxy-1H-indol-3-yl)ethyl]acetamideiodomelatonin; SD1882, N-[2-(4-iodo-5-methoxy-1H-indol-3-yl)ethyl]acetamide; SD1918, N-[7-iodomelatonin; Div 880, 2-(7-iodo-5-methoxy-1H-indol-3-yl)ethyl]acetamide2-[(2-iodo-4,5-dimethoxyphenyl)methyl]-4,5-dimethoxy phenyl; S70254, 2-iodo-N-2-[5-methoxy-2-(naphthalen-1-yl)-1H-pyrrolo[3,2-b]pyridine-3-yl])acetamide. Concentration isotherms were obtained using 10 concentrations of each product from 10^−13^ to 10^−4^ M.

Because the profiles of the Mel1c bindings are similar between birds (chicken) and amphibians (clawed frog), it seemed important to proceed to studies using only the Mel1c from clawed frog. Thus, we focused on the stable expression in CHO cells of the platypus and clawed frog Mel1c receptors. These two receptors were expressed at workable levels, although other melatonin receptors were sometimes expressed at up to 2 pmol/mg of protein [27] depending on the method chosen for establishing the stable cell line. The last step of this characterization was to obtain on this biological material, a pharmacological profile for a series of 19 ligands for these two species compared to the human melatonin receptors MT_1_ and MT_2_. The curves obtained for platypus Mel1c with the products are given in Fig 5A and for the clawed frog Mel1c in Fig 5B as examples.

Nineteen compounds were tested, from the classic melatonin ligands to the more obscure D600, a specific MT_1_ ligand [25], and the marketed drugs Ramelteon^®^ and Agomelatin^®^, as well as the iodinated derivatives of melatonin (e.g., 4-iodo, 2-iodo, or 6-iodo melatonin). The data were compared to that of the human MT_1_ and MT_2_ (Table 5).

**Table 5:** Molecular pharmacology of Mel1c receptors in platypus and xenopus compared to the human MT_1_ and MT_2_ melatonin receptors.

Compounds	Platypus Mel1C	Xenopus Mel1C	hMT_1_	hMT_2_
Melatonin	8.7	8.7	9.6	9.3
2-iodomelatonin	9.9	9.7	10.7	9.8
6-chloromelatonin	8.1	8.1	8.7	9.6
Luzindole	5.6	5.6	6.6	7.6
4P-PDOT	6.2	6.1	6.8	8.9
Agomelatin ®	9.4	9.1	9.9	9.9
Ramelteon ®	9.9	9.9	10.1	10.3
D600	5.7	5.7	7.0	<5
S20928	6.1	5.9	7.1	7.1
S21278	5.7	5.8	6.2	6.2
S22153	7.3	6.9	8.2	8.0
S73893	7.7	7.4	8.4	8.1
S75436	8.2	7.8	7.9	8.9
S27128	9.0	8.6	8.9	9.2
DIV880	6.2	5.9	6.1	8.0
SD6	9.4	9.3	9.9	9.9
6-Iodomelatonin	8.4	8.1	8.8	8.6
4-Iodomelatonin	7.0	6.6	7.8	7.9
7-Iodomelatonin	7.5	7.3	7.3	7.3
S70254	ND	6.3	7.0	9.0

ND: not determined. Luzindole, N-acetyl-2-benzyltryptamine; 4P-PDOT, N-[(2S,4S)-4-phenyl-1,2,3,4-tetrahydronaphthalen-2-yl]propanamide; D600, methoxyverapamil; S20928, (N-[2-(1-naphthyl)ethyl] cyclobutanecarboxamide); S21278, N-[2-(6-methoxybenzimidazol-1-yl)ethyl]acetamide; S22153, N-[2-(5-ethylbenzothiophen-3-yl)ethyl]acetamide; S73893, N-[3-methoxy-2-(7-methoxy-1-naphthyl)propyl]acetamide; S75436, 2-fluoro-N-[3-hydroxy-2-(7-methoxy-1-naphthyl)propyl]acetamide; S27128-1, N-[(8-methoxy-6-nitro-1H-indol-3-yl)ethyl]acetamide; DIV880, 2-(2-[(2-iodo-4,5-dimethoxyphenyl)methyl]-4,5-dimethoxy phenyl; SD6, N-[2-(5-methoxy-1H-indol-3-yl)ethyl]iodoacetamide; S70254, 2-iodo-N-2-[5-methoxy-2-(naphthalen-1-yl)-1H-pyrrolo[3,2-b]pyridine-3-yl])acetamide. Binding experiments were performed with 2-[125I]-iodomelatonin as the radioligand, Experiments were run in triplicate, at least twice using different batches of membranes from stably transfected CHO cells. Concentration isotherms were obtained using 10 concentrations of each product from 10^−13^ to 10^−4^ M.

**Fig 6:**
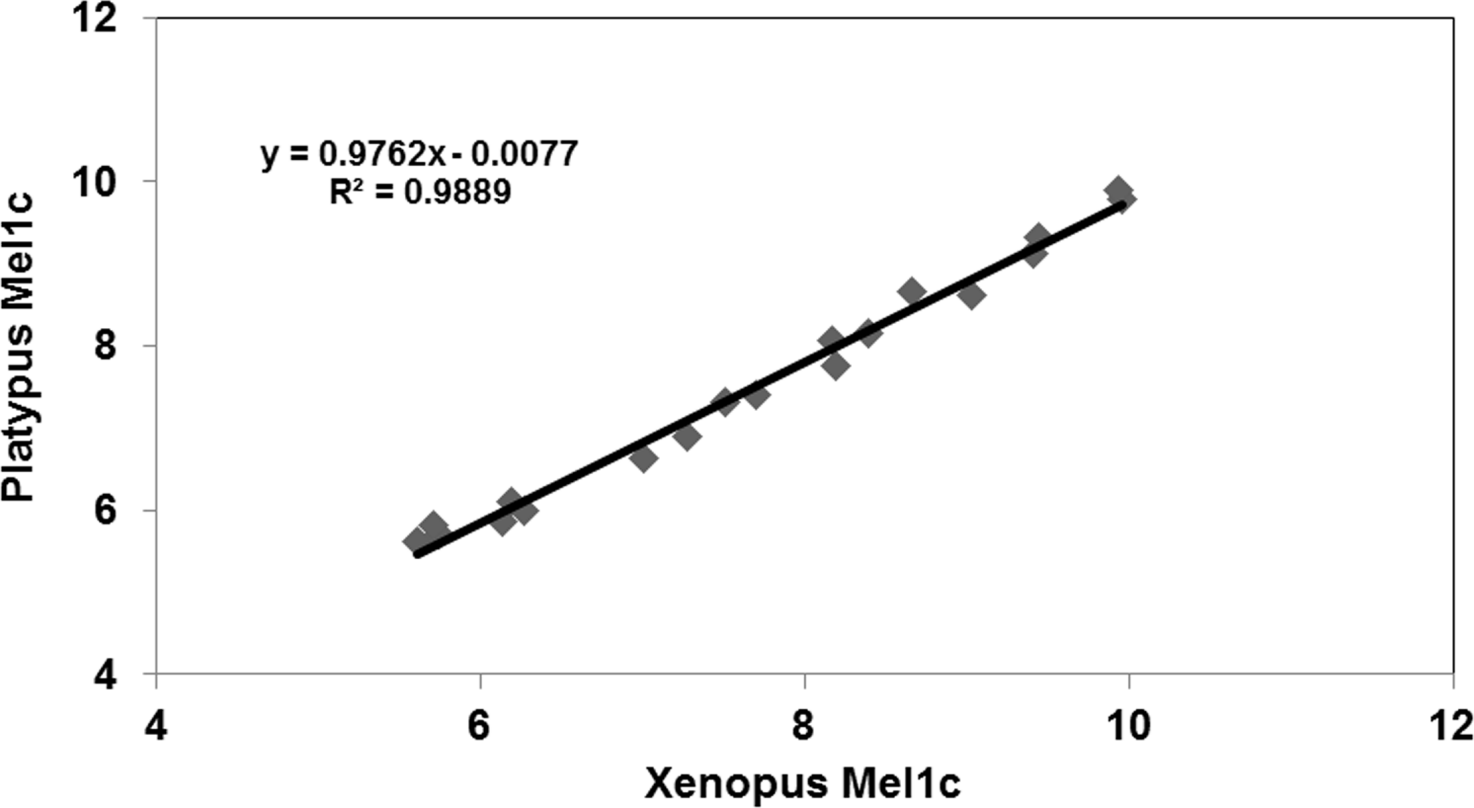
Correlation between the data obtained from platypus Mel1c and those obtained with clawed frog Mel1c. Data were obtained in a binding assay with 2-[^125^I]-iodomelatonin as the radioligand. See Fig 5.

We obtained an almost perfect match between the results obtained with chicken Mel1c, which is a reference in this domain, and those obtained with the platypus Mel1c that was cloned and characterized here for the first time. The simplest correlation coefficient (R^2^) was 0.98 (Fig 6).

**Fig 7:**
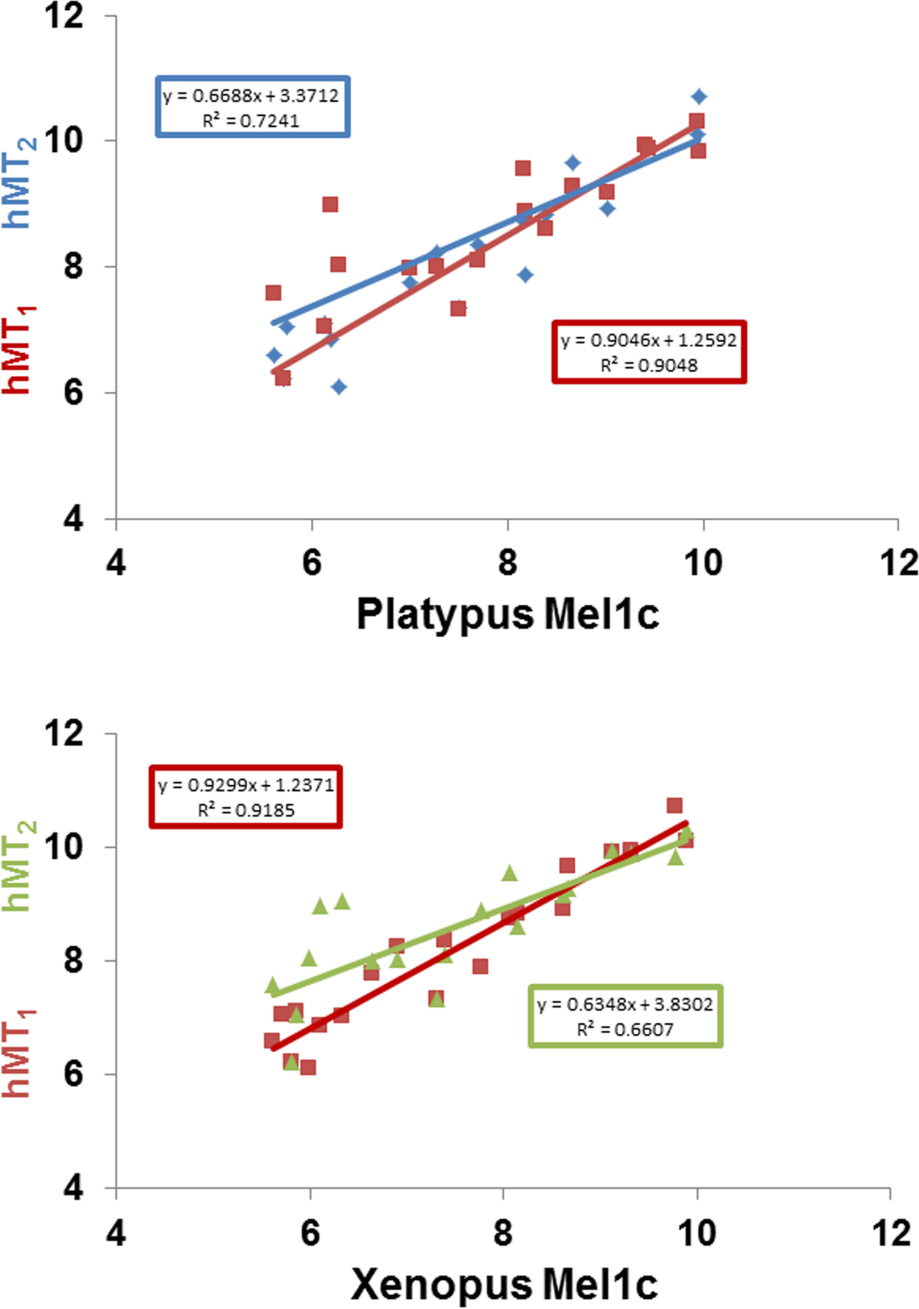
**Comparison of the correlations between the molecular pharmacology of platypus Mel1c (A) or clawed frog Mel1c (B) and human melatonin receptors MT**_**1**_ **and MT**_**2**_. The correlation in red is between hMT_1_ and platypus Mel1c; in blue, between hMT_2_ and Platypus Mel1c (upper panel). The correlation in red is between hMT_1_ and clawed frog Mel1c; and in green between hMT_2_ and clawed frog Mel1c (lower panel.) See Fig 5 for data.

At first glance, the data do not seem to differ from one species to another, with either the Mel1c receptor, as shown above with transiently expressed receptor (Table 4), or the human MT_1_ and MT_2_ receptors. In order to validate this observation, the data obtained with the clawed frog Mel1c receptor and platypus Mel1c receptor were plotted against the data obtained for MT_1_ and MT_2_ (Fig 7).

At first glance, Mel1c seems to behave identically towards this selection of compounds among the two species (human versus platypus), but it is quite different when the correlation coefficients are examined. Surprisingly, the Mel1c data correlate highly (R^2^ = 0.9) with MT_1_ and more poorly with MT_2_ (R^2^ = 0.7 for platypus and 0.6 for Clawed frog).

## Discussion

Platypus and echidna are the oldest surviving mammalian lineage, having diverged from other mammals approximately 190 MYA [29]. These species have an astonishing track record of informing us about the evolution of the mammalian genome [30] and individual genes (see for example Grutzner *et al*. [17], Cortez *et al*. [20], Tsend-Ayush *et al*. [31] and Hu *et al*. [32]). Comparing the GPCRs from different species can help us understand the structure of these receptors, as well as their binding characteristics [33]. To prepare for the expression, purification, crystallization, and characterization of receptors, a preliminary approach is to closely study the various receptors from diverse species. Natural sequences have evolved throughout these species; though maintaining specificity towards their natural ligand (see Li *et al*. for complete melatonin receptor evolutionary divergences [34]). They show that it is slightly different for the melatonin-related receptor (GPR50) and Mel1c.

The seminal work of Dufourny *et al*. [7] demonstrated from minute genetic analyses that the Mel1c receptor from amphibians and birds gave rise to GPR50 in mammals, which is still categorized as an orphan receptor because it does not bind melatonin or analogs, confirmed independently by us and others (C Ouvry & JA Boutin, unpublished). It has been a challenge for a long time to understand when GPR50 lost its binding during mammalian evolution and what this means for the function and evolution of melatonin and GPR50.

Studies of molecular evolution have been reported frequently in the literature. The trees of phylogenetic affiliation has been used to better understand the passage from one species to another, especially by studying species that seem far away from each other, as well as those that are closely related. Monotremes occupy a key position in mammalian phylogeny and the present paper attempted to determine the evolution and binding of melatonin. Therefore, whether the melatonin receptor cloned from platypus is in fact Mel1c or GPR50 was of interest.

The predicted sequence was very close to the actual sequence with a single mutation at position 44. The structure of the gene was as expected because of the common nature of all of the melatonin receptors throughout living species, two exons separated by a long intron of ~30,000 bp (Panel A in S1 Fig). Alignment of the platypus receptor with chicken and frog Mel1c shows very high similarity at the amino acid level (Panel B in S1 Fig). However, when including human GPR50 in the protein alignment, it is apparent that the platypus is much more similar to chicken and from than to human GPR50 (Fig 2A). Furthermore, phylogenetic analysis (including a range of eutherian and marsupial species) shows the platypus grouping with chicken and frog to the exclusion of all therian species (Fig 2B). This suggests that platypus does in fact have the ancestral Mel1c receptor and that the “mammalian” GPR50 evolved after monotremes diverged from therian mammals ~190 MYA. Of interest in the present study is the similarity of the melatonin binding Mel1c sequence throughout the millions of years of evolution between amphibians, birds and even monotremes (Panel B in S1 Fig). In stark contrast is the highly variable GPR50 in therian mammals that have lost their ability to bind melatonin. This strongly suggests that the initial loss of binding allowed GPR50 to then continue to diverge in therian mammals.

There is still a lack of consensus on which specific amino acid changes in GPR50 have caused the lack of binding of this receptor to melatonin, despite many mutation and modeling studies on functional melatonin receptors (see Pala *et al*., for review [35]). The majority of studies have focused on the transmembrane domains. However, Clement *et al*. proposed that the second extracellular loop (E2) in MT_1_ is instead important for melatonin binding [26]. They found that Gln181 in hMT_1_ was highly important for melatonin binding through a stabilizing interaction with Phe179. All melatonin-binding receptors investigated had Gln at this position, while human GPR50 instead had a Thr. Upon examination of the same E2 region of Mel1c in the platypus, chicken and frog we found there also to be a Gln at this same position (Fig 2C), while all eutherian species at this position contained Thr and the three marsupial species analysed had Thr, His or Pro (Fig 2C). This supports the proposal from Clement et al [26] that if Gln in the E2 region of melatonin receptors is important for binding melatonin, then the change of this amino acid to Thr (or His/Pro) may be responsible for the inability of GPR50 to be a functional melatonin receptor. As the marsupial species also lacked Gln, this further suggests that Mel1c changing to GPR50 occurred after the divergence of monotremes and before the therian split, giving a tighter timeframe of 190–160MYA. Further support for this hypothesis is that the marsupial GPR50 also has the characteristic elongated C-tail that eutherian GPR50 has [7]. Functional studies using marsupial GPR50 would need to be undertaken to verify its inability to bind melatonin before committing to this timeframe.

In order to confirm the observations we found in the amino acid sequence for platypus melatonin receptor, we cloned platypus Mel1c for the first time and expressed it to study its binding capacity and function. The three Mel1c receptors studied here (from frog, chicken and platypus) were expressed at the cell surface. Considering the binding aspects, few data have been reported in the literature on the pharmacological profiles of receptors from uncommon species. Chicken and clawed frog Mel1c have been known for some time to bind melatonin [8,36]. To the best of our knowledge, few data have been published on the binding characteristics of Mel1c receptors from other species, and even less has been published regarding various molecules in a binding assay [9,10]. Pharmacological profiles have been published, but using a pigment aggregation *X*. *laevis* melanophore-based assay (i.e., a functional assay) rather than a molecular one [9,11]. Overall, with the few compounds that have been described in clawed frog Mel1c [36] and in chicken Mel1c [9,11], no major discrepancies were recorded here. Though the present work provides new information on the molecular pharmacology of platypus Mel1c, the ancestor of the GPR50 orphan receptor in mammals, the available data on the roles of those two receptors remains scarce. Interestingly, GPR50 is expressed strongly in specific brain areas (hypothalamo-pituitary regions [37–40] and ependymal cell layer of the third ventricle [41]) in many mammalian species, and Mel1c is widely expressed in the chicken brain [8]. Because we cloned the receptor from whole brain, one can conclude that Mel1c is also expressed broadly in this organ in the platypus. Furthermore, from the molecular pharmacology point of view, Mel1c is closer to MT_1_ than to MT_2_; its profile based on a set of 19 compounds as diverse as possible from the melatoninergic prospect is similar to human MT_1_.

Complementing the initial work of Dufourny *et al*. [7], mammalian Mel1c from the platypus fills a gap in terms of sequence and gene structure, as well as molecular pharmacology characteristics, between the melatoninergic systems of amphibians and birds and that of mammals. This work also complements nicely our current efforts to clone melatonin receptors from various species to better understand their sequence/pharmacology relationships [27,28,42,43].

In conclusion, the present work establishes for the first time that melatonin binding to GPR50 was lost after the divergence of monotremes and that this even likely sparked rapid divergence of GPR50 in therian mammals

## Supporting information

S1 Fig Platypus Mel1c gene in comparison with chicken and the clawed frog. Structure of Mel1c in platypus. The entire gene is 34,831 bp in length, where ~30,000 bp intron separates two exons: exon 1 is 184 bp long and exon 2 is 866 bp long. Every 5000 bp are labeled. B) Protein alignment of Mel1c from platypus (*O*. *anatinus*), chicken (*G*. *gallus*) and the clawed frog (*X*. *laevis*), showing that Mel1c is very conserved at the amino acid level. Each amino acid is depicted by its single letter symbol and an associated colour. Identity bar above alignment denotes the similarity at each amino acid position between the four species: green = all homologous; yellow = two species homologous; no bar = no homology. Every 10 amino acid positions are labeled. C) DNA alignment of Mel1c exons from platypus (*O*. *anatinus*), chicken (*G*. *gallus*) and the clawed frog (*X*. *laevis*), showing that at a nucleotide level Mel1c has more variation compared to at the amino acid level. Grey bars indicate homology between all three species while colours indicate SNPs (Single Nucleotide Polymorphisms); red = A; green = T; yellow = G; purple = C. Identity bar above alignment denotes the similarity at each nucleotide position between the three species: green = all homologous; yellow = two species homologous; no bar = no homology. Every 20 bp are labeled.
(TIF)

S1 Data As per the new policy from Plos One, the raw data are available from the supporting information tables. They are counter listings, formatted to give the individual numbers used to calculate all the saturation curves and affinities reported in the present paper. The plates are all arranged the same ways: Saturation: The 3 first columns are used for increasing low concentrations (nM): A: 0.01; B: 0.02; C: 0.04; D: 0.05; E: 0.08; F: 0.1; G: 0.2; in triplicate. The 3 next columns (4 to 6) were used for the nonspecific binding. The 3 next columns (7 to 9) were used for higher concentrations: A: 0.3; B: 0.4; C: 0.5; D: 0.8; E: 1; F: 1.5 and G: 2. The last 3 columns, same concentrations, nonspecific binding. Nonspecific binding was done in the presence of 10 μM of cold melatonin. The H line was not used. R: 11 concentrations of each product. The concentrations of the products were from 10-^14^M to 10^-4^M (from column 1 to 11). Colum 12 is for unspecific binding. Two lines (A&B; C&D, etc.) were used per compounds. For DR in COS7 cell membranes, only 8 compounds were tested in that order from top to bottom: melatonin, 2-iodomelatonin, S 70254, 4P-P-DOT, S 20098/agomelatonin, S 22153, FLN68/ramelteon and Luzindole. For DR in CHO cell membranes in that order from top to bottom: melatonin, 2-iodomelatonin, 6-chlmromeltonin, Luzindole, 4PPDOT, S 20098/agomelatin, FLN68/ramelteon, D600, S20928, S21278, S22153, S70254, S73893, S75436, S27128, DIV880, SD6, SD1881, SD1882 and SD1918. If needed, more information can be obtained from the corresponding author upon request. **Table A. Raw data for calculation of COS7 Xenopus Mel1c (n = 1 & 2) saturations. Table B. Raw data for calculation of COS7 Platypus Mel1c (n = 1) saturation. Table C. Raw data for calculation of COS7 Platypus (n = 2) & Xenopus (n = 3) Mel1c saturations. Table D. Raw data for calculation of COS7 Mel1c Platypus (n = 3) and naïve cells saturation. Table E. Raw data for calculation of CO7 Mel1c Chicken (n = 1 & 2) and naïve cells saturations. Table F. Raw data for calculation of CHO Mel1c Xenopus (n = 1) saturation. Table G. Raw data for calculation of CHO Mel1c Platypus (n = 1 & 2) saturation. Table H. Raw data for calculation of CHO Mel1c Platypus (n = 3), Human MT1 and MT2 & Xenopus Mel1c (n = 2) saturation. Table I. Raw data for calculation of COS7 Mel1c Xenopus saturation (n = 4 & 5) and dose-responses (n = 1 & 2). Table J. Raw data for calculation of COS7 Mel1c Platypus (n = 1 & 2) dose-responses. Table K. Raw data for calculation of COS7 Chicken Mel1c (n = 1 & 2) dose-responses. Table L. Raw data for calculation of CHO Mel1C Platypus (n = 2) & Xenopus (n = 2) dose-responses. Table M. Raw data for calculation CHO Mel1C Platypus (n = 1) & Xenopus (n = 1) dose-responses.**
(ZIP)
