# Supplementary figures and images for: Characterization of the Mel1c melatoninergic receptor in platypus (Ornithorhynchus anatinus)
Source: PLoS One. 2018 Mar 12;13(3):e0191904. doi: 10.1371/journal.pone.0191904 (PMC5846726; doi:10.1371/journal.pone.0191904)

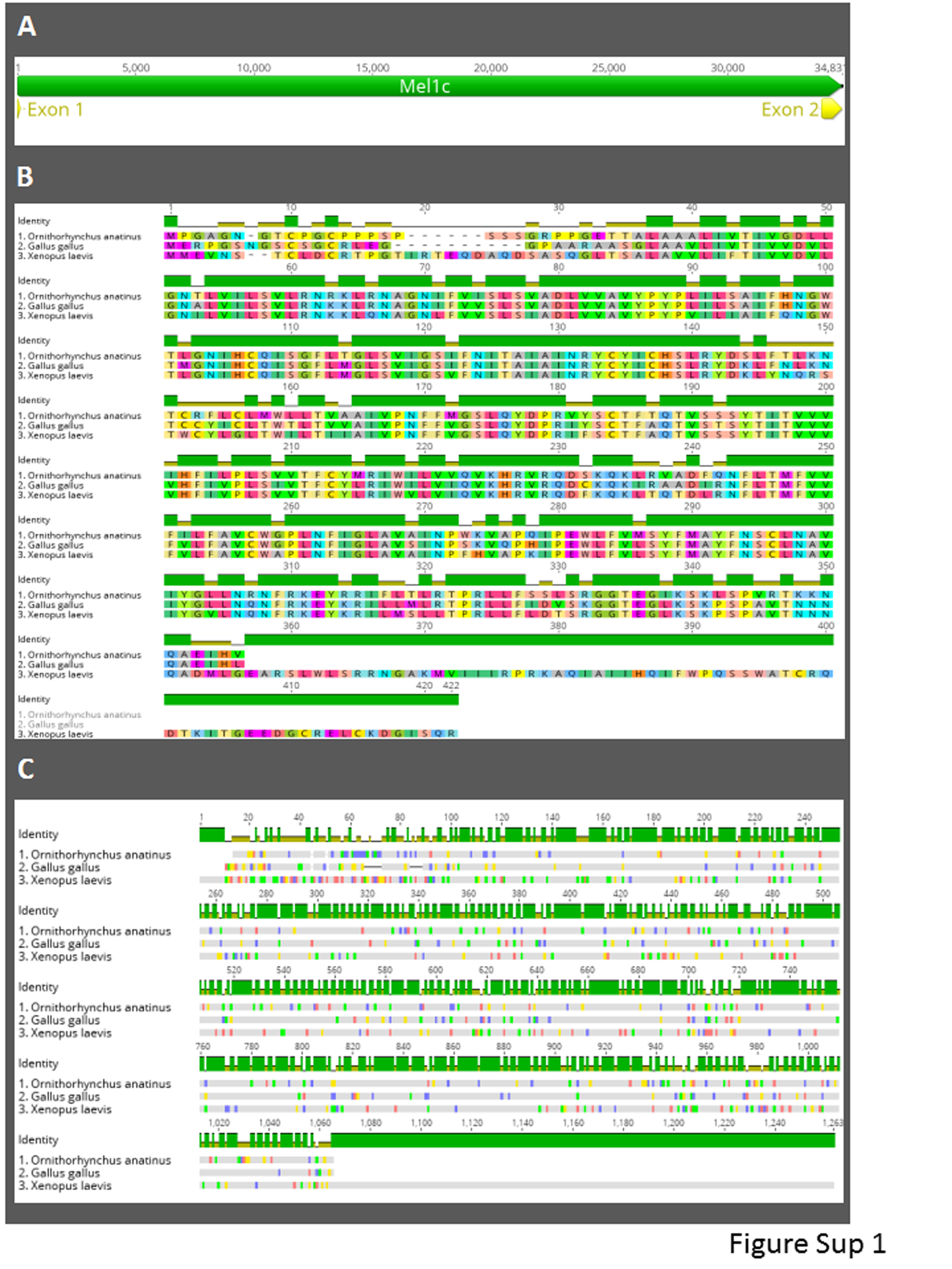

Supplement: S1 Fig — Structure of Mel1c in platypus. The entire gene is 34,831 bp in length, where ~30,000 bp intron separates two exons: exon 1 is 184 bp long and exon 2 is 866 bp long. Every 5000 bp are labeled. B) Protein alignment of Mel1c from platypus (O. anatinus), chicken (G. gallus) and the clawed frog (X. laevis), showing that Mel1c is very conserved at the amino acid level. Each amino acid is depicted by its single letter symbol and an associated colour. Identity bar above alignment denotes the similarity at each amino acid position between the four species: green = all homologous; yellow = two species homologous; no bar = no homology. Every 10 amino acid positions are labeled. C) DNA alignment of Mel1c exons from platypus (O. anatinus), chicken (G. gallus) and the clawed frog (X. laevis), showing that at a nucleotide level Mel1c has more variation compared to at the amino acid level. Grey bars indicate homology between all three species while colours indicate SNPs (Single Nucleotide Polymorphisms); red = A; green = T; yellow = G; purple = C. Identity bar above alignment denotes the similarity at each nucleotide position between the three species: green = all homologous; yellow = two species homologous; no bar = no homology. Every 20 bp are labeled. (TIF) [file pone.0191904.s001.tif]
