# Supplementary material for: Characterization of the Mel1c melatoninergic receptor in platypus (Ornithorhynchus anatinus)
Source: PLoS One. 2018 Mar 12;13(3):e0191904. doi: 10.1371/journal.pone.0191904 (PMC5846726; doi:10.1371/journal.pone.0191904)
Supplement: S1 Data — They are counter listings, formatted to give the individual numbers used to calculate all the saturation curves and affinities reported in the present paper. The plates are all arranged the same ways: Saturation: The 3 first columns are used for increasing low concentrations (nM): A: 0.01; B: 0.02; C: 0.04; D: 0.05; E: 0.08; F: 0.1; G: 0.2; in triplicate. The 3 next columns (4 to 6) were used for the nonspecific binding. The 3 next columns (7 to 9) were used for higher concentrations: A: 0.3; B: 0.4; C: 0.5; D: 0.8; E: 1; F: 1.5 and G: 2. The last 3 columns, same concentrations, nonspecific binding. Nonspecific binding was done in the presence of 10 μM of cold melatonin. The H line was not used. R: 11 concentrations of each product. The concentrations of the products were from 10-14M to 10-4M (from column 1 to 11). Colum 12 is for unspecific binding. Two lines (A&B; C&D, etc.) were used per compounds. For DR in COS7 cell membranes, only 8 compounds were tested in that order from top to bottom: melatonin, 2-iodomelatonin, S 70254, 4P-P-DOT, S 20098/agomelatonin, S 22153, FLN68/ramelteon and Luzindole. For DR in CHO cell membranes in that order from top to bottom: melatonin, 2-iodomelatonin, 6-chlmromeltonin, Luzindole, 4PPDOT, S 20098/agomelatin, FLN68/ramelteon, D600, S20928, S21278, S22153, S70254, S73893, S75436, S27128, DIV880, SD6, SD1881, SD1882 and SD1918. If needed, more information can be obtained from the corresponding author upon request. Table A. Raw data for calculation of COS7 Xenopus Mel1c (n = 1 & 2) saturations. Table B. Raw data for calculation of COS7 Platypus Mel1c (n = 1) saturation. Table C. Raw data for calculation of COS7 Platypus (n = 2) & Xenopus (n = 3) Mel1c saturations. Table D. Raw data for calculation of COS7 Mel1c Platypus (n = 3) and naïve cells saturation. Table E. Raw data for calculation of CO7 Mel1c Chicken (n = 1 & 2) and naïve cells saturations. Table F. Raw data for calculation of CHO Mel1c Xenopus (n = 1) saturation. Table G. [file pone.0191904.s002.zip › Table B.pdf]

Plaque filtre - Plat

DPM	1	2	3	4	5	6	7	8	9	10	11	12
A	2302	2469	1760	0	0	89	9396	8553	8848	397	491	0
B	3420	3102	3083	0	104	106	7926	8787	8262	504	326	919
C	4531	3803	3925	303	705	114	8080	8366	7892	470	400	404
D	4942	4367	4060	565	172	136	9048	8546	8314	625	610	541
E	5778	5036	4847	260	128	172	9466	9861	8955	978	646	570
F	6322	5149	5244	247	288	142	9181	8692	8492	1051	809	756
G	7414	6339	6982	256	216	230	8482	10133	9881	1390	1290	988
H	51	52	67	0	0	47	94	69	60	917	39	0

CPM	1	2	3	4	5	6	7	8	9	10	11	12
A	1378	1490	1033	133	44	45	5693	5203	5353	220	276	9635
B	2042	1849	1859	120	47	57	4804	5284	4999	276	188	539
C	2707	2256	2364	109	54	57	4881	5027	4758	266	223	227
D	2925	2606	2456	88	52	67	5413	5132	5025	367	351	302
E	3457	3002	2923	124	60	91	5708	5922	5391	567	378	333
F	3778	3048	3188	124	100	79	5534	5207	5121	616	474	435
G	4389	3768	4216	127	80	133	5064	6034	5960	814	752	577
H	25	26	24	51	23	26	46	36	32	46	20	19

tSIS	1	2	3	4	5	6	7	8	9	10	11	12
A	85.83	88.26	79.47	10.94	18.63	56.42	89.48	90.81	89	65.34	66.82	13.81
B	85.01	84.47	87.92	15.14	48.06	61.84	89.6	87.01	89.04	63.88	69.83	76.46
C	85.15	83.08	87.48	39.17	24.36	55.79	88.51	86.75	87.86	67.57	66.08	66.73
D	82.33	84.93	88.97	26.66	34.43	54.53	85.56	86.57	88.69	76.2	69.39	65.97
E	85.61	84.59	87.91	52.46	50.54	61.01	87.95	86.62	87.37	70.51	74.34	73.68
F	85.25	82.37	90.66	56.78	38.15	65.69	87.76	85.96	87.95	76.76	74.19	69.48
G	82.37	83.73	88.36	55.62	40.15	70.84	84.96	84.25	87.98	75.59	72.5	73.65
H	52.86	55.71	38.49	8.85	7.38	66.78	54.73	58.65	60.09	23.53	58.22	11.02

Optiplate -totaux

DPM	1	2	3	4	5	6	7	8	9	10	11	12
A	0	0	145	0	364591	358228	369862	3735	1345	13384	13150	13911
B	0	0	44	0	527380	517217	546001	6040	2076	26222	26395	26701
C	0	0	29	0	568185	570951	583032	7881	2851	44456	46457	46810
D	0	0	0	939	935007	1001009	1009469	10529	3585	56535	55860	59737
E	0	0	0	0	1281773	1170733	1294246	13875	4421	91691	92234	92492
F	0	0	29	0	1924999	1949638	1972361	16692	4628	106559	114381	109912
G	0	0	0	0	2523473	2611757	2609611	14990	3877	220904	220555	225230
H	0	0	24	0	16179	20598	16638	8050	3346	2897	2238	1922

CPM	1	2	3	4	5	6	7	8	9	10	11	12
A	9	9	7	119	213527	209948	216695	2305	828	7840	7205	7028
B	9	10	13	174	309064	302627	319645	3705	1273	15364	14315	14038
C	13	14	17	185	333059	334703	341541	4820	1753	25947	25116	23618
D	9	11	14	244	550706	590118	591451	6456	2202	33118	30640	30592

E	14	11	18	367	763113	694169	761544	8492	2712	53725	50513	46614
F	11	15	18	579	1154014	1170788	1178039	10243	2854	62467	64751	57822
G	6	6	17	677	1527328	1578312	1572057	9202	2394	129437	124942	116786
H	6	16	14	254	9973	12708	10166	4925	2058	1789	1338	1122

tSIS	1	2	3	4	5	6	7	8	9	10	11	12
A	6.54	5.43	23.47	8.83	74.98	77.72	75.37	95.65	94.79	75.19	64.03	57.03
B	14.44	6.4	33.42	15.43	75.75	74.24	74.64	93.62	93.66	75.42	63.04	60.22
C	7.34	7.43	85.71	11.77	77.29	76.93	75.22	92.61	94.37	72.91	62.73	56.93
D	6.24	8.66	9.24	30.97	80.64	80.97	78.16	93.46	94.11	75.2	64.15	58.07
E	10.63	7.36	8.44	14.32	84.17	82.92	80.28	92.87	93.64	75.5	63.99	56.84
F	8.2	6.34	90.71	16.09	86.14	86.6	85.11	93.73	95.44	76.63	67.57	60.27
G	8.12	9.5	8.26	14.12	89.14	88.63	87.59	93.86	95.8	75.51	67.65	59.06
H	6.02	9.44	80.24	15.17	95.27	95.53	92.31	92.7	94.45	95.82	85.42	73.19
