# Supplementary material for: Characterization of the Mel1c melatoninergic receptor in platypus (Ornithorhynchus anatinus)
Source: PLoS One. 2018 Mar 12;13(3):e0191904. doi: 10.1371/journal.pone.0191904 (PMC5846726; doi:10.1371/journal.pone.0191904)
Supplement: S1 Data — They are counter listings, formatted to give the individual numbers used to calculate all the saturation curves and affinities reported in the present paper. The plates are all arranged the same ways: Saturation: The 3 first columns are used for increasing low concentrations (nM): A: 0.01; B: 0.02; C: 0.04; D: 0.05; E: 0.08; F: 0.1; G: 0.2; in triplicate. The 3 next columns (4 to 6) were used for the nonspecific binding. The 3 next columns (7 to 9) were used for higher concentrations: A: 0.3; B: 0.4; C: 0.5; D: 0.8; E: 1; F: 1.5 and G: 2. The last 3 columns, same concentrations, nonspecific binding. Nonspecific binding was done in the presence of 10 μM of cold melatonin. The H line was not used. R: 11 concentrations of each product. The concentrations of the products were from 10-14M to 10-4M (from column 1 to 11). Colum 12 is for unspecific binding. Two lines (A&B; C&D, etc.) were used per compounds. For DR in COS7 cell membranes, only 8 compounds were tested in that order from top to bottom: melatonin, 2-iodomelatonin, S 70254, 4P-P-DOT, S 20098/agomelatonin, S 22153, FLN68/ramelteon and Luzindole. For DR in CHO cell membranes in that order from top to bottom: melatonin, 2-iodomelatonin, 6-chlmromeltonin, Luzindole, 4PPDOT, S 20098/agomelatin, FLN68/ramelteon, D600, S20928, S21278, S22153, S70254, S73893, S75436, S27128, DIV880, SD6, SD1881, SD1882 and SD1918. If needed, more information can be obtained from the corresponding author upon request. Table A. Raw data for calculation of COS7 Xenopus Mel1c (n = 1 & 2) saturations. Table B. Raw data for calculation of COS7 Platypus Mel1c (n = 1) saturation. Table C. Raw data for calculation of COS7 Platypus (n = 2) & Xenopus (n = 3) Mel1c saturations. Table D. Raw data for calculation of COS7 Mel1c Platypus (n = 3) and naïve cells saturation. Table E. Raw data for calculation of CO7 Mel1c Chicken (n = 1 & 2) and naïve cells saturations. Table F. Raw data for calculation of CHO Mel1c Xenopus (n = 1) saturation. Table G. [file pone.0191904.s002.zip › Table C.pdf]

Raw data Mel1C Plat Xeno

Mel1C Plat N=1

	1	2	3	4	5	6	7	8	9	10	11	12
A	3751	3574	3915	10347	0	253	13854	12813	13584	460	607	557
B	5446	5532	6114	260	395	203	13967	14309	13693	569	681	561
C	8294	8222	8031	194	196	239	15316	13743	14018	757	701	650
D	8662	9098	8574	151	210	202	15526	14930	13701	850	890	851
E	9801	10424	11014	182	238	283	15148	15121	12895	988	1035	944
F	10216	10889	11704	287	253	267	14219	14241	13182	1385	1380	1297
G	11973	12667	12911	382	340	351	12974	12318	11871	1678	2281	1574
H	122	110	79	71	0	121	145	163	137	117	0	0

	1	2	3	4	5	6	7	8	9	10	11	12
A	1977	2045	2160	57	70	86	7390	7029	7454	215	264	264
B	2778	2979	3305	58	85	85	7492	7737	7484	271	299	270
C	4363	4642	4438	82	82	92	8071	7522	7560	375	303	330
D	4461	4990	4640	65	82	97	7852	8074	7447	424	424	445
E	5077	5797	6026	85	109	118	7847	8231	7161	501	468	495
F	5385	5994	6436	131	104	127	7296	7518	7440	699	681	675
G	6239	7072	7151	166	160	172	6801	6538	6342	848	1100	821
H	49	46	40	29	33	28	50	56	61	38	41	34

	1	2	3	4	5	6	7	8	9	10	11	12
A	71.65	82.17	77.14	14.33	12.07	41.38	73	76.4	76.44	60.54	54.72	61.7
B	68.18	74.09	74.56	29.92	29.43	51.75	73.66	74.6	75.93	61.88	55.4	62.57
C	71.42	80.23	77.33	52.31	51.33	46.49	71.61	76.11	74.29	65.39	54.3	67.66
D	69.13	76.38	74.7	54.38	47.58	62.63	67.32	74.63	75.24	65.97	61.98	70.75
E	69.74	78.15	76.05	60.52	59.11	51.37	69.75	75.43	77.98	67.61	57.71	70.92
F	71.65	76.83	76.7	58.49	50.72	61.87	68.76	71.82	80.18	67.1	65.05	70.35
G	70.38	78.7	77.64	54.48	60.6	64.25	71.03	72.43	73.17	67.23	62.97	70.59
H	49.38	51.48	68.66	51.61	8.03	30.71	41.75	41.48	57.13	39.29	7.82	9.63

Xeno Mel1C N=2

	1	2	3	4	5	6	7	8	9	10	11	12
A	749	714	716	165	0	0	2939	2948	2714	222	279	263
B	1139	1141	1191	2461	0	232	3240	2988	3048	325	406	345
C	1645	1807	1797	99	254	134	3419	3511	3289	373	421	416
D	1930	1848	2055	112	148	119	3768	3539	3384	556	652	547
E	2668	2581	2819	117	152	169	4534	4268	3833	851	716	752
F	2372	2516	2533	114	170	111	3323	3427	3148	932	884	854
G	2809	2873	3080	163	201	264	3212	2902	3019	1158	1240	1099
H	122	0	0	40	0	0	952	162	135	110	0	0

	1	2	3	4	5	6	7	8	9	10	11	12
A	357	405	331	16	31	31	1629	1668	1526	70	145	125
B	545	591	586	26	38	39	1684	1654	1690	170	197	166

C	821	1010	950	31	46	41	1819	1886	1797	180	199	210
D	953	1011	1083	36	41	48	1931	1934	1836	293	313	266
E	1333	1422	1455	46	48	72	2305	2250	2107	413	369	395
F	1164	1413	1406	53	60	53	1801	1814	1737	479	449	433
G	1399	1543	1691	69	95	121	1743	1576	1648	623	657	574
H	18	18	19	13	21	8	27	21	31	13	24	20

	1	2	3	4	5	6	7	8	9	10	11	12
A	62.02	80.84	59.48	21.19	7.84	11.94	77.74	80.55	79.63	38.49	70.08	61.99
B	62.29	69.75	64.79	14.7	11.87	26.1	70.11	77.52	77.74	70.66	63.64	63.04
C	66.03	78.76	71.92	38.4	27.12	37.69	72.72	73.85	75.87	63.3	61.16	67.31
D	65.03	76.05	71.63	39.51	34.93	49.39	68.64	75.88	74.99	71.75	62.48	63.7
E	66.15	76.94	69.34	47.54	38.92	53.16	67.85	71.65	76.63	63.59	69.07	71.12
F	64.49	79.5	77.91	59.14	42.63	62.96	74.9	72.09	77.15	68.98	67.62	67.65
G	65.9	73.81	76.5	53.31	61.42	58.61	75.05	75.13	75.75	74.07	72.32	70.57
H	24.86	8.84	9.64	39.08	6.87	11.19	16.01	23.59	30.48	22.69	6.7	8.97

Totaux

	1	2	3	4	5	6	7	8	9	10	11	12
A	0	22	0	491292	470268	481494	3255	1110	710	16858	19844	17841
B	0	0	0	666958	645904	657727	5278	1641	1148	34068	36814	32929
C	0	0	27766	814446	793509	821327	7317	2255	1500	68003	73399	66622
D	0	0	0	1291827	1280779	1315453	10297	2806	1923	63203	90313	82203
E	0	0	0	1614311	1655972	1519747	13029	3563	2313	128152	142648	132711
F	0	0	0	2396478	2405678	2466111	16394	3595	2593	160139	171662	159587
G	0	0	0	3069452	3172562	3344799	15358	2992	2533	306127	321880	301904
H	0	0	0	16073	19911	16855	7691	2805	1694	1834	2244	1793

	1	2	3	4	5	6	7	8	9	10	11	12
A	14	13	38	263664	253634	254577	1897	667	423	8317	9186	8240
B	16	20	110	358467	352148	347769	3079	954	672	16820	16765	15237
C	17	26	123	432332	432226	438547	4263	1350	899	33478	33437	29898
D	22	25	194	706107	705353	709670	6005	1661	1141	31191	41062	37983
E	22	38	286	885167	915442	829099	7554	2094	1366	62353	65964	58659
F	14	39	431	1340270	1371233	1364727	9447	2098	1513	76866	79162	71445
G	20	46	416	1702074	1794233	1833419	8882	1738	1475	147799	151249	135585
H	16	29	126	9479	12082	9835	4490	1624	998	1052	1220	993

	1	2	3	4	5	6	7	8	9	10	11	12
A	7.38	80.32	9.21	73.72	74.31	71.99	84.97	89.47	88.36	64.99	59.59	59.4
B	7.48	10.72	13.65	73.89	75.62	72	85.12	84.61	85.6	65.06	58.32	59.56
C	7.46	10.57	14.25	72.44	75.51	73.12	84.88	89.06	89.12	64.8	58.34	57.13
D	6.39	8.65	12.54	75.94	76.89	74.34	85.06	87.43	87.81	65.02	58.19	59.44
E	9.82	11.94	13.55	76.34	77.38	75.7	84.14	86.32	87.11	63.75	59.51	55.89
F	8.63	11.15	13.44	78.92	81.58	77.52	83.2	85.17	85.17	62.57	59.29	56.93
G	9.68	11.29	13.46	77.79	80.46	76.3	83.75	84.43	84.83	63.07	60.79	57.19
H	8.48	8.58	13.67	86.9	90.95	85.14	85.22	83.89	86.76	82.53	75.28	77.6
