# Supplementary material for: Characterization of the Mel1c melatoninergic receptor in platypus (Ornithorhynchus anatinus)
Source: PLoS One. 2018 Mar 12;13(3):e0191904. doi: 10.1371/journal.pone.0191904 (PMC5846726; doi:10.1371/journal.pone.0191904)
Supplement: S1 Data — They are counter listings, formatted to give the individual numbers used to calculate all the saturation curves and affinities reported in the present paper. The plates are all arranged the same ways: Saturation: The 3 first columns are used for increasing low concentrations (nM): A: 0.01; B: 0.02; C: 0.04; D: 0.05; E: 0.08; F: 0.1; G: 0.2; in triplicate. The 3 next columns (4 to 6) were used for the nonspecific binding. The 3 next columns (7 to 9) were used for higher concentrations: A: 0.3; B: 0.4; C: 0.5; D: 0.8; E: 1; F: 1.5 and G: 2. The last 3 columns, same concentrations, nonspecific binding. Nonspecific binding was done in the presence of 10 μM of cold melatonin. The H line was not used. R: 11 concentrations of each product. The concentrations of the products were from 10-14M to 10-4M (from column 1 to 11). Colum 12 is for unspecific binding. Two lines (A&B; C&D, etc.) were used per compounds. For DR in COS7 cell membranes, only 8 compounds were tested in that order from top to bottom: melatonin, 2-iodomelatonin, S 70254, 4P-P-DOT, S 20098/agomelatonin, S 22153, FLN68/ramelteon and Luzindole. For DR in CHO cell membranes in that order from top to bottom: melatonin, 2-iodomelatonin, 6-chlmromeltonin, Luzindole, 4PPDOT, S 20098/agomelatin, FLN68/ramelteon, D600, S20928, S21278, S22153, S70254, S73893, S75436, S27128, DIV880, SD6, SD1881, SD1882 and SD1918. If needed, more information can be obtained from the corresponding author upon request. Table A. Raw data for calculation of COS7 Xenopus Mel1c (n = 1 & 2) saturations. Table B. Raw data for calculation of COS7 Platypus Mel1c (n = 1) saturation. Table C. Raw data for calculation of COS7 Platypus (n = 2) & Xenopus (n = 3) Mel1c saturations. Table D. Raw data for calculation of COS7 Mel1c Platypus (n = 3) and naïve cells saturation. Table E. Raw data for calculation of CO7 Mel1c Chicken (n = 1 & 2) and naïve cells saturations. Table F. Raw data for calculation of CHO Mel1c Xenopus (n = 1) saturation. Table G. [file pone.0191904.s002.zip › Table D.pdf]

Plaque filtre - Plat2

DPM	1	2	3	4	5	6	7	8	9	10	11
A	3816	3063	2870	0	187	109	12296	11894	11278	362	226
B	5345	5634	5894	524	106	130	13073	12358	12827	451	298
C	5931	6888	6771	175	88	137	15354	14095	13653	535	340
D	7398	7297	7023	206	109	167	14603	14366	14036	650	565
E	8446	8263	8123	239	139	182	15003	16122	14372	831	580
F	8380	8936	8775	234	203	205	14636	15258	15720	995	907
G	9573	10185	11047	230	154	213	13986	13993	16460	1286	1116
H	79	82	67	0	56	67	88	89	118	99	43

CPM	1	2	3	4	5	6	7	8	9	10	11
A	2284	1823	1751	102	34	52	7476	7215	6787	201	132
B	3224	3375	3572	79	47	70	7835	7482	7752	250	164
C	3518	4123	4082	83	50	78	9275	8532	8301	311	199
D	4427	4397	4284	97	57	84	8856	8689	8565	381	331
E	5143	4978	4990	105	81	100	9052	9795	8425	487	340
F	4950	5365	5379	114	87	96	8816	9198	9459	585	531
G	5725	6135	6715	122	86	120	8383	8291	9943	753	653
H	35	43	37	50	22	32	46	48	61	46	24

tSIS	1	2	3	4	5	6	7	8	9	10	11
A	85.73	84.04	91.74	12.81	27.39	52.87	90.65	89.88	87.27	65.32	71.77
B	88.04	85.96	89.57	26.53	46.86	63	86.05	89.24	88.65	64.98	64.59
C	83.08	85.76	87.88	51.67	66.26	68.15	88.51	89.19	90.63	71.27	75.65
D	85.65	87.64	91.74	50.87	60.03	57.25	89.79	88.93	91.86	79.22	74.2
E	91.13	87.61	94.14	46.68	74.17	64.69	88.11	90.39	76.68	76.37	75.36
F	81.69	86.53	93.38	54.04	45.25	50.48	87.55	87.82	87.2	80.09	74.38
G	85.44	87.55	90.54	61.36	66.34	67.31	86.11	82.68	88.49	78.3	74.15
H	47.89	59.53	63.81	20.89	42.29	51.34	59.88	61.73	58.18	49.27	63.71

Plaque filtre - naive cells

DPM	1	2	3	4	5	6	7	8	9	10	11
A	0	0	23	0	0	0	329	155	296	219	167
B	0	0	60	0	0	0	238	227	256	295	229
C	50	29	0	0	173	126	402	286	317	396	295
D	0	0	41	0	0	89	635	481	540	448	427
E	62	123	74	0	92	91	616	655	574	621	408
F	70	80	85	269	116	102	793	759	880	716	558
G	107	144	82	333	127	142	833	748	749	1151	803
H	0	0	34	0	0	0	0	0	15	0	0

CPM	1	2	3	4	5	6	7	8	9	10	11
A	8	14	11	53	25	12	152	90	157	118	82
B	17	23	14	41	22	19	133	133	148	162	124
C	23	18	19	59	30	19	222	163	176	210	150
D	19	24	23	55	36	27	369	275	313	260	247

E	30	40	40	61	40	33	358	374	329	349	236
F	37	41	44	73	52	47	428	440	487	417	324
G	56	76	45	84	59	66	475	434	429	675	459
H	9	6	9	33	18	15	11	12	11	29	13

tSIS	1	2	3	4	5	6	7	8	9	10	11
A	7.23	9.18	50.09	9.07	7.12	8.47	49.72	73.42	60.69	62.16	54.39
B	21.24	16.25	28.77	7.84	12.39	14.58	65.6	74.14	69.9	64.29	62.81
C	49.51	101.97	20.73	14.34	27.15	26.45	64.7	68.72	65.13	61	57.37
D	16.31	15.37	67.56	10.77	20.21	35.08	71.13	68.74	70.57	71.21	70.37
E	53.63	36.37	61.77	14.43	46.67	39.63	71.2	68.55	68.97	66.85	70.19
F	60.49	58.16	58.4	32.13	48.49	49.38	62.42	70.57	64.95	71.82	71.44
G	59.16	60.78	64.24	30.31	50.77	50.17	68.34	70.73	69.01	78.88	68.62
H	6.16	6.27	30.37	7.81	8.77	8.61	5.4	8.14	178.87	19.58	8.47

optiplate-totaux

DPM	1	2	3	4	5	6	7	8	9	10	11
A	0	5	0	0	352157	354573	361682	3847	1375	12355	11552
B	0	0	0	0	490044	492027	491473	6035	2224	31097	32068
C	0	0	0	0	596278	610469	621651	8320	3151	50744	48299
D	0	0	17	0	1018493	1025632	1055164	12164	4102	56196	62095
E	20	0	58	0	1260952	1276922	1325403	16108	4973	88386	88201
F	0	0	25	0	1909774	1947373	2015294	19536	5233	107330	115004
G	10	0	20	0	2533407	2629440	2686376	17897	4418	216297	199348
H	0	0	0	0	18022	22452	18517	8906	3700	2988	2472

CPM	1	2	3	4	5	6	7	8	9	10	11
A	4	5	5	71	206276	207920	212011	2348	840	7208	6167
B	7	6	8	107	287895	288689	288089	3688	1355	18145	17142
C	4	11	6	158	349506	359357	364365	5061	1914	29514	25199
D	9	4	10	202	601195	604615	619462	7402	2518	32675	33201
E	5	12	16	332	745428	756640	782674	9771	3023	51174	47145
F	6	12	18	510	1141931	1166325	1193463	11888	3198	62787	63064
G	6	6	12	659	1526768	1581515	1605347	10888	2715	126485	110564
H	3	11	11	226	11066	13763	11287	5418	2263	1820	1450

tSIS	1	2	3	4	5	6	7	8	9	10	11
A	4.08	254.36	4.07	10.84	78.46	78.94	76.28	91.87	92.27	72.73	61.57
B	9.1	7.41	10.2	11.13	79.68	79.17	77.32	92.32	91.44	72.81	61.68
C	7.96	11.44	4.07	14.29	78.76	80.44	77.55	90.85	90.43	71.6	59.57
D	9.23	5.17	68.02	15.5	81.42	80.96	79.41	90.92	93.85	71.5	61.7
E	29.03	16.8	32.38	16.3	81.93	82.71	81.56	89.89	90.54	70.38	61.68
F	8.45	10.61	147.08	14.16	85.43	85.88	82.52	90.94	92.3	74.11	64.12
G	99.58	6.46	78.19	14.21	87.72	87.07	85.26	90.84	94.28	73.88	65.29
H	4.08	12.96	8.39	13.24	93.95	93.38	91.5	90.81	92.65	91.31	79.02

12
381
269
285
549
541
753
995
84

12
172
157
164
316
316
434
579
24

12
48.19
72.48
69.68
69.68
73.44
69.78
71.77
33.13

12
326
181
257
371
494
589
712
0

12
150
98
129
214

272
341
414
22
12
49.6
63.16
56.75
69.8
64.48
70.58
71.3
13.77
12
12308
30397
37141
59519
86381
112849
214043
1995
12
6390
16009
20023
30934
45404
59356
114034
1129
12
59.16
60.37
62.46
59.25
60.2
60.26
61.38
67.54
