# Supplementary material for: Characterization of the Mel1c melatoninergic receptor in platypus (Ornithorhynchus anatinus)
Source: PLoS One. 2018 Mar 12;13(3):e0191904. doi: 10.1371/journal.pone.0191904 (PMC5846726; doi:10.1371/journal.pone.0191904)
Supplement: S1 Data — They are counter listings, formatted to give the individual numbers used to calculate all the saturation curves and affinities reported in the present paper. The plates are all arranged the same ways: Saturation: The 3 first columns are used for increasing low concentrations (nM): A: 0.01; B: 0.02; C: 0.04; D: 0.05; E: 0.08; F: 0.1; G: 0.2; in triplicate. The 3 next columns (4 to 6) were used for the nonspecific binding. The 3 next columns (7 to 9) were used for higher concentrations: A: 0.3; B: 0.4; C: 0.5; D: 0.8; E: 1; F: 1.5 and G: 2. The last 3 columns, same concentrations, nonspecific binding. Nonspecific binding was done in the presence of 10 μM of cold melatonin. The H line was not used. R: 11 concentrations of each product. The concentrations of the products were from 10-14M to 10-4M (from column 1 to 11). Colum 12 is for unspecific binding. Two lines (A&B; C&D, etc.) were used per compounds. For DR in COS7 cell membranes, only 8 compounds were tested in that order from top to bottom: melatonin, 2-iodomelatonin, S 70254, 4P-P-DOT, S 20098/agomelatonin, S 22153, FLN68/ramelteon and Luzindole. For DR in CHO cell membranes in that order from top to bottom: melatonin, 2-iodomelatonin, 6-chlmromeltonin, Luzindole, 4PPDOT, S 20098/agomelatin, FLN68/ramelteon, D600, S20928, S21278, S22153, S70254, S73893, S75436, S27128, DIV880, SD6, SD1881, SD1882 and SD1918. If needed, more information can be obtained from the corresponding author upon request. Table A. Raw data for calculation of COS7 Xenopus Mel1c (n = 1 & 2) saturations. Table B. Raw data for calculation of COS7 Platypus Mel1c (n = 1) saturation. Table C. Raw data for calculation of COS7 Platypus (n = 2) & Xenopus (n = 3) Mel1c saturations. Table D. Raw data for calculation of COS7 Mel1c Platypus (n = 3) and naïve cells saturation. Table E. Raw data for calculation of CO7 Mel1c Chicken (n = 1 & 2) and naïve cells saturations. Table F. Raw data for calculation of CHO Mel1c Xenopus (n = 1) saturation. Table G. [file pone.0191904.s002.zip › Table E.pdf]

Optiplate -totaux

DPM	1	2	3	4	5	6	7	8	9	10	11	12
A	0	0	14	0	369217	376026	372587	3419	1240	12252	12860	12849
B	0	0	0	0	518328	517289	521945	5500	1858	24003	25447	25106
C	37	0	19	0	647482	656017	656605	7451	2474	32044	48429	48040
D	0	20	0	0	1048097	1055944	1081457	10298	3155	56026	61613	61765
E	0	41	43	1614	1327221	1342843	1331264	12457	3492	92427	96112	95803
F	0	0	0	0	1983073	2016615	2025279	12585	3236	73580	117265	120371
G	0	0	0	0	2623675	2482950	139197	7162	3307	208647	234331	237221
H	0	0	18	0	14720	15656	8145	3797	2222	2248	1927	1657

CPM	1	2	3	4	5	6	7	8	9	10	11	12
A	8	11	7	97	216270	220431	218251	2066	744	7096	6594	5858
B	7	8	9	123	303790	303241	305890	3326	1128	13874	13283	12329
C	8	9	12	146	379455	385230	384909	4518	1503	18776	25602	23017
D	5	10	11	247	624848	627222	640054	6221	1916	32756	33397	29951
E	9	11	21	352	790310	798093	787779	7505	2113	53508	50020	45141
F	6	10	13	509	1200872	1218737	1218158	7610	1975	43121	64355	59882
G	6	10	12	542	1615966	1532579	86755	4324	2017	122057	130323	119773
H	10	7	10	188	9006	9557	4945	2302	1350	1372	1137	924

tSIS	1	2	3	4	5	6	7	8	9	10	11	12
A	13.46	8.35	57.47	10.83	75.13	77.03	75.17	88.57	86.49	70.42	58.17	48.93
B	7.7	7.83	9.19	11.11	78.73	77.05	77.77	88.81	90.15	70.16	59.61	54.54
C	28.54	7.35	99.52	15.51	77.81	79.51	77.06	89.77	90.42	75.52	60.7	52.51
D	9.61	53.35	8.36	12.41	84.58	83.48	82.32	88.5	90.33	73.75	62.98	53.48
E	6.15	30.63	52.38	28.53	84.23	83.65	82.27	87.64	89.11	70.36	59.37	51.24
F	6.99	11.24	9.31	18.12	89.32	88.65	87.08	88.83	91.94	77.82	64.2	55.69
G	13.08	11.72	10.01	15	94.98	95.71	99	88.34	91.63	74.11	65.58	56.98
H	7.52	9.94	65.55	14.39	92.75	91.98	90.2	89.65	90.5	91.99	81.29	65.82

Saturation-Chicken1

DPM	1	2	3	4	5	6	7	8	9	10	11	12
A	3858	4582	4927	150	110	260	58112	57505	59353	538	339	306
B	8482	9249	9517	197	144	292	68910	65482	67023	778	457	425
C	15990	14247	16459	225	203	423	72438	75384	72473	1104	587	469
D	16539	18507	19656	281	185	480	87730	87083	81565	1231	928	699
E	26888	27366	27817	362	263	543	89085	92533	87275	1493	840	798
F	31840	32006	30917	335	280	599	97525	94237	90858	1668	1286	1064
G	42842	46473	47332	440	265	554	93587	93052	87702	2081	1907	1476
H	265	270	271	184	170	204	467	483	491	218	118	102

CPM	1	2	3	4	5	6	7	8	9	10	11	12
A	1864	2694	2936	68	63	146	34442	34265	35553	312	178	145
B	5040	5500	5729	98	77	169	40973	38644	40131	456	264	224
C	9473	8372	9864	131	118	244	43111	44973	43350	634	341	270
D	9564	10912	11747	137	107	276	51963	51671	47955	721	534	404

E	15795	16366	16561	197	150	311	53223	54844	51938	875	475	449
F	18763	18822	18133	185	160	348	57672	55634	54366	976	729	613
G	25115	27638	27874	252	129	315	55589	54762	52011	1210	1045	858
H	132	144	150	100	80	118	258	272	270	127	58	50

tSIS	1	2	3	4	5	6	7	8	9	10	11	12
A	53.17	79.92	84.47	48.87	68.71	66.91	82.78	84.43	85.92	70.86	59.84	51.82
B	83.61	83.84	87.31	55.97	61.95	70.47	83.78	81.34	85.81	75.38	70.13	60.56
C	82.65	79.79	86.06	71.51	72.27	70.09	84.06	84.78	85.53	69.35	71.48	69.38
D	70.21	81.04	85.27	53.94	69.99	69.5	82.57	83.14	79.97	75.61	69.48	70.13
E	79.65	85.48	84.18	63.42	68.75	68.81	85.19	82.79	84.05	76.7	67.57	66.77
F	80.83	80.07	79.01	65.1	68.43	71.16	82.05	81.47	85.62	74.22	67.77	69.74
G	77.04	83.84	80.58	69.23	54.15	68.16	83.47	80.34	82.97	71.5	64.03	71.54
H	55.93	61.51	65.48	62.98	51.51	71.25	65.04	67.13	64.48	72.36	54.41	54.49

Saturation-Chicken2

DPM	1	2	3	4	5	6	7	8	9	10	11	12
A	4716	5239	5110	187	129	271	61564	59735	60182	619	464	367
B	9793	10354	9998	243	200	469	73395	70084	70497	833	476	400
C	16738	17587	17696	263	224	531	72707	78747	70390	942	616	435
D	19502	21165	20496	303	279	548	82890	85264	86178	1237	815	640
E	27712	29427	29012	329	348	749	86569	90438	88662	1531	887	765
F	31798	35357	32924	423	354	565	91744	94295	91304	1825	1183	848
G	48953	48676	49456	490	316	675	88486	90288	90805	1867	1360	1126
H	268	272	256	216	175	251	562	582	593	212	101	185

CPM	1	2	3	4	5	6	7	8	9	10	11	12
A	2748	3064	3018	91	65	149	36307	35401	35873	360	253	194
B	5769	6106	5981	138	117	258	43642	41111	42433	485	276	212
C	9820	10315	10572	145	131	305	42595	46855	41264	546	349	236
D	11433	12557	12227	176	158	308	48579	50111	51788	725	472	375
E	16238	17608	17134	191	189	417	50572	53447	51968	892	518	447
F	18632	21050	19296	243	196	326	53717	55857	54373	1060	688	494
G	28708	28694	28989	278	184	381	51840	52898	54099	1094	780	655
H	141	150	144	114	88	129	311	327	286	118	54	84

tSIS	1	2	3	4	5	6	7	8	9	10	11	12
A	72.27	73.88	81.68	53.42	57.36	64.56	81.11	82.75	84.53	72.13	63.64	60.8
B	80.71	81.09	85.53	67.72	74.18	64.59	83.8	79.07	87.31	71.98	71.06	61.07
C	79.14	79.03	85.17	64.33	73.66	69.15	75.3	84	76.93	70.7	67.83	63.07
D	76.95	83.11	84.76	71.91	67.39	66.85	75.83	79.83	86.79	76.01	70.25	75.29
E	78.02	85.63	81.59	71.52	63.44	65.67	73.34	81.83	77.5	72.45	73.09	73.22
F	78.08	84.17	75.89	69.55	65.18	69.84	74.74	82.61	84.26	71.26	71.68	71.77
G	78.97	80.95	78.77	67.7	73.05	67.14	75.31	78.22	84.38	76	69.17	71.68
H	60.61	64.58	66.76	60.52	55.97	58.31	65.03	66.78	53.11	65.74	62.32	49.04

Saturation-naive cells

DPM	1	2	3	4	5	6	7	8	9	10	11	12
A	169	79	58	0	315	0	223	230	259	269	195	208
B	55	191	48	422	148	73	372	323	363	309	301	255
C	114	53	106	227	76	80	453	572	453	446	262	312
D	66	102	88	755	117	119	947	840	697	755	542	520
E	108	103	103	153	115	166	1057	769	718	859	631	524
F	81	91	155	207	127	130	924	909	1064	1048	677	801
G	185	218	209	208	142	201	1180	1254	1222	1290	949	853
H	125	89	78	0	162	92	57	61	84	0	60	64

CPM	1	2	3	4	5	6	7	8	9	10	11	12
A	20	32	20	42	41	19	115	113	121	127	97	76
B	25	25	28	46	35	30	194	176	188	166	146	126
C	35	31	44	56	38	37	251	307	254	248	145	157
D	30	52	51	53	52	46	531	459	380	413	298	252
E	49	60	50	69	48	42	589	435	410	486	345	280
F	46	53	71	73	66	56	513	504	595	576	376	431
G	96	110	85	105	81	110	663	659	668	725	497	457
H	17	24	32	30	53	46	34	29	24	29	17	31

tSIS	1	2	3	4	5	6	7	8	9	10	11	12
A	25.72	43.06	37.23	15.45	25.97	15.64	58.98	54.26	50.72	51.5	56.08	39.62
B	48.63	26.02	71.98	25.33	29.27	43.71	59.57	63.62	59.14	62.21	53.45	55.2
C	34.8	78.52	44.19	29.74	55.43	50.2	65.11	62.18	66.55	65.58	65.11	56.66
D	48.76	57.47	71.95	24.15	47.8	41.16	66.53	63.69	63.58	63.97	64.43	53.23
E	47.8	71.49	53.62	48.22	44.51	30.48	65.81	67.36	68.59	67.55	63.98	61.57
F	68.53	72.47	49.15	38.6	58.57	45.67	65.48	65.25	66.16	64.34	65.42	62.14
G	59.1	56.93	42.96	57.1	68.3	63.75	66.75	60.21	63.77	66.72	59.86	61.82
H	26.13	31.86	43.19	12.69	36.52	55.83	80.22	52.36	32.86	19.61	32.56	53.77
