# Supplementary material for: Characterization of the Mel1c melatoninergic receptor in platypus (Ornithorhynchus anatinus)
Source: PLoS One. 2018 Mar 12;13(3):e0191904. doi: 10.1371/journal.pone.0191904 (PMC5846726; doi:10.1371/journal.pone.0191904)
Supplement: S1 Data — They are counter listings, formatted to give the individual numbers used to calculate all the saturation curves and affinities reported in the present paper. The plates are all arranged the same ways: Saturation: The 3 first columns are used for increasing low concentrations (nM): A: 0.01; B: 0.02; C: 0.04; D: 0.05; E: 0.08; F: 0.1; G: 0.2; in triplicate. The 3 next columns (4 to 6) were used for the nonspecific binding. The 3 next columns (7 to 9) were used for higher concentrations: A: 0.3; B: 0.4; C: 0.5; D: 0.8; E: 1; F: 1.5 and G: 2. The last 3 columns, same concentrations, nonspecific binding. Nonspecific binding was done in the presence of 10 μM of cold melatonin. The H line was not used. R: 11 concentrations of each product. The concentrations of the products were from 10-14M to 10-4M (from column 1 to 11). Colum 12 is for unspecific binding. Two lines (A&B; C&D, etc.) were used per compounds. For DR in COS7 cell membranes, only 8 compounds were tested in that order from top to bottom: melatonin, 2-iodomelatonin, S 70254, 4P-P-DOT, S 20098/agomelatonin, S 22153, FLN68/ramelteon and Luzindole. For DR in CHO cell membranes in that order from top to bottom: melatonin, 2-iodomelatonin, 6-chlmromeltonin, Luzindole, 4PPDOT, S 20098/agomelatin, FLN68/ramelteon, D600, S20928, S21278, S22153, S70254, S73893, S75436, S27128, DIV880, SD6, SD1881, SD1882 and SD1918. If needed, more information can be obtained from the corresponding author upon request. Table A. Raw data for calculation of COS7 Xenopus Mel1c (n = 1 & 2) saturations. Table B. Raw data for calculation of COS7 Platypus Mel1c (n = 1) saturation. Table C. Raw data for calculation of COS7 Platypus (n = 2) & Xenopus (n = 3) Mel1c saturations. Table D. Raw data for calculation of COS7 Mel1c Platypus (n = 3) and naïve cells saturation. Table E. Raw data for calculation of CO7 Mel1c Chicken (n = 1 & 2) and naïve cells saturations. Table F. Raw data for calculation of CHO Mel1c Xenopus (n = 1) saturation. Table G. [file pone.0191904.s002.zip › Table F.pdf]

Mel1C Xenope Laevis												
DPM	1	2	3	4	5	6	7	8	9	10	11	12
A	424	553	581	0	488	0	3592	1907	1245	175	682	292
B	957	947	979	220	0	0	1087	3313	3316	378	324	289
C	1415	1526	1287	178	2871	322	3793	4349	3962	360	321	500
D	1655	1595	1486	123	118	135	2019	5050	4693	171	309	203
E	1975	1949	2139	309	146	145	5515	4832	4495	698	487	770
F	2208	2220	2276	111	162	174	6557	4763	4457	379	726	304
G	2895	2852	2889	189	158	208	4992	4735	4377	765	780	361
H	298	0	528	97	0	737	163	57	0	0	0	0

CPM	1	2	3	4	5	6	7	8	9	10	11	12
A	154	315	327	27	42	32	2062	752	590	65	52	94
B	505	535	531	23	38	24	466	1807	1881	193	162	86
C	785	873	670	31	46	33	2094	2471	2337	196	161	278
D	897	942	762	45	52	32	923	2932	2641	79	97	84
E	1075	1140	1226	26	63	68	3042	2806	2599	379	199	409
F	1207	1257	1278	57	64	81	3701	2672	2617	154	359	111
G	1596	1670	1600	98	78	111	2830	2775	2530	422	408	165
H	21	16	20	18	29	24	37	25	32	16	28	24

	1	2	3	4	5	6	7	8	9	10	11	12
A	44.01	81.38	79.82	10.19	20.21	13.11	82.61	48.09	61.51	45	19.52	39.3
B	71.76	80.43	75.09	21.52	10.15	8.7	53.59	75.69	80.9	68.27	66.35	36.77
C	77.81	82.05	70.23	26.72	15.09	21.52	77.25	81.09	86.93	75.62	66.62	78.15
D	74.91	87.05	68.71	43.97	55.89	30.85	58.64	84.39	79.76	58.85	38.48	51.01
E	75.5	85.63	82.42	20.12	54.36	60.69	77.11	84.4	83.68	75.17	50.41	72.42
F	75.94	80.58	79.47	68.68	48.43	60.38	80.18	79.33	86.15	49.86	65.11	44.41
G	77.02	85.72	77.6	69.96	64.88	72.89	80.83	85.84	83.7	76.9	70.68	58.68
H	19.03	7.39	16.67	27.79	8.03	16.32	30.36	55.95	10.62	8.59	6.55	8.56

Totaux												
DPM	1	2	3	4	5	6	7	8	9	10	11	12
A	521	769	1563	4249	466177	484588	467950	3977	1505	19083	20251	19241
B	776	1084	2283	6818	658251	671954	668650	6002	2333	38349	39981	37995
C	817	1388	3163	9410	831526	843601	861892	8505	3245	68484	71865	66742
D	919	1641	3917	12898	1334775	1365997	1364986	13118	4214	96564	97238	95000
E	961	1938	4764	16415	1698294	1713906	1689720	17099	5393	139942	140041	137361
F	983	1967	4780	20099	2523661	2565302	2542724	20336	5829	172478	175513	167618
G	806	1683	3632	18093	3225804	3428752	3262250	19226	5038	335746	328499	333528
H	824	1375	3328	8892	20885	23774	22324	10201	4092	3850	3473	2880

CPM	1	2	3	4	5	6	7	8	9	10	11	12
A	315	500	980	2702	272796	282210	249969	2511	961	10071	9903	9491
B	466	702	1442	4336	382723	382024	368933	3810	1486	20045	19752	18510
C	492	903	1983	5942	494104	481313	472673	5365	2065	35790	35169	32485
D	547	1055	2426	8094	778704	795370	748403	8230	2667	49911	47537	46211

E	581	1237	2930	10314	984881	989322	916545	10572	3411	72302	69198	65919
F	585	1240	2964	12385	1464886	1482432	1400556	12483	3608	88120	85775	79360
G	478	1047	2251	11148	1969581	1986751	1876852	11805	3166	173631	170757	158455
H	490	853	2068	5464	13266	14571	13474	6211	2588	2327	1997	1659

	1	2	3	4	5	6	7	8	9	10	11	12
A	90.49	101.19	95.75	97.85	85.61	84.84	73.17	96.8	98.4	71.78	64.19	64.98
B	89.39	100.51	96.83	97.87	84.58	81.21	77.14	97.58	98.1	70.72	65.12	63.86
C	89.85	101.36	95.74	96.79	87.96	81.72	76.36	96.64	97.96	70.7	64.26	63.78
D	88.28	99.54	93.92	95.87	85.12	84.81	76.33	95.83	97.17	69.51	64.17	63.72
E	90.57	98.36	92.9	96.05	84.18	83.46	75	93.68	97.03	69.47	65.14	62.55
F	88.31	96.54	94.09	93.19	84.32	83.63	76.91	92.62	93.84	68.32	64.14	61.41
G	87.83	94.56	94.01	93.17	91.85	84.05	82.96	92.67	96.1	69.57	70.12	61.7
H	87.97	94.15	94.45	92.78	97.7	92.4	90.19	91.44	97.07	90.4	82.82	83.11
