# Supplementary material for: Characterization of the Mel1c melatoninergic receptor in platypus (Ornithorhynchus anatinus)
Source: PLoS One. 2018 Mar 12;13(3):e0191904. doi: 10.1371/journal.pone.0191904 (PMC5846726; doi:10.1371/journal.pone.0191904)
Supplement: S1 Data — They are counter listings, formatted to give the individual numbers used to calculate all the saturation curves and affinities reported in the present paper. The plates are all arranged the same ways: Saturation: The 3 first columns are used for increasing low concentrations (nM): A: 0.01; B: 0.02; C: 0.04; D: 0.05; E: 0.08; F: 0.1; G: 0.2; in triplicate. The 3 next columns (4 to 6) were used for the nonspecific binding. The 3 next columns (7 to 9) were used for higher concentrations: A: 0.3; B: 0.4; C: 0.5; D: 0.8; E: 1; F: 1.5 and G: 2. The last 3 columns, same concentrations, nonspecific binding. Nonspecific binding was done in the presence of 10 μM of cold melatonin. The H line was not used. R: 11 concentrations of each product. The concentrations of the products were from 10-14M to 10-4M (from column 1 to 11). Colum 12 is for unspecific binding. Two lines (A&B; C&D, etc.) were used per compounds. For DR in COS7 cell membranes, only 8 compounds were tested in that order from top to bottom: melatonin, 2-iodomelatonin, S 70254, 4P-P-DOT, S 20098/agomelatonin, S 22153, FLN68/ramelteon and Luzindole. For DR in CHO cell membranes in that order from top to bottom: melatonin, 2-iodomelatonin, 6-chlmromeltonin, Luzindole, 4PPDOT, S 20098/agomelatin, FLN68/ramelteon, D600, S20928, S21278, S22153, S70254, S73893, S75436, S27128, DIV880, SD6, SD1881, SD1882 and SD1918. If needed, more information can be obtained from the corresponding author upon request. Table A. Raw data for calculation of COS7 Xenopus Mel1c (n = 1 & 2) saturations. Table B. Raw data for calculation of COS7 Platypus Mel1c (n = 1) saturation. Table C. Raw data for calculation of COS7 Platypus (n = 2) & Xenopus (n = 3) Mel1c saturations. Table D. Raw data for calculation of COS7 Mel1c Platypus (n = 3) and naïve cells saturation. Table E. Raw data for calculation of CO7 Mel1c Chicken (n = 1 & 2) and naïve cells saturations. Table F. Raw data for calculation of CHO Mel1c Xenopus (n = 1) saturation. Table G. [file pone.0191904.s002.zip › Table H.pdf]

## Raw data Mel1C Plat N=2

| DPM | 1 | 2 | 3 | 4 | 5 | 6 | 7 | 8 | 9 | 10 | 11 | 12 |
|---|---|---|---|---|---|---|---|---|---|---|---|---|
| A | 2320 | 2619 | 2699 | 679 | 0 | 173 | 9612 | 8973 | 8615 | 329 | 321 | 278 |
| B | 4021 | 3814 | 3579 | 515 | 160 | 148 | 9410 | 9141 | 8615 | 392 | 544 | 329 |
| C | 5731 | 5896 | 6007 | 152 | 117 | 138 | 10025 | 9931 | 8795 | 479 | 424 | 529 |
| D | 6265 | 6117 | 6093 | 107 | 136 | 172 | 9611 | 9405 | 9356 | 592 | 740 | 684 |
| E | 7114 | 7321 | 7244 | 178 | 179 | 183 | 10146 | 9276 | 8456 | 821 | 770 | 723 |
| F | 7310 | 7467 | 8095 | 196 | 177 | 214 | 8609 | 8141 | 8088 | 1159 | 1017 | 887 |
| G | 8156 | 8438 | 8714 | 255 | 325 | 310 | 7814 | 7235 | 7074 | 1312 | 1479 | 1222 |
| H | 75 | 72 | 71 | 42 | 0 | 149 | 98 | 78 | 86 | 37 | 0 | 0 |

| CPM | 1 | 2 | 3 | 4 | 5 | 6 | 7 | 8 | 9 | 10 | 11 | 12 |
|---|---|---|---|---|---|---|---|---|---|---|---|---|
| A | 1268 | 1514 | 1539 | 37 | 42 | 60 | 5498 | 5223 | 5124 | 180 | 172 | 153 |
| B | 2147 | 2210 | 1957 | 42 | 43 | 64 | 5266 | 5383 | 5003 | 217 | 250 | 172 |
| C | 3101 | 3438 | 3417 | 60 | 45 | 59 | 5654 | 5795 | 5130 | 255 | 223 | 280 |
| D | 3254 | 3484 | 3421 | 45 | 56 | 80 | 5281 | 5431 | 5418 | 330 | 373 | 367 |
| E | 3754 | 4197 | 4104 | 81 | 86 | 89 | 5620 | 5348 | 4942 | 432 | 396 | 380 |
| F | 3909 | 4252 | 4493 | 88 | 84 | 104 | 4694 | 4660 | 4616 | 652 | 549 | 501 |
| G | 4402 | 4710 | 4857 | 133 | 181 | 151 | 4208 | 4033 | 4028 | 730 | 763 | 672 |
| H | 30 | 33 | 29 | 20 | 226 | 19 | 35 | 35 | 44 | 17 | 31 | 18 |

| Quer | 1 | 2 | 3 | 4 | 5 | 6 | 7 | 8 | 9 | 10 | 11 | 12 |
|---|---|---|---|---|---|---|---|---|---|---|---|---|
| A | 75.94 | 83.7 | 81.67 | 17.9 | 12.84 | 42.04 | 82.1 | 84.78 | 88.09 | 76.04 | 73.3 | 76.35 |
| B | 73.15 | 84.05 | 75.99 | 19.83 | 33.46 | 54.52 | 78.99 | 86.64 | 84.39 | 77.93 | 59.15 | 71.13 |
| C | 74.71 | 85.02 | 81.28 | 48.28 | 47.43 | 52.91 | 80.07 | 85.16 | 85.08 | 72.8 | 71.2 | 72.12 |
| D | 70.03 | 81.5 | 79.44 | 52.44 | 50.8 | 60.16 | 76.6 | 83.52 | 83.96 | 78.31 | 66.95 | 73.72 |
| E | 71.77 | 82.43 | 80.72 | 58 | 63.09 | 63.92 | 77.64 | 83.27 | 85.39 | 71.5 | 69.04 | 71.44 |
| F | 73.29 | 81.43 | 77.92 | 57.03 | 61.12 | 63.51 | 75.64 | 82.21 | 81.78 | 79.72 | 74.31 | 80.33 |
| G | 74.38 | 78.67 | 78.48 | 70.42 | 78.42 | 63.67 | 74.12 | 78.49 | 81.42 | 78.31 | 69.28 | 76.8 |
| H | 49.58 | 58.47 | 49.87 | 63.4 | 10.12 | 23.37 | 43.55 | 56.81 | 68.77 | 58.2 | 8.93 | 8.75 |

Totaux

| DPM | 1 | 2 | 3 | 4 | 5 | 6 | 7 | 8 | 9 | 10 | 11 | 12 |
|---|---|---|---|---|---|---|---|---|---|---|---|---|
| A | 762 | 1192 | 3843 | 500029 | 469694 | 472472 | 4127 | 1324 | 944 | 17192 | 19110 | 18493 |
| B | 1114 | 1875 | 5927 | 673864 | 650235 | 666542 | 6771 | 2087 | 1437 | 32832 | 36625 | 35347 |
| C | 1402 | 2536 | 8828 | 807089 | 784619 | 804582 | 9688 | 2883 | 2025 | 69349 | 75167 | 72418 |
| D | 1774 | 3288 | 12429 | 1362578 | 1383738 | 1398116 | 13110 | 3648 | 2549 | 80377 | 86544 | 83329 |
| E | 1989 | 4045 | 15862 | 1589608 | 1459987 | 1560695 | 16254 | 4409 | 2960 | 133853 | 135170 | 131670 |
| F | 2060 | 3933 | 19671 | 2521890 | 2394181 | 2452852 | 20040 | 4439 | 3356 | 168459 | 171465 | 162323 |
| G | 2005 | 3452 | 18725 | 3258790 | 3084283 | 3115413 | 19503 | 3720 | 3081 | 318981 | 333257 | 320049 |
| H | 1621 | 3232 | 9505 | 21009 | 24145 | 20399 | 9716 | 3612 | 2204 | 2599 | 2973 | 2300 |

| CPM | 1 | 2 | 3 | 4 | 5 | 6 | 7 | 8 | 9 | 10 | 11 | 12 |
|---|---|---|---|---|---|---|---|---|---|---|---|---|
| A | 457 | 769 | 2413 | 281465 | 269278 | 265088 | 2518 | 825 | 588 | 8793 | 8994 | 8881 |
| B | 674 | 1204 | 3697 | 382379 | 372576 | 381387 | 4112 | 1300 | 885 | 16775 | 17244 | 16773 |
| C | 837 | 1618 | 5466 | 450492 | 449504 | 443716 | 5858 | 1806 | 1241 | 35032 | 35711 | 34304 |

| | | | | | | | | | | | | |
|---|---|---|---|---|---|---|---|---|---|---|---|---|
| D | 1057 | 2083 | 7593 | 776640 | 787713 | 785975 | 7858 | 2239 | 1561 | 41046 | 40529 | 37989 |
| E | 1180 | 2527 | 9684 | 903650 | 838813 | 880167 | 9826 | 2725 | 1800 | 66989 | 64216 | 60786 |
| F | 1220 | 2457 | 12010 | 1477087 | 1447625 | 1444112 | 12120 | 2698 | 2057 | 83085 | 81444 | 75129 |
| G | 1184 | 2127 | 11438 | 1823568 | 1789423 | 1755160 | 11778 | 2253 | 1893 | 157959 | 167482 | 148241 |
| H | 953 | 2015 | 5801 | 12763 | 15111 | 12408 | 5874 | 2188 | 1359 | 1523 | 1629 | 1272 |

| Quer | 1 | 2 | 3 | 4 | 5 | 6 | 7 | 8 | 9 | 10 | 11 | 12 |
|---|---|---|---|---|---|---|---|---|---|---|---|---|
| A | 89.09 | 100.1 | 95.99 | 79.81 | 82.43 | 79.36 | 91.73 | 94.84 | 94.78 | 68.44 | 60.92 | 62.61 |
| B | 90.44 | 99.31 | 94.96 | 80.94 | 82.35 | 82.14 | 91.08 | 94.75 | 93.11 | 68.33 | 60.96 | 61.6 |
| C | 88.67 | 98.37 | 93.88 | 78.66 | 82.33 | 77.07 | 90.45 | 95.63 | 92.4 | 67.21 | 61.7 | 61.45 |
| D | 88.35 | 97.33 | 91.92 | 81.58 | 81.4 | 79.63 | 89.2 | 92.65 | 92.26 | 68.28 | 60.51 | 58.4 |
| E | 87.65 | 95.22 | 91.84 | 81.2 | 82.75 | 80.07 | 90.42 | 93.62 | 91.21 | 66.32 | 61.7 | 59.37 |
| F | 87.54 | 95.19 | 91.84 | 85.76 | 90.44 | 86.61 | 90.47 | 91.19 | 92.38 | 64.96 | 61.68 | 59.58 |
| G | 87.04 | 93.17 | 91.91 | 79 | 84.24 | 79.92 | 90.26 | 90.67 | 92.77 | 65.33 | 66.72 | 59.63 |
| H | 86.36 | 94.86 | 91.77 | 91.12 | 95.47 | 91.3 | 90.42 | 90.69 | 93.3 | 85.8 | 76.24 | 77.42 |
