# Supplementary material for: Characterization of the Mel1c melatoninergic receptor in platypus (Ornithorhynchus anatinus)
Source: PLoS One. 2018 Mar 12;13(3):e0191904. doi: 10.1371/journal.pone.0191904 (PMC5846726; doi:10.1371/journal.pone.0191904)
Supplement: S1 Data — They are counter listings, formatted to give the individual numbers used to calculate all the saturation curves and affinities reported in the present paper. The plates are all arranged the same ways: Saturation: The 3 first columns are used for increasing low concentrations (nM): A: 0.01; B: 0.02; C: 0.04; D: 0.05; E: 0.08; F: 0.1; G: 0.2; in triplicate. The 3 next columns (4 to 6) were used for the nonspecific binding. The 3 next columns (7 to 9) were used for higher concentrations: A: 0.3; B: 0.4; C: 0.5; D: 0.8; E: 1; F: 1.5 and G: 2. The last 3 columns, same concentrations, nonspecific binding. Nonspecific binding was done in the presence of 10 μM of cold melatonin. The H line was not used. R: 11 concentrations of each product. The concentrations of the products were from 10-14M to 10-4M (from column 1 to 11). Colum 12 is for unspecific binding. Two lines (A&B; C&D, etc.) were used per compounds. For DR in COS7 cell membranes, only 8 compounds were tested in that order from top to bottom: melatonin, 2-iodomelatonin, S 70254, 4P-P-DOT, S 20098/agomelatonin, S 22153, FLN68/ramelteon and Luzindole. For DR in CHO cell membranes in that order from top to bottom: melatonin, 2-iodomelatonin, 6-chlmromeltonin, Luzindole, 4PPDOT, S 20098/agomelatin, FLN68/ramelteon, D600, S20928, S21278, S22153, S70254, S73893, S75436, S27128, DIV880, SD6, SD1881, SD1882 and SD1918. If needed, more information can be obtained from the corresponding author upon request. Table A. Raw data for calculation of COS7 Xenopus Mel1c (n = 1 & 2) saturations. Table B. Raw data for calculation of COS7 Platypus Mel1c (n = 1) saturation. Table C. Raw data for calculation of COS7 Platypus (n = 2) & Xenopus (n = 3) Mel1c saturations. Table D. Raw data for calculation of COS7 Mel1c Platypus (n = 3) and naïve cells saturation. Table E. Raw data for calculation of CO7 Mel1c Chicken (n = 1 & 2) and naïve cells saturations. Table F. Raw data for calculation of CHO Mel1c Xenopus (n = 1) saturation. Table G. [file pone.0191904.s002.zip › Table I.pdf]

Plaque filtre - Sat- Xeno1 ok

DPM	1	2	3	4	5	6	7	8	9	10	11	12
A	1898	2742	2566	356	61	85	14402	12848	12589	293	237	278
B	4535	4347	4058	181	80	101	17104	15551	10405	393	267	291
C	5963	5477	4333	140	86	132	11542	9907	11889	593	368	307
D	6445	6186	3471	150	117	138	11598	12051	11182	587	661	419
E	7205	7669	5076	221	173	185	14403	12295	12586	694	482	418
F	9186	8157	6423	183	206	207	15189	15613	14278	1219	792	737
G	10866	11400	10913	288	222	256	16456	17870	14206	1313	972	751
H	65	86	68	118	70	53	134	156	93	68	61	78

CPM	1	2	3	4	5	6	7	8	9	10	11	12
A	944	1618	1540	56	30	45	8590	7704	7590	163	124	137
B	2728	2585	2448	64	35	57	10324	9240	6106	214	150	144
C	3548	3234	2589	70	50	74	6924	5845	7198	320	213	163
D	3848	3662	2047	69	60	71	6796	7217	6714	343	353	237
E	4224	4523	3023	114	82	101	8621	7212	7542	405	279	237
F	5447	4813	3837	95	118	112	9079	9314	8554	705	461	410
G	6455	6839	6543	144	109	141	9797	10530	8543	755	552	419
H	38	44	32	47	30	28	62	73	48	35	22	15

tSIS	1	2	3	4	5	6	7	8	9	10	11	12
A	55.66	81.35	86.54	26.66	53.17	61.27	84.71	86.21	87.86	65.85	60.03	54.51
B	87.15	83.79	88.07	38.51	45.96	66.49	88.25	83.59	79.27	63.3	66.93	55.19
C	83.99	81.58	85.25	56.5	71.27	67.15	86.3	81.26	89.24	62.72	70.78	60.95
D	85.02	82.4	81.15	49.6	58.58	58.88	75.52	85.84	86.56	74.61	61.63	67.81
E	76.82	81.08	84.29	58.67	51.73	63.89	85.73	79.06	86.02	72.8	70.32	67.78
F	82.92	81.29	85.14	59.33	69.11	62.61	85.34	84.75	85.95	70.2	71.55	65.58
G	83.51	86.33	86.15	56.03	54.7	64.71	84.16	80.82	87.03	69.39	68.02	65.94
H	73.91	58.87	51.62	42.26	45.32	60.71	49.62	50.94	58.28	58.37	38.58	27.78

Plaque filtre - Plaque1- Xeno1 ok

DPM	1	2	3	4	5	6	7	8	9	10	11	12
A	6368	6978	6906	7213	6782	4873	2031	425	156	94	90	120
B	6422	7277	7124	6567	7346	5587	2103	557	148	126	46	119
C	6450	7008	7357	7274	4708	1401	418	159	108	121	103	91
D	6350	7050	7449	7610	5123	1386	408	176	140	233	119	119
E	7134	7275	7221	7441	7255	7538	7828	6179	955	205	12804	6527
F	6624	6293	6787	7913	8098	7793	7496	6295	934	169	8861	6471
G	6357	6786	7110	6857	7781	6961	6898	5973	2661	514	219	6432
H	5631	6586	6518	6577	6803	6737	6425	5428	2263	389	226	6499

CPM	1	2	3	4	5	6	7	8	9	10	11	12
A	3762	4090	4119	4227	4050	2865	1200	249	90	43	26	36
B	3826	4303	4264	3850	4352	3304	1249	326	72	53	26	32
C	3850	4128	4388	4279	2760	824	240	76	48	58	33	40
D	3806	4153	4467	4509	3031	816	230	92	70	65	64	70

E	4226	4289	4259	4488	4285	4478	4717	3683	561	108	7674	3878
F	3973	3657	3978	4780	4776	4581	4507	3764	548	89	5330	3832
G	3740	4011	4230	4040	4640	4178	4081	3508	1560	301	128	3800
H	3316	3858	3749	3855	3997	3906	3758	3151	1284	226	111	3845

tSIS	1	2	3	4	5	6	7	8	9	10	11	12
A	81.7	78.75	84.72	77.69	85.05	80	81.71	77.68	70.45	49.88	33.89	34.33
B	84.35	82.01	85.68	76.91	82.63	82.04	83.37	78.74	53.51	44.43	65.18	31.74
C	84.9	80.66	84.72	80.12	77.38	79.81	68.97	52.44	47.7	52.93	36.3	46.53
D	86.08	80.73	86.23	82.69	82.22	80.36	66.75	59.81	56.45	32.85	61.89	76.37
E	82.63	80.97	81.14	87.97	81.61	83.48	87.69	84.47	79.28	60.66	86.07	83.59
F	86.32	71.35	77.67	88.51	81.07	79.89	86.95	85.43	77.68	60.23	87.13	82.46
G	80.19	81.87	83.99	80.76	84.65	86.42	82.18	79.53	78.89	75.47	71.28	81.74
H	80.6	78.43	69.51	75.84	79.76	70.65	74.03	70.97	67.81	72.06	55	82.22

Plaque filtre - Plaque2- Xeno1 ok

DPM	1	2	3	4	5	6	7	8	9	10	11	12
A	6412	7273	7460	7349	7379	4284	1239	269	68	140	70	6977
B	7232	7684	7191	7541	7219	4496	1248	301	99	142	38	93
C	6750	6626	8127	7819	8052	8180	7723	5689	1566	276	87	107
D	6776	7541	7677	8450	8332	8453	7726	5142	1695	276	121	74
E	6616	7416	7768	7785	4976	1384	332	111	117	108	134	6297
F	7092	7110	7602	7898	5054	1291	359	180	140	85	185	6562
G	6897	6662	8011	7725	6945	7789	7608	7589	6177	2799	483	6480
H	6021	7390	6844	7301	7225	6714	6830	7090	5547	2375	450	6349

CPM	1	2	3	4	5	6	7	8	9	10	11	12
A	3320	4261	4403	4373	4381	2511	729	146	34	45	33	25
B	4237	4505	4212	4453	4284	2642	731	176	52	49	21	37
C	3961	3859	4824	4595	4750	4823	4617	3368	922	161	41	36
D	3967	4418	4594	5003	4911	4971	4542	2994	993	161	52	43
E	3904	4345	4576	4601	2952	811	194	66	58	51	79	3692
F	4205	4168	4466	4641	2867	745	207	100	66	44	103	3846
G	4043	3904	4747	4557	3827	4611	4528	4448	3636	1650	278	3842
H	3529	4332	4011	4306	4238	3934	3965	3991	3252	1397	261	3723

tSIS	1	2	3	4	5	6	7	8	9	10	11	12
A	58.94	75.29	81.41	84.03	83.33	78.71	80.09	63.21	56.55	36.21	51.8	22.06
B	78.01	76.97	78.39	81.58	83.15	79.87	78.74	73.41	61	37.76	64.81	42.58
C	79.2	72.06	83.23	79.75	81.24	81.05	85.34	82.47	80.71	74.89	50.37	37.71
D	74.79	75.34	85.65	82.44	80.89	80.04	79.94	71.97	78.54	72.34	44.9	69.99
E	81.34	78.3	80.67	81.86	83.12	78.17	73.54	81.49	55.06	51.12	78.41	76.67
F	82.91	77.19	79.68	79.76	67.82	69.93	69.79	65.26	51.54	59.57	66.02	78.74
G	77.08	75.54	82.72	81.21	64.61	82.41	84.09	78.77	80.35	80.89	69.47	82.85
H	77.6	77.39	78.68	81.09	79.07	77.91	71.02	66.93	77.32	80.01	71.53	78.87

Plaque filtre - Sat- Xeno2

DPM	1	2	3	4	5	6	7	8	9	10	11	12
A	954	971	965	162	0	54	4566	4426	4323	180	183	132
B	1501	1514	1079	497	0	123	4636	4650	4649	288	178	216
C	2054	1594	1308	0	119	98	4857	4932	4551	374	244	252
D	2267	1847	1338	130	60	118	5207	5076	4747	394	404	367
E	2639	2448	1834	146	162	110	5325	4852	4510	730	385	338
F	3218	2893	2335	139	139	115	5541	4831	4735	637	488	545
G	3510	3442	3550	231	181	180	5472	5581	5177	878	787	619
H	50	33	42	0	76	31	58	69	60	61	61	0

CPM	1	2	3	4	5	6	7	8	9	10	11	12
A	559	563	566	38	17	25	2699	2604	2537	100	96	68
B	879	886	633	37	27	34	2729	2738	2743	164	105	120
C	1203	935	766	40	39	46	2865	2906	2681	198	129	120
D	1349	1083	783	59	26	40	3053	2989	2783	225	213	179
E	1551	1433	1076	74	57	61	3127	2844	2644	389	216	169
F	1897	1696	1369	72	68	63	3256	2833	2780	363	277	294
G	2057	2017	2086	109	76	99	3215	3271	3039	512	417	324
H	24	17	18	28	20	16	29	31	26	24	29	13

tSIS	1	2	3	4	5	6	7	8	9	10	11	12
A	75.89	70.54	77.79	29.2	10.85	50.3	81.85	80.31	79.24	66.02	60.17	58.02
B	74.79	74.27	79.11	24.27	14.06	32.28	80.47	80.54	81.26	68.55	76.26	65.27
C	78.62	76.98	77.99	20.37	36.69	51.65	81.11	80.82	80.68	60.95	60.83	52.14
D	83.99	75.78	74.45	48.48	44.93	37.22	78.91	80.51	77.02	68.39	60.54	53.68
E	79.82	75	79.13	56.95	38.26	65.59	79.55	77.77	77.23	61.39	66.54	56.18
F	80.87	76.24	77.43	58.79	54.24	64.76	79.74	79.04	79.43	68.48	68.09	62.37
G	77.65	76.01	79.64	51.58	44.42	63.74	79.67	76.25	79.44	72.63	60.92	59.96
H	53.06	55.09	45.75	8.63	31.05	55.54	56.32	47.6	44.81	42.64	53.49	11.78

Plaque filtre - Plaque1- Xeno2

DPM	1	2	3	4	5	6	7	8	9	10	11	12
A	2572	2645	3169	2454	2799	1952	769	210	63	124	55	99
B	2456	2438	2556	2908	2514	2008	741	245	110	141	57	78
C	2522	2755	2608	2676	1650	604	232	122	93	85	69	63
D	2706	2712	2405	2840	1850	562	184	121	95	115	131	138
E	2603	2646	2725	2988	2772	2998	3104	2386	378	218	12029	2386
F	2487	2676	2748	2726	2894	2792	2767	2409	344	136	10907	2705
G	2356	2546	2748	2551	3088	2877	2813	2291	1066	254	166	2479
H	1971	2417	2375	2515	2689	2709	2798	2245	890	195	129	2502

CPM	1	2	3	4	5	6	7	8	9	10	11	12
A	1507	1547	1858	1438	1640	1144	448	109	31	36	25	25
B	1435	1426	1498	1706	1473	1177	432	134	55	51	28	41
C	1477	1611	1528	1568	964	339	126	61	40	45	40	31
D	1585	1589	1411	1666	1085	322	104	62	42	55	53	52
E	1526	1549	1601	1754	1625	1757	1820	1398	207	126	7093	1377

F	1459	1568	1610	1597	1701	1636	1622	1410	178	69	6480	1561
G	1381	1491	1610	1478	1812	1689	1651	1343	621	147	85	1448
H	1155	1410	1392	1482	1576	1520	1639	1314	516	111	57	1384

tSIS	1	2	3	4	5	6	7	8	9	10	11	12
A	75.7	74.02	76.96	77.32	78.7	76.48	71.95	59.13	55.54	33.59	47.98	30.07
B	73.59	74.1	76.69	79.09	78.32	76.18	72.42	63.59	56.03	39.32	54.72	60.17
C	74.81	73.94	75.61	77.85	73.21	66.58	63.16	55.93	45.09	60.79	70.17	55.34
D	78.52	75.96	79.06	79.12	79.11	69.1	66.76	58.82	47.17	53	42.95	40.48
E	77.3	74.7	79.8	79.37	76.13	77.42	76.93	74.93	64.27	69.98	81.05	69.99
F	79.07	75.79	75.57	75.59	79.94	78.03	77.88	74.42	58.76	57.21	83.54	69.91
G	75.86	75.04	76.04	70.58	79.27	79.41	79.38	78.81	72.12	70.26	58.22	73.18
H	75.29	72.79	77.8	80.92	75.73	66.53	74.98	74.45	70.67	68.39	46.85	65.04

Plaque filtre - Plaque2- Xeno2

DPM	1	2	3	4	5	6	7	8	9	10	11	12
A	2172	2512	2410	2394	2276	1362	434	169	74	100	133	124
B	2202	2401	2573	2505	2412	1586	459	170	160	91	82	95
C	2070	2355	2423	2783	2735	2653	2359	1788	603	179	138	176
D	2261	2606	2539	2768	2699	2705	2645	1867	603	181	116	157
E	1968	2238	2210	2397	1631	474	220	148	108	151	135	2327
F	1981	2271	2370	2364	1654	420	153	97	155	127	160	2272
G	2168	2284	2283	2551	2652	2588	2424	2484	2161	876	244	2165
H	1963	2115	1923	2235	2449	2432	2368	2356	1900	762	171	2095

CPM	1	2	3	4	5	6	7	8	9	10	11	12
A	1223	1454	1413	1404	1334	793	250	84	33	44	34	35
B	1290	1394	1510	1472	1413	929	267	96	39	45	32	36
C	1209	1371	1416	1629	1604	1547	1383	1045	346	95	47	39
D	1325	1507	1488	1620	1582	1585	1550	1073	345	91	47	50
E	1153	1309	1292	1405	950	272	107	66	47	60	75	1360
F	1157	1323	1389	1385	969	232	83	49	47	59	80	1324
G	1265	1335	1334	1492	1554	1513	1421	1453	1254	513	119	1261
H	1133	1173	1125	1261	1411	1335	1343	1359	1097	444	84	1209

tSIS	1	2	3	4	5	6	7	8	9	10	11	12
A	66.98	70.38	76.32	77.02	75.88	71.79	69.7	55.49	46.2	46.78	30.27	32.52
B	76.03	71.05	79.28	79.83	78.01	74.71	71.1	67.01	29.53	54.85	42.05	41.27
C	73.12	71.77	73.45	74.8	79.06	72.44	76.14	73.46	69.36	61	37.71	28.63
D	77.38	70.23	76.25	74.49	76.56	75.52	75.79	69.35	68.96	56.8	42.87	36.08
E	74.91	74.03	73.6	76.01	72.08	68.89	53.83	47.21	46.61	42.55	64.88	73.71
F	73.32	72.11	77.39	75.33	75.55	64.85	62.72	56.54	34.97	49.6	55.8	72.08
G	72.82	73.45	73.54	73.84	76.28	73.48	76.46	74.23	70.83	74.48	53.79	72.02
H	70.03	65.29	74.07	67.16	69.79	64.2	67.76	69.9	69.95	72.79	55.02	69.96

Totaux

DPM	1	2	3	4	5	6	7	8	9	10	11	12

A	0	0	0	0	0	0	0	0	0	68399	65497	69167
B	0	0	0	0	0	0	0	40	0	77859	73237	67381
C	0	0	0	0	0	21	0	0	0	73109	78651	73635
D	0	0	6	0	33	0	0	10	0	73390	75303	77533
E	0	0	0	0	139	0	0	0	0	72724	72655	70670
F	0	0	0	0	0	0	0	0	0	69921	78031	78685
G	12	0	0	0	0	0	0	0	0	75244	69461	68094
H	0	0	0	0	0	0	0	0	0	685	701	636

CPM	1	2	3	4	5	6	7	8	9	10	11	12
A	5	8	7	22	9	4	3	4	10	40428	38395	40547
B	5	4	7	22	8	7	5	4	7	45772	42837	39485
C	4	5	6	23	9	8	4	4	11	43143	45983	43087
D	6	3	4	25	10	5	5	7	10	43189	44112	45356
E	6	6	7	25	8	5	6	4	7	42821	42555	41368
F	4	2	3	19	10	4	5	7	9	41147	45695	46047
G	3	5	4	22	8	6	7	4	9	44326	40714	39907
H	4	8	4	20	8	3	4	4	2	420	431	380

tSIS	1	2	3	4	5	6	7	8	9	10	11	12
A	4.08	10.6	7.24	7.71	6.38	6.58	6.29	6.26	7.07	81.88	76.46	76.95
B	14.88	5.27	13	6.78	6.43	9.1	8.42	25.06	7.22	79.94	74.02	75.64
C	4.17	10.21	9.28	7.66	7.36	39.14	8.73	8.77	9.31	81.33	73.75	74.28
D	8.06	5.27	143.85	6.78	35.56	8.51	10.72	129.47	9.4	80.33	75.22	74.1
E	13.54	5.32	8.25	7.78	23.74	4.28	7.15	8.77	7.27	80.54	75.07	74.56
F	10.03	4.34	3.2	6.8	6.18	7.63	5.38	8.39	8.21	80.32	74.87	74.35
G	34.39	13.01	8.15	6.74	7.61	21.88	8.63	12.71	9.25	80.71	77.45	75.8
H	5.53	16.45	-1	7.79	5.36	12.19	7.61	8.13	-1	93.44	94	85.3
