# Supplementary material for: Characterization of the Mel1c melatoninergic receptor in platypus (Ornithorhynchus anatinus)
Source: PLoS One. 2018 Mar 12;13(3):e0191904. doi: 10.1371/journal.pone.0191904 (PMC5846726; doi:10.1371/journal.pone.0191904)
Supplement: S1 Data — They are counter listings, formatted to give the individual numbers used to calculate all the saturation curves and affinities reported in the present paper. The plates are all arranged the same ways: Saturation: The 3 first columns are used for increasing low concentrations (nM): A: 0.01; B: 0.02; C: 0.04; D: 0.05; E: 0.08; F: 0.1; G: 0.2; in triplicate. The 3 next columns (4 to 6) were used for the nonspecific binding. The 3 next columns (7 to 9) were used for higher concentrations: A: 0.3; B: 0.4; C: 0.5; D: 0.8; E: 1; F: 1.5 and G: 2. The last 3 columns, same concentrations, nonspecific binding. Nonspecific binding was done in the presence of 10 μM of cold melatonin. The H line was not used. R: 11 concentrations of each product. The concentrations of the products were from 10-14M to 10-4M (from column 1 to 11). Colum 12 is for unspecific binding. Two lines (A&B; C&D, etc.) were used per compounds. For DR in COS7 cell membranes, only 8 compounds were tested in that order from top to bottom: melatonin, 2-iodomelatonin, S 70254, 4P-P-DOT, S 20098/agomelatonin, S 22153, FLN68/ramelteon and Luzindole. For DR in CHO cell membranes in that order from top to bottom: melatonin, 2-iodomelatonin, 6-chlmromeltonin, Luzindole, 4PPDOT, S 20098/agomelatin, FLN68/ramelteon, D600, S20928, S21278, S22153, S70254, S73893, S75436, S27128, DIV880, SD6, SD1881, SD1882 and SD1918. If needed, more information can be obtained from the corresponding author upon request. Table A. Raw data for calculation of COS7 Xenopus Mel1c (n = 1 & 2) saturations. Table B. Raw data for calculation of COS7 Platypus Mel1c (n = 1) saturation. Table C. Raw data for calculation of COS7 Platypus (n = 2) & Xenopus (n = 3) Mel1c saturations. Table D. Raw data for calculation of COS7 Mel1c Platypus (n = 3) and naïve cells saturation. Table E. Raw data for calculation of CO7 Mel1c Chicken (n = 1 & 2) and naïve cells saturations. Table F. Raw data for calculation of CHO Mel1c Xenopus (n = 1) saturation. Table G. [file pone.0191904.s002.zip › Table J.pdf]

DR1 platypus

	1	2	3	4	5	6	7	8	9	10
A	7016	7159	8119	6965	7614	6059	3256	823	134	96
B	6821	6936	6739	6110	6806	6206	3124	733	149	94
C	7216	6604	6442	5998	4509	1350	269	115	66	153
D	6994	6119	5972	5996	4352	1238	263	135	85	147
E	6485	6074	6208	6628	6870	7140	6485	6135	3040	303
F	7136	6379	7501	6801	7336	7243	6933	6784	2818	249
G	7295	7449	8004	8008	8191	7542	6822	6551	3164	627
H	7095	7588	7836	7771	7752	7588	7504	6194	3311	570

	1	2	3	4	5	6	7	8	9	10
A	3685	4192	4759	4083	4462	3544	1907	479	75	39
B	3996	4039	3951	3581	4015	3638	1832	430	75	47
C	4229	3861	3799	3541	2648	791	135	58	34	46
D	4100	3562	3506	3556	2550	726	150	57	44	51
E	3802	3554	3639	3893	4025	4242	3802	3596	1767	169
F	4183	3738	4423	3995	4320	4258	4064	3984	1652	138
G	4276	4363	4725	4747	4393	4420	3999	3851	1857	367
H	4063	4426	4593	4555	4547	4445	4395	3631	1940	333

	1	2	3	4	5	6	7	8	9	10
A	60.13	74.77	77.13	77.28	78.71	73.94	75.30	71.46	65.92	42.57
B	78.29	72.01	76.60	75.79	81.25	77.47	77.08	76.10	57.41	56.43
C	75.68	73.79	81.06	81.48	79.48	77.88	56.23	57.10	58.01	34.66
D	76.43	71.93	79.35	82.96	78.13	76.21	68.07	44.95	59.98	37.95
E	77.28	74.17	76.40	79.56	78.56	83.57	76.86	76.20	71.42	66.29
F	77.47	75.61	81.02	79.60	80.63	79.90	77.42	79.52	77.37	65.50
G	76.52	75.05	81.42	82.86	61.98	75.65	76.56	79.96	79.26	74.60
H	68.97	72.62	77.22	77.45	79.03	78.34	74.97	77.01	78.61	73.64

DR2 platypus

	1	2	3	4	5	6	7	8	9	10
A	8398	7451	7167	7129	6191	3187	754	170	69	97
B	6737	6991	6741	6932	6412	3151	824	153	89	111
C	6937	7037	6981	7491	7564	7296	6614	3407	766	193
D	6694	7667	7055	8079	7210	7423	6445	3261	709	182
E	7165	7443	7423	7302	5457	1711	356	157	95	107
F	7090	7212	7708	7322	5690	1532	323	142	122	93
G	7352	7442	7558	7902	9066	7991	7532	7657	6162	2478
H	7443	7436	7144	7409	7519	7962	7389	7205	6132	2245

	1	2	3	4	5	6	7	8	9	10
A	4337	4347	4201	4177	3629	1841	439	85	32	35
B	3938	4075	3950	4062	3758	1831	478	88	39	46
C	4053	4097	4090	4386	4434	4269	3875	1997	426	100
D	3901	4488	4129	4732	4225	4351	3777	1912	408	100

E	4199	4357	4341	4280	3199	1000	197	66	41	44
F	4151	4174	4519	4292	3307	877	170	70	53	42
G	4304	4320	4430	4649	4866	4682	4432	4488	3612	1453
H	4352	4314	4177	4335	4383	4664	4316	4209	3588	1313

	1	2	3	4	5	6	7	8	9	10
A	58.73	72.71	76.65	75.58	77.16	70.06	71.96	56.69	50.21	39.46
B	73.68	72.39	75.52	75.59	75.86	71.38	70.74	70.04	46.82	44.00
C	73.31	71.95	78.11	74.71	76.94	74.36	75.51	76.96	65.63	59.20
D	72.24	74.51	74.46	75.04	78.08	77.55	77.69	76.64	69.76	64.34
E	75.98	74.53	73.89	77.44	76.88	73.57	65.12	44.58	45.53	43.40
F	74.76	70.32	76.78	76.59	71.38	68.85	60.50	54.49	46.17	48.54
G	74.64	70.96	75.89	80.24	62.06	77.97	80.30	77.68	77.65	76.24
H	73.88	70.83	73.78	74.34	72.39	75.33	73.34	73.35	74.29	74.31

Totaux

	1	2	3	4	5	6	7	8	9	10
A	0	0	0	0	0	0	0	0	0	58873
B	0	0	0	0	0	0	0	0	0	58659
C	0	0	0	0	0	0	0	0	0	58928
D	0	0	0	0	37	0	0	0	0	60530
E	0	0	0	0	0	0	0	0	0	55048
F	0	0	0	0	0	0	0	0	0	61876
G	0	0	5	0	96	0	0	0	0	51064
H	0	0	0	0	0	0	0	0	0	66689

	1	2	3	4	5	6	7	8	9	10
A	5	7	6	30	7	6	5	3	6	34654
B	6	5	4	26	12	4	5	5	7	34507
C	6	7	5	30	19	4	4	6	14	34672
D	6	6	5	17	11	4	6	6	9	35510
E	6	8	4	27	8	7	5	6	11	32510
F	4	12	3	30	7	6	6	5	15	36410
G	2	5	3	28	8	7	6	6	15	30170
H	4	3	5	23	10	6	6	5	3	39138

	1	2	3	4	5	6	7	8	9	10
A	6.21	12.92	10.57	8.76	7.79	6.93	6.50	-1.00	12.92	80.42
B	11.83	6.42	7.14	6.79	8.28	10.53	6.39	8.49	14.85	80.19
C	5.24	10.45	7.27	8.81	10.38	9.91	4.26	18.31	8.31	80.27
D	6.31	7.15	5.04	6.84	33.13	4.25	6.45	5.10	7.10	79.11
E	6.04	12.72	6.98	6.74	6.42	8.57	7.31	11.20	7.11	81.60
F	8.25	10.97	8.84	7.83	4.18	7.29	7.62	9.53	9.47	80.30
G	11.68	4.27	53.37	9.86	24.52	12.40	8.42	13.86	9.44	81.74
H	13.99	11.96	6.11	8.87	16.33	-1.00	7.96	6.31	8.83	79.27

11	12
1074	154
49	141
67	124
129	183
14014	5674
13718	5877
229	6070
208	6219

11	12
24	71
23	59
24	68
59	82
8226	3321
8165	3430
116	3502
110	3645

11	12
22.63	49.83
51.54	44.52
38.57	63.90
49.05	47.70
79.34	74.54
84.09	72.91
57.33	69.92
60.60	75.99

11	12
74	123
88	158
92	149
81	148
222	7251
235	6640
388	6802
418	7297

11	12
39	64
30	73
24	76
43	80

117	4237
133	3890
225	3982
233	4274

11	12
59.80	58.79
37.43	50.22
30.62	57.37
60.96	62.14
60.62	73.48
67.33	75.26
70.66	74.69
65.75	75.17

11	12
60006	59233
58621	59152
58043	58241
62422	57123
59257	61062
59914	58191
51943	51932
67749	67111

11	12
35154	34703
34336	34663
34022	34131
36589	33464
34712	35795
35088	34112
30442	30436
39715	39270

11	12
75.32	75.37
75.09	75.65
76.14	75.74
76.20	75.25
75.20	76.54
74.95	76.59
78.70	77.73
77.08	74.28
