# Supplementary material for: Characterization of the Mel1c melatoninergic receptor in platypus (Ornithorhynchus anatinus)
Source: PLoS One. 2018 Mar 12;13(3):e0191904. doi: 10.1371/journal.pone.0191904 (PMC5846726; doi:10.1371/journal.pone.0191904)
Supplement: S1 Data — They are counter listings, formatted to give the individual numbers used to calculate all the saturation curves and affinities reported in the present paper. The plates are all arranged the same ways: Saturation: The 3 first columns are used for increasing low concentrations (nM): A: 0.01; B: 0.02; C: 0.04; D: 0.05; E: 0.08; F: 0.1; G: 0.2; in triplicate. The 3 next columns (4 to 6) were used for the nonspecific binding. The 3 next columns (7 to 9) were used for higher concentrations: A: 0.3; B: 0.4; C: 0.5; D: 0.8; E: 1; F: 1.5 and G: 2. The last 3 columns, same concentrations, nonspecific binding. Nonspecific binding was done in the presence of 10 μM of cold melatonin. The H line was not used. R: 11 concentrations of each product. The concentrations of the products were from 10-14M to 10-4M (from column 1 to 11). Colum 12 is for unspecific binding. Two lines (A&B; C&D, etc.) were used per compounds. For DR in COS7 cell membranes, only 8 compounds were tested in that order from top to bottom: melatonin, 2-iodomelatonin, S 70254, 4P-P-DOT, S 20098/agomelatonin, S 22153, FLN68/ramelteon and Luzindole. For DR in CHO cell membranes in that order from top to bottom: melatonin, 2-iodomelatonin, 6-chlmromeltonin, Luzindole, 4PPDOT, S 20098/agomelatin, FLN68/ramelteon, D600, S20928, S21278, S22153, S70254, S73893, S75436, S27128, DIV880, SD6, SD1881, SD1882 and SD1918. If needed, more information can be obtained from the corresponding author upon request. Table A. Raw data for calculation of COS7 Xenopus Mel1c (n = 1 & 2) saturations. Table B. Raw data for calculation of COS7 Platypus Mel1c (n = 1) saturation. Table C. Raw data for calculation of COS7 Platypus (n = 2) & Xenopus (n = 3) Mel1c saturations. Table D. Raw data for calculation of COS7 Mel1c Platypus (n = 3) and naïve cells saturation. Table E. Raw data for calculation of CO7 Mel1c Chicken (n = 1 & 2) and naïve cells saturations. Table F. Raw data for calculation of CHO Mel1c Xenopus (n = 1) saturation. Table G. [file pone.0191904.s002.zip › Table K.pdf]

Totaux 11DR

DPM	1	2	3	4	5	6	7	8	9	10	11	12
A	0	0	0	0	63628	63919	63291	535	560	66878	67438	67986
B	0	0	0	0	63928	63739	62811	738	758	65737	66926	66707
C	0	0	0	0	64372	63629	63736	797	830	66663	67355	66925
D	0	0	0	0	65061	63769	63412	791	879	67016	67540	67091
E	0	0	0	0	63842	63574	62985	814	857	66772	67359	66961
F	0	0	0	0	63471	62679	63531	908	764	66487	67315	67999
G	0	0	11	0	64046	63155	63765	798	746	66630	67301	67622
H	0	0	0	0	63255	61953	62199	640	573	66323	66205	66446

CPM	1	2	3	4	5	6	7	8	9	10	11	12
A	5	3	4	37	37203	37463	37070	326	344	39205	39346	39592
B	5	8	4	42	37385	37365	36779	452	463	38537	39053	39039
C	4	5	8	37	37695	37283	37326	488	511	39066	39379	39083
D	4	7	8	31	38140	37367	37077	487	539	39278	39541	39262
E	3	7	5	29	37390	37244	36820	497	526	39132	39386	39183
F	6	4	4	31	37173	36727	37239	555	470	38967	39364	39808
G	6	7	6	30	37534	37022	37351	491	457	39059	39278	39578
H	5	9	4	29	37042	36318	36457	392	351	38902	38712	38886

tSIS	1	2	3	4	5	6	7	8	9	10	11	12
A	6.02	15.17	8.24	8.61	73.8	77.66	75.05	91.58	93.52	76.6	72.75	72.03
B	6.11	8.8	2.48	10.26	73.9	76.69	74.81	93.62	92.5	76.69	72.82	74.38
C	4.17	5.98	11.21	8.89	74.85	75.52	74.94	93.12	94.95	77.89	73.76	73.17
D	3.11	7.45	7.99	9.07	77.05	75.6	73.8	94.84	93.33	77.62	74.66	74.34
E	6.12	12.81	4.17	7.88	74.98	75.3	73.69	92.38	94.13	77.77	73.82	74.3
F	15.48	4.17	7.37	7.56	75	75.51	76.18	92.36	94.09	77.68	73.87	74.63
G	5.14	8.55	60.53	6.87	75.79	76.49	75.16	94.72	92.86	76.56	72.89	74.46
H	8.35	7.5	7.27	7.9	74.87	76.69	76.09	92.94	93.52	79.05	73.84	74.39

11DR1-Chicken1

DPM	1	2	3	4	5	6	7	8	9	10	11	12
A	23549	22771	22705	22452	21709	17433	8619	2063	297	106	69	85
B	22963	22512	21836	22301	23057	17894	8491	2007	358	113	62	116
C	23469	22678	23853	23181	19718	7963	1734	407	163	140	84	127
D	23724	23155	23364	21857	18973	8267	1850	494	223	130	127	202
E	23874	23364	22156	23845	23119	20855	22931	18145	2398	239	332	20915
F	23185	23526	22016	23956	23260	21923	23085	18253	2173	250	573	20424
G	24095	22590	24177	23137	24524	22174	22027	19556	7778	1288	567	22048
H	21940	22249	21924	20493	21542	21478	20675	17455	7358	1146	452	20485

CPM	1	2	3	4	5	6	7	8	9	10	11	12
A	13688	13348	13303	13165	12780	10214	5052	1209	173	47	32	35
B	13456	13197	12798	13139	13527	10560	4976	1177	198	58	30	45
C	13758	13300	13977	13699	11555	4668	1019	232	87	58	42	49
D	13905	13574	13698	12751	11122	4844	1084	280	123	56	61	107

E	13995	13687	12982	14033	13547	12143	13452	10637	1406	134	177	12253
F	13588	13789	12625	14094	13621	12842	13523	10698	1274	140	332	11945
G	14122	13212	14185	13559	14429	13095	12931	11494	4578	757	299	12923
H	12812	13043	12848	11984	12609	12085	12088	10144	4296	670	240	12009

tSIS	1	2	3	4	5	6	7	8	9	10	11	12
A	71.39	76.5	78.19	78.92	80.47	78.17	77.13	77.73	71.63	46.81	50.16	44.1
B	77.92	76.4	77.58	80.76	79.12	81.36	75.74	77.45	65.04	58.26	53.93	41.26
C	76.75	78.97	78.06	81.82	77.87	76.38	79.85	68.09	61.85	43.6	55.36	41.19
D	77.49	76.83	78.85	72.7	76.96	78.14	77.31	67.76	64.55	45.49	53.16	60.91
E	77.28	75.25	78.04	80.35	78	71.99	79.09	76.42	77.48	65.92	61.19	75.25
F	75.84	77.63	69.13	80.23	74.9	75.15	78.43	77.59	76.78	66.07	70.64	73.96
G	75.95	73.95	79.15	78.69	80.24	81.6	79.39	79.84	80.42	79.74	60.45	77.42
H	73.17	76.43	75.67	73.89	74.52	66.86	73.76	71.34	73.09	73.76	61.09	76.91

11DR1-Chicken1

DPM	1	2	3	4	5	6	7	8	9	10	11	12
A	21956	20417	18956	19949	19508	13093	5024	888	159	111	51	104
B	18464	17941	18817	19703	18867	13718	5560	999	243	150	70	155
C	19304	17897	18741	20368	20365	20027	18737	15302	5832	826	138	101
D	19487	16706	16796	19648	20727	19486	18872	14817	5565	957	217	168
E	17656	18324	16736	16403	15300	7796	1358	308	184	103	144	19373
F	19213	18345	17485	18051	17231	7318	1371	321	250	151	168	18116
G	19538	17586	17967	18935	22004	18865	18177	18470	13211	4550	788	19315
H	19437	17372	18441	17712	17994	18559	18894	18088	13744	5003	960	19191

CPM	1	2	3	4	5	6	7	8	9	10	11	12
A	11384	11941	10309	11743	11472	7670	2945	520	86	39	28	40
B	10805	10508	11029	11595	11053	8112	3258	586	122	59	32	39
C	11295	10489	10988	12012	11938	11744	10961	8971	3428	487	75	45
D	11406	9771	9820	11518	12159	11422	11060	8642	3262	560	99	79
E	10305	10741	9811	9614	8934	4570	795	166	90	55	68	11355
F	11248	10739	10203	10580	10101	4294	800	171	130	81	83	10618
G	11439	10284	10464	11109	11307	11060	10623	10821	7742	2673	441	11326
H	11376	10145	10810	10383	10539	10878	11058	10585	8051	2932	472	11172

tSIS	1	2	3	4	5	6	7	8	9	10	11	12
A	59.06	73.99	63.3	80.41	80.07	78.53	77.3	75.04	63.12	38.3	67	40.95
B	74.34	75.08	77.52	80.31	78.26	82.02	75.44	76.21	56.59	41.74	48.69	30.19
C	74.26	75.84	78.89	81.11	76.63	78.94	74.13	77	79.92	80.85	62.59	47.17
D	74.49	73.98	73.76	76.45	79.08	77.25	75.78	72.62	76.62	78.41	48.58	51.04
E	72.93	77.28	76.37	77.59	73.12	78.81	74.6	62.23	53.82	61.55	51.08	76.07
F	74.61	74.58	72.8	77.65	76.62	79.24	72.61	61.31	58.98	61.87	55.02	77.61
G	74.71	73.86	72.05	79.15	58.33	78.87	73.53	75.37	77.91	79.64	66.21	78.93
H	74.46	73.18	76.3	76.36	75.02	77.5	74.42	74.31	75.28	77.56	54.53	71.9

11DR1-Chicken2

DPM	1	2	3	4	5	6	7	8	9	10	11	12
A	18684	20268	18408	18824	19560	14232	6728	1609	233	115	59	0
B	19245	18461	18317	18984	19320	14448	7280	1571	274	121	65	126
C	18611	18206	18768	19678	15518	7079	1412	346	192	87	128	66
D	18386	18557	20657	20299	15565	6513	1521	354	194	98	95	186
E	20222	20070	19590	20633	20955	21096	19164	15383	1815	199	497	18832
F	19152	18045	19223	19080	20331	19338	19634	15550	1721	249	1763	17883
G	19732	20345	22094	20168	23334	19561	18782	16324	6490	1194	516	17513
H	17941	18627	17121	18030	18483	18889	19509	16304	6460	1051	453	17448

CPM	1	2	3	4	5	6	7	8	9	10	11	12
A	10797	11836	10777	11030	11463	8343	3939	943	125	45	27	29
B	11239	10821	10718	11129	11326	8469	4268	920	154	60	32	35
C	10894	10581	10991	11571	9093	4148	825	187	100	48	34	33
D	10763	10858	12105	11945	9111	3817	887	202	105	50	51	75
E	11813	11759	11479	12095	12198	12342	11231	8993	1056	104	288	11022
F	11182	10577	11261	11183	11913	11335	11489	9097	1005	136	1032	10475
G	11532	11925	12952	11835	13675	11468	10998	9551	3804	700	289	10231
H	10481	10894	9978	10570	10799	11073	11048	8994	3664	610	261	10169

tSIS	1	2	3	4	5	6	7	8	9	10	11	12
A	70.11	73.14	74.7	75.56	75.72	76.69	74.68	76.06	62.27	41.7	48.07	16.58
B	73.17	76.3	74.3	76.92	76.92	77.19	76.47	74.79	66.89	55.41	55.81	32.49
C	74.54	71.37	74.91	80.02	75.55	77.94	73.27	62.76	58.92	63.65	31.46	56.98
D	74.59	74.23	77.98	80.31	74.5	77.72	72.68	68.47	62.45	58.8	61.93	42.89
E	73.32	75.41	75.53	76.4	71.89	74.16	75.7	73.71	71.72	60.04	70.38	74.44
F	73.05	76.15	75.21	77.58	75.51	77.56	74.33	74.15	72.92	63.54	74.29	75.12
G	73.57	76.09	76.65	79.21	75.81	78.83	74.82	74.22	75.88	77.15	66.6	73.36
H	73.34	73.95	72.3	76.86	73.41	76.97	67.61	64.73	67.79	71	69.61	72.34

11DR2-Chicken2

	1	2	3	4	5	6	7	8	9	10	11	12
A	20321	19444	19450	18933	18474	12200	4391	799	130	75	69	62
B	19196	17885	17976	18963	18554	13968	5665	956	202	101	93	68
C	18353	18713	17586	18695	19054	18184	18670	14317	5044	759	146	80
D	18476	18788	16649	17786	17464	18899	18182	14555	5703	962	158	141
E	19215	18474	16810	17974	16091	7076	1176	300	157	123	154	18877
F	18430	18123	16635	17009	14825	6840	1370	332	188	116	173	18427
G	20215	18980	19383	19436	20880	18089	18310	18079	13331	4400	690	18994
H	17755	18684	18468	18442	17588	18862	18264	18218	14002	4698	858	17651

	1	2	3	4	5	6	7	8	9	10	11	12
A	11788	11376	11394	11098	10830	7151	2571	451	71	36	29	28
B	11172	10458	10537	11117	10877	8188	3319	556	98	50	34	37
C	10667	10943	10310	10959	11154	10658	10939	8390	2951	444	79	45
D	10763	10778	9758	10425	10237	11078	10654	8514	3343	561	82	68
E	11227	10829	9818	10536	9399	4148	682	166	75	57	71	11044

F	10739	10598	9752	9643	8635	4009	796	171	92	65	88	10794
G	11770	11101	11316	11393	12187	10604	10729	10569	7812	2575	390	11128
H	9315	10895	10408	10817	10198	10591	10471	10638	8183	2650	443	10303

	1	2	3	4	5	6	7	8	9	10	11	12
A	70.79	74.19	75.26	76.44	76.55	76.16	74.89	67.1	64.17	53.35	45.01	47.77
B	71.81	73.83	77.45	78.81	76.66	76.3	78.36	71.88	53.3	54.83	39.56	64.22
C	71.38	73.89	76.66	76.55	74.58	77.55	75.46	75.79	74.16	74.48	62.93	67.18
D	72.15	69.18	77.63	76.06	76.34	76.23	75.6	74.07	77.16	72.29	58.9	53.84
E	73.41	76.21	73.25	76.27	73.28	76.4	70.5	64.71	52.77	49.31	49.9	74.18
F	72.24	73.87	76.87	67.74	72.11	77.57	71.32	58.49	53.83	65.83	57.79	75.19
G	71.98	73.96	73.01	76.21	72.93	76.43	75.56	73.72	75.62	74.31	67.29	75.35
H	60.04	72.54	67.05	79.05	70.67	66.63	69.1	73.13	73.51	67.15	58.82	72.97
