# Supplementary material for: Characterization of the Mel1c melatoninergic receptor in platypus (Ornithorhynchus anatinus)
Source: PLoS One. 2018 Mar 12;13(3):e0191904. doi: 10.1371/journal.pone.0191904 (PMC5846726; doi:10.1371/journal.pone.0191904)
Supplement: S1 Data — They are counter listings, formatted to give the individual numbers used to calculate all the saturation curves and affinities reported in the present paper. The plates are all arranged the same ways: Saturation: The 3 first columns are used for increasing low concentrations (nM): A: 0.01; B: 0.02; C: 0.04; D: 0.05; E: 0.08; F: 0.1; G: 0.2; in triplicate. The 3 next columns (4 to 6) were used for the nonspecific binding. The 3 next columns (7 to 9) were used for higher concentrations: A: 0.3; B: 0.4; C: 0.5; D: 0.8; E: 1; F: 1.5 and G: 2. The last 3 columns, same concentrations, nonspecific binding. Nonspecific binding was done in the presence of 10 μM of cold melatonin. The H line was not used. R: 11 concentrations of each product. The concentrations of the products were from 10-14M to 10-4M (from column 1 to 11). Colum 12 is for unspecific binding. Two lines (A&B; C&D, etc.) were used per compounds. For DR in COS7 cell membranes, only 8 compounds were tested in that order from top to bottom: melatonin, 2-iodomelatonin, S 70254, 4P-P-DOT, S 20098/agomelatonin, S 22153, FLN68/ramelteon and Luzindole. For DR in CHO cell membranes in that order from top to bottom: melatonin, 2-iodomelatonin, 6-chlmromeltonin, Luzindole, 4PPDOT, S 20098/agomelatin, FLN68/ramelteon, D600, S20928, S21278, S22153, S70254, S73893, S75436, S27128, DIV880, SD6, SD1881, SD1882 and SD1918. If needed, more information can be obtained from the corresponding author upon request. Table A. Raw data for calculation of COS7 Xenopus Mel1c (n = 1 & 2) saturations. Table B. Raw data for calculation of COS7 Platypus Mel1c (n = 1) saturation. Table C. Raw data for calculation of COS7 Platypus (n = 2) & Xenopus (n = 3) Mel1c saturations. Table D. Raw data for calculation of COS7 Mel1c Platypus (n = 3) and naïve cells saturation. Table E. Raw data for calculation of CO7 Mel1c Chicken (n = 1 & 2) and naïve cells saturations. Table F. Raw data for calculation of CHO Mel1c Xenopus (n = 1) saturation. Table G. [file pone.0191904.s002.zip › Table L.pdf]

Raw data Mel1C Xeno CHO - Mel1C Plat CHO N=2

Ornithorynque Mel1C- Plaque 1

	1	2	3	4	5	6	7	8	9	10	11	12
A	8172	3105	3407	3569	3269	2710	995	243	184	119	129	134
B	3262	3551	3913	3657	3455	2977	1087	317	164	129	179	277
C	3534	3094	3917	3418	2164	644	250	190	378	138	300	202
D	3534	3641	3709	3653	2029	686	279	165	186	103	125	155
E	3626	3576	3923	3979	3739	3657	2193	705	211	125	177	3375
F	3562	3541	3621	3801	3906	3769	2002	590	276	135	198	3579
G	3652	3551	4063	3920	3525	3766	3872	3791	2794	1041	319	3337
H	3471	2606	3278	3538	3507	3662	3758	3691	2826	1061	254	3001

	1	2	3	4	5	6	7	8	9	10	11	12
A	3921	1789	1903	1980	1910	1573	522	132	62	42	51	50
B	1690	1828	1981	1993	1881	1616	575	152	71	50	75	52
C	1838	1669	2041	1830	1252	341	108	85	61	53	49	43
D	1725	2022	1929	1917	1130	346	114	59	65	44	52	40
E	1779	1968	1983	1983	2104	1973	1072	359	103	45	52	1852
F	1703	1924	1933	2026	2249	2038	1086	277	119	65	60	1877
G	1783	1950	2180	2006	2049	1989	2088	2058	1566	545	146	1888
H	1759	1352	1650	1957	2002	2011	2007	1943	1520	571	113	1733

	1	2	3	4	5	6	7	8	9	10	11	12
A	62.54	83.15	78.74	77.88	85.31	84.31	71.08	75.24	40.92	42.44	48.19	45.06
B	69.74	69.12	67.44	75.61	75.48	75.04	72.08	62.71	54.83	47.14	51.78	27.61
C	70.17	74.35	70.39	73.43	83.87	72.02	54.18	56.39	25.79	46.56	25.79	29.27
D	64	77.96	70.15	71.17	78.4	67.03	50.42	43.2	42.66	53.92	50.59	32.76
E	64.48	76.78	67.26	65.93	79.81	74.34	64.15	68.04	63.57	43.32	36.26	76.42
F	62.24	75.21	73.06	72.89	83.06	74.66	74.97	60.76	54.04	62.47	37.53	71.11
G	64.04	76.52	73.68	68.51	84.51	71.88	74.27	75.1	79.2	70.87	58.43	80.49
H	67.5	69.89	66.87	77.47	81.82	76.51	73.17	71.53	73.96	73.93	56.51	83.5

Ornithorynque Mel1C- Plaque 2

	1	2	3	4	5	6	7	8	9	10	11	12
A	7284	3431	3584	3560	3179	3706	3328	3493	2333	689	215	115
B	3548	3556	3725	4099	3713	3877	3278	3695	2078	763	292	172
C	3641	3599	3847	3999	2963	1318	367	286	184	128	126	118
D	3862	3681	3936	3905	3044	1512	470	209	238	104	127	122
E	3565	3568	4126	3782	2135	663	233	171	114	144	115	3776
F	3665	3584	3720	3508	2182	567	268	153	176	122	151	3615
G	3484	3678	3808	3896	3614	4092	4022	3918	2775	923	263	3268
H	3304	3296	3653	3745	3648	3762	3875	3578	2601	852	218	3424

	1	2	3	4	5	6	7	8	9	10	11	12
A	3585	1937	1994	1928	1888	2112	1691	1903	1279	380	88	42

B	1723	1809	1894	2090	2020	2102	1647	2056	1177	390	125	53
C	1873	1950	2020	2165	1714	728	171	131	67	47	46	40
D	1827	2049	2104	2154	1692	821	208	95	74	42	44	42
E	1759	1967	2143	1972	1187	348	99	69	51	58	47	2045
F	1822	1943	1948	1832	1268	306	124	61	76	46	63	1954
G	1769	2048	2109	1737	2104	2147	2140	2152	1567	501	114	1820
H	1667	1822	1952	1947	2021	2130	2027	1949	1441	448	111	1839

	1	2	3	4	5	6	7	8	9	10	11	12
A	64.78	80.22	78.21	74.82	87.86	81.56	67.81	75.56	76.32	76.99	50.21	44.04
B	63.57	67.87	67.83	68.1	75.33	74.97	66.69	78.24	80.62	68.49	53.22	37.6
C	69.05	74.88	71.24	74.76	83.78	77.25	59.93	58.95	43.98	44.03	44.06	40.96
D	61.34	78.3	73.23	77.12	78.13	75.13	56.08	58.49	38.44	49.92	42.13	41.59
E	64.99	77.02	70.04	70.48	78.09	71.24	53.15	49.3	56.33	49.98	50.7	74.82
F	65.68	74.94	70.94	70.61	84.51	74.22	59.34	49.04	54.45	45.11	51.11	74.58
G	67.74	78.36	77.63	56.57	84.78	71.1	72.7	76.59	80.3	74.94	54.38	78.38
H	67.08	77.36	73.2	70.14	77.69	80.59	70.81	75.49	77.69	71.25	67.93	73.81

Ornithorynque Mel1C- Plaque 3

	1	2	3	4	5	6	7	8	9	10	11	12
A	4792	3497	3553	3631	3259	3691	3546	3296	2022	546	206	134
B	3688	3741	4020	4400	3761	3966	4042	3739	2003	644	282	179
C	3847	3676	4110	4422	4017	3883	4172	3851	2952	1221	309	122
D	3754	3631	4022	4050	4001	3881	4076	4003	3091	1260	370	100
E	3681	3867	3991	4318	3711	3916	3661	1933	677	218	163	3632
F	3654	3745	3872	4188	4006	4409	3541	1878	608	195	174	3588
G	3545	3460	3876	4148	3868	3983	3967	3889	2596	473	297	3320
H	3672	3247	3729	3808	3529	3607	3817	3748	2695	431	290	3225

	1	2	3	4	5	6	7	8	9	10	11	12
A	2372	1911	1947	1979	1841	2136	1895	1849	1075	292	87	37
B	1823	1868	1966	2209	2058	2087	1919	2039	1046	341	119	67
C	1888	2033	2186	2381	2299	2031	2246	2113	1652	640	146	52
D	1845	1965	2182	2195	2248	1823	2076	2204	1610	661	172	40
E	1860	2040	2106	2269	2131	2124	1897	1010	322	110	63	1978
F	1843	2095	2099	2194	2297	2394	1873	977	325	97	62	1973
G	1824	1953	2038	2231	2188	2079	2150	2129	1470	231	146	1800
H	1809	1796	1998	2072	2040	2022	2079	2040	1510	223	154	1754

	1	2	3	4	5	6	7	8	9	10	11	12
A	65.31	75.92	76.28	75.6	80.33	83.84	73.2	79.3	72.6	73.5	52.12	34.92
B	65.19	66.13	64.2	66.61	76.04	71.45	61.63	75.68	70.59	72.1	52.73	45.44
C	64.52	77.43	72.68	74.1	82.18	70.81	74.08	76.38	78.96	71.08	61.41	53.54
D	64.65	74.7	75.02	74.92	79.59	60.73	67.99	76.85	70.31	71.18	59.78	48.49
E	67.25	71.76	71.79	71.27	82.66	74.97	69.79	70.68	61.74	67.06	46.99	75.51
F	67.08	78.94	74.92	70.99	82.48	75.11	72.03	70.18	73.33	65.54	42.85	76.72
G	69.06	80.16	71.36	73.98	80.47	70.55	74.89	76.1	80.6	64.11	64.47	74.97

H	64.86	77.46	73.54	75.37	83.63	79.26	75.5	75.43	79.15	69.51	72.64	75.32

Ornithorynque Mel1C- Plaque 4

	1	2	3	4	5	6	7	8	9	10	11	12
A	5238	3877	4113	3988	3631	3996	2945	910	280	108	176	146
B	4276	4477	4155	4625	4279	3968	2994	1134	294	127	183	118
C	4094	4149	4366	4751	4203	4036	2213	815	285	141	177	149
D	4317	4236	4391	4698	4312	3907	2172	765	286	139	136	123
E	4458	4385	4520	4677	4180	2634	947	370	181	166	427	4228
F	4304	4655	4687	4841	3950	2289	922	329	207	98	157	4189
G	4266	4403	4410	4546	4429	4698	4473	4304	2272	396	208	3668
H	4177	3818	4414	4510	3904	4019	4433	4289	2264	430	154	3869

	1	2	3	4	5	6	7	8	9	10	11	12
A	2563	2131	2220	2131	2094	2234	1568	486	133	49	58	66
B	1986	2201	2063	2379	2407	2194	1610	588	132	56	71	48
C	2120	2202	2291	2525	2412	2174	1180	411	130	66	46	48
D	2150	2354	2405	2515	2394	2126	1016	401	134	57	48	43
E	2111	2373	2373	2514	2294	1386	495	175	74	63	174	2275
F	2075	2456	2449	2546	2210	1243	464	167	100	46	57	2233
G	2143	2356	2394	2452	2552	2569	2319	2303	1188	205	80	2023
H	2007	2041	2375	2396	2215	2210	2395	2334	1259	225	74	2075

	1	2	3	4	5	6	7	8	9	10	11	12
A	64.25	76.66	74.4	73.24	83.3	78.88	72.82	73.25	61.61	58.37	39.99	57.86
B	59.85	64.68	65.58	69	79.71	77.38	73.98	69.88	57.02	56.46	47.05	50.85
C	69.7	72.43	71.13	72.59	82.6	74.15	72.91	66.97	58.65	60.3	32.91	39.25
D	65.87	78.09	76.17	73.39	77.94	75.41	60.47	70.93	60.36	50.6	43.03	41.95
E	61.41	74.71	71.18	73.89	76.44	71.46	70.69	61.3	50.52	46.04	50.05	74.01
F	62.92	71.74	70.7	71.38	78.97	75.12	66.75	67.99	63.27	60.3	44.28	72.92
G	66.69	73.36	75.08	74.32	83.18	75.98	69.85	73.39	70.74	69.91	46.68	77.06
H	62.67	73.26	74.03	72.53	80.92	76.71	74.52	75.41	78.19	71.08	62.43	73.66

Ornithorynque Mel1C- Plaque 5

	1	2	3	4	5	6	7	8	9	10	11	12
A	4796	3399	3475	2952	2390	946	384	181	155	86	170	135
B	3793	3813	3761	4021	2819	1248	309	286	149	129	217	187
C	3898	3890	4053	4118	3829	3443	1501	531	220	161	206	127
D	4012	2316	2929	3452	3473	2576	787	466	183	139	131	78
E	3882	3743	4088	4158	3896	4123	3551	2231	860	317	203	3499
F	3956	3932	4172	4141	3925	3986	3702	2485	854	259	145	3494
G	3776	3631	3959	3993	3850	4130	3132	1216	516	162	147	3267
H	3668	3599	3943	3940	3684	3455	3076	1397	340	150	164	3145

	1	2	3	4	5	6	7	8	9	10	11	12
A	2313	1835	1836	1543	1346	513	195	68	58	41	51	58
B	1790	1917	1847	2010	1600	592	150	107	71	49	72	71

C	1975	2072	2129	2167	2107	1903	769	269	93	65	53	51
D	1924	1282	1503	1838	1905	1417	405	224	77	49	49	39
E	1788	1940	2036	2160	2122	2252	1823	1151	436	149	60	1853
F	1823	2063	2186	2168	2251	2160	1951	1279	428	127	66	1783
G	1890	1877	2094	2079	2169	2144	1642	625	271	79	48	1720
H	1829	1919	2047	2046	2125	1879	1637	746	167	63	53	1683

	1	2	3	4	5	6	7	8	9	10	11	12
A	62.97	74.38	71.92	70.76	79.88	74.91	67.86	45.71	45.11	61.92	37.33	54.33
B	61.13	66.77	64.58	66.22	80.96	61.53	63.25	45.28	62.2	45.59	40.47	45.89
C	67.52	72.82	71.27	71.49	76.81	77.38	68.54	67.46	52.37	49.71	32.85	49.04
D	62.49	77.52	68.73	72.77	76.36	76.74	69	62.91	52.55	42.36	45.08	67.9
E	59.18	69.78	65.85	70.05	75.46	75.85	68.8	69.34	67.44	60.87	36.52	72.18
F	59.22	71.14	70.99	70.91	82.49	74.89	71.64	69.08	66.54	64.03	58.93	68.22
G	66.33	69.52	72.03	70.28	79.89	69.95	71.04	68.82	71.03	64.49	39.97	71.53
H	65.97	72.98	69.96	70.02	83.37	75.34	72.78	73.18	64.34	51.48	39.64	73.37

Xénope Laevis Mel1C- Plaque 1

	1	2	3	4	5	6	7	8	9	10	11	12
A	4594	2474	2623	2659	2283	1881	883	239	159	78	173	235
B	2570	2757	3018	2883	2616	1868	841	258	148	165	214	125
C	2652	2496	2866	2879	1721	622	216	226	123	206	126	121
D	2815	2551	2769	2666	1730	665	244	189	155	109	127	100
E	2821	2707	2619	2819	2689	2734	1600	486	227	180	158	2683
F	2704	2702	2766	2909	2857	2662	1489	601	223	118	135	2541
G	2730	2613	2770	2982	2684	2905	2871	2818	2159	847	264	2422
H	2516	2491	2650	2782	2523	2547	2776	2764	2065	735	259	2175

	1	2	3	4	5	6	7	8	9	10	11	12
A	2082	1343	1365	1400	1329	1055	461	112	69	40	65	51
B	1212	1331	1371	1482	1345	995	426	133	66	60	77	56
C	1314	1373	1475	1460	952	329	99	90	51	78	55	57
D	1286	1399	1445	1410	965	351	102	87	61	39	47	51
E	1326	1450	1379	1460	1515	1456	829	249	100	60	51	1379
F	1260	1406	1400	1523	1562	1404	761	298	102	54	58	1316
G	1309	1337	1442	1502	1487	1498	1482	1472	1130	437	105	1304
H	1174	1373	1390	1489	1439	1381	1400	1423	1107	368	131	1127

	1	2	3	4	5	6	7	8	9	10	11	12
A	57.96	75.08	70.21	71.57	84.69	79.28	70.54	60.51	54.84	68.14	45.48	29.5
B	61.12	63.08	58.15	68.97	68.96	72.81	67.51	68.9	56.96	44.11	43.58	56.16
C	65.42	76.72	69.08	67.59	77.47	72.15	59.01	48.71	50.77	45.54	54.68	60.6
D	58.57	76.38	70.53	72.03	78.55	71.82	51.65	59.59	48.26	42.95	44.42	66.92
E	60.83	73.47	71.52	69.71	79.93	72.76	69.73	68.62	55.7	40.94	39.39	68.96
F	60.11	70.25	67.38	70.9	75.99	71.69	68.32	65.32	58.4	58.5	54.58	69.73
G	62.51	68.42	70.26	66.95	77.67	69.29	69.4	70.62	70.84	69.33	48.72	74.1
H	60.21	77.02	71.13	73.43	81.74	74.94	67.02	69.11	73.51	66.37	67.06	69.74

Xénope Laevis Mel1C- Plaque 2

	1	2	3	4	5	6	7	8	9	10	11	12
A	3862	2435	2660	2749	2634	2712	2883	2533	1787	650	245	92
B	2599	2657	2990	3084	2778	2953	3034	2674	1928	684	236	128
C	2586	2653	3031	3135	2523	1350	504	237	223	134	182	89
D	2715	2625	2751	3059	2600	1354	455	168	144	103	146	110
E	2690	2981	2876	2442	1624	539	200	200	154	125	93	2532
F	2481	2658	2938	2798	1578	491	239	143	151	128	111	2221
G	2455	2506	2854	2938	2770	2785	2775	2743	1918	631	196	2295
H	2244	2272	2690	2715	2114	2430	940	2580	1736	615	204	2204

	1	2	3	4	5	6	7	8	9	10	11	12
A	1851	1343	1399	1443	1464	1479	1532	1376	986	325	104	37
B	1230	1329	1458	1551	1469	1467	1494	1442	1032	333	117	48
C	1260	1417	1491	1650	1373	731	231	107	61	62	45	41
D	1272	1427	1396	1625	1396	717	217	76	64	40	48	47
E	1253	1551	1452	1270	875	285	87	84	53	58	44	1337
F	1254	1417	1480	1442	859	247	107	64	59	56	46	1150
G	1213	1291	1450	1508	1539	1468	1498	1437	1029	324	79	1195
H	1100	1183	1364	1350	1177	1235	468	1359	932	314	88	1180

	1	2	3	4	5	6	7	8	9	10	11	12
A	62.41	77.12	71.39	71.18	78.07	75.64	72.56	75.22	77.09	66.28	52.64	49.91
B	61.38	66.29	63.94	66.8	72.01	65.6	64.83	74.3	73.38	63.79	65.14	45.29
C	63.85	73.2	64.74	71.51	75.36	74.79	58.73	57.71	34.05	59.43	31.69	59.8
D	60.53	75.28	67.66	72.55	73.75	72.18	61.91	58.43	56.48	47.6	40.01	54.2
E	60.07	70.24	67.19	70.12	74.22	72.07	54.85	52.01	41.81	59.07	61.15	71.85
F	67.24	72.96	66.95	69.25	75.35	66.74	56.8	56.46	46.98	54.8	51.78	69.75
G	65.11	69.16	67.77	68.79	78.05	71.63	74.38	70.97	73.7	68.72	49.57	70.31
H	64.44	70.34	67.57	65.68	78.33	67.83	65.9	71.56	73.75	68.16	53.91	73.4

Xénope Laevis Mel1C- Plaque 3

	1	2	3	4	5	6	7	8	9	10	11	12
A	3479	2630	2592	2842	2446	2539	3054	2098	1436	690	191	136
B	2589	2610	2880	2873	2531	2415	2647	2730	1810	668	249	180
C	2661	2631	3035	3124	2827	2741	2830	2627	1829	850	284	144
D	2851	2818	2752	2912	2831	2805	2741	2714	1942	690	237	120
E	2816	2866	2789	2901	2931	3016	2681	1709	601	399	139	2305
F	2820	2869	2970	2999	2905	3021	2814	1722	596	231	197	2434
G	2627	2710	2908	3051	2863	2913	2937	2734	1530	226	241	2272
H	2507	2476	2681	2755	2563	2824	2745	2323	1413	223	208	2326

	1	2	3	4	5	6	7	8	9	10	11	12
A	1584	1393	1337	1510	1359	1334	1507	1130	754	355	86	52
B	1249	1266	1335	1359	1352	1236	1349	1451	945	328	110	58
C	1293	1403	1491	1624	1579	1482	1428	1438	975	443	126	45

D	1367	1507	1416	1470	1501	1523	1378	1487	1032	367	106	53
E	1295	1423	1368	1429	1548	1590	1382	886	317	187	60	1186
F	1315	1470	1494	1447	1589	1615	1445	873	300	115	64	1257
G	1269	1402	1559	1590	1545	1532	1569	1447	838	101	108	1221
H	1231	1335	1437	1422	1418	1562	1472	1246	778	102	98	1208

	1	2	3	4	5	6	7	8	9	10	11	12
A	58.31	72.2	69.25	72.56	78.03	71.26	65.01	74.1	71.18	69.16	57.22	46.62
B	63.04	63.46	59.7	61.35	73.17	68.53	68.07	72.57	70.63	64.49	55.71	39.48
C	63.64	72.91	64.58	70.15	78.75	74.62	67.08	76.16	72.93	70.5	56.11	38.57
D	62.44	73.29	69.06	67.13	72.3	75.13	66.79	76.27	72.64	72.56	56.88	55.5
E	59.07	65.58	64.5	64.85	71.9	71.68	69.23	69.8	71.68	60.43	53.97	69.02
F	60.19	68.58	66.77	63.01	76.05	73.22	68.86	67.56	66.98	66.26	39.57	69.43
G	63.09	69.62	73.59	70.37	74.41	71.38	73.22	72.11	76.21	56.91	57.13	73.89
H	64.6	74.32	73.6	69.41	77.51	77.46	73.64	73.63	76.98	58.96	60.92	70.07

Xénope Laevis Mel1C- Plaque 4

	1	2	3	4	5	6	7	8	9	10	11	12
A	3841	2638	2904	2939	2787	2996	2361	1112	425	119	124	106
B	2757	3114	3184	3299	3065	3195	2694	1213	330	132	151	177
C	3064	2880	3246	3490	3311	3190	2324	982	283	144	122	114
D	3265	3211	3199	3168	3336	3166	2356	848	235	150	128	121
E	3041	2734	3082	3283	2644	2114	998	359	173	123	308	2703
F	2949	2968	3137	3343	2581	2012	846	251	174	122	142	2176
G	2735	2901	3225	3166	3012	3680	2919	3071	2440	491	169	2326
H	2768	2620	3060	3325	2837	2998	3492	2797	2043	430	132	2221

	1	2	3	4	5	6	7	8	9	10	11	12
A	1789	1445	1492	1547	1522	1572	1244	602	203	49	44	50
B	1220	1470	1522	1595	1636	1651	1415	594	137	68	56	66
C	1360	1513	1682	1805	1868	1725	1172	497	136	66	48	51
D	1530	1743	1659	1651	1794	1637	1170	434	118	56	48	47
E	1399	1413	1581	1647	1433	1110	494	163	71	53	115	1365
F	1363	1554	1666	1698	1445	1048	415	122	78	49	51	1126
G	1316	1460	1652	1608	1653	1856	1529	1618	1318	242	65	1175
H	1254	1332	1588	1684	1567	1590	1827	1473	1106	219	58	1167

	1	2	3	4	5	6	7	8	9	10	11	12
A	60.08	76.18	68.87	71.54	75.81	71.13	71.67	74.91	62.15	51.32	42.44	60.35
B	55.96	61.18	62.18	63.17	73.08	69.48	71.24	64.34	51.46	68.18	44.7	45.57
C	56.24	71.33	69.8	69.61	80.14	74.62	67.04	67.31	62.4	58.29	47.44	56.88
D	60.54	75.05	69.87	70.37	73.93	69.55	65.61	68.58	66.2	45.2	45.78	46.98
E	59.11	69.53	68.72	66.54	74.94	71.19	65.3	57.78	50.34	53.48	45.26	67.16
F	59.44	70.87	72.51	67.76	79.07	70.39	64.34	63.96	57.67	49.78	43.72	69.64
G	62.75	66.9	68.57	67.75	76.43	67.09	70.92	71.6	74.5	64.83	46.94	67.18
H	57.92	67.86	69.92	67.45	77.27	72.31	70.79	71.52	74.75	68.24	55.5	71.28

Xénope Laevis Mel1C- Plaque 5

	1	2	3	4	5	6	7	8	9	10	11	12
A	4880	124	256	141	768	426	167	122	134	96	283	143
B	2544	1939	1459	2460	2194	1088	351	212	134	136	176	148
C	2720	2705	3008	3150	3110	3042	1646	535	248	219	117	133
D	1617	2923	2321	2540	2130	1755	1268	422	199	144	171	232
E	3107	3058	3076	3238	3186	3271	3173	2446	990	320	148	2484
F	2811	2881	3167	3324	3134	3279	3208	2506	1061	304	208	2384
G	2596	2737	3074	3145	2803	3100	2517	1319	484	203	155	2321
H	2499	2310	2608	2847	2749	2641	2346	1165	404	114	154	2062

	1	2	3	4	5	6	7	8	9	10	11	12
A	2268	43	136	63	391	216	76	61	55	40	52	47
B	1163	998	755	1198	1110	532	153	101	56	61	70	58
C	1320	1448	1503	1660	1728	1607	776	273	111	94	50	51
D	792	1505	1221	1337	1156	953	657	206	85	51	63	88
E	1387	1585	1571	1670	1737	1698	1646	1256	491	163	59	1276
F	1303	1520	1655	1691	1723	1748	1679	1254	555	151	67	1194
G	1250	1478	1585	1616	1539	1629	1324	678	253	88	56	1177
H	1214	1204	1348	1461	1487	1450	1250	602	207	57	48	1077

	1	2	3	4	5	6	7	8	9	10	11	12
A	59.91	42.03	72.49	56.39	68.07	67.47	58.46	65.88	50.12	51.97	27.34	39.94
B	58.59	69.12	69.63	63.84	67.34	64.18	54.52	62.38	51.43	56.57	48.6	47.61
C	63.5	73.43	66.18	71.6	78.06	71.91	61.09	68.14	57.15	54.14	52.97	46.27
D	64.4	69.08	71.43	71.5	75.07	75.1	69.81	64.05	53.18	42.47	44.2	46.07
E	56.7	69.84	68.31	69.3	75.65	69.96	69.89	68.8	65.52	67.88	48.98	68.84
F	59.71	71.75	70.69	67.91	76.68	72.89	70.88	66.29	70.85	65.48	39.44	66.43
G	62.88	74.46	69.28	68.91	76.54	71.3	71.4	68.93	70.73	54.32	43.43	67.57
H	63.62	70.44	69.53	68.78	74.68	76.55	72.85	69.43	68.7	65.17	38.1	70.69

Totaux

	1	2	3	4	5	6	7	8	9	10	11	12
A	0	0	0	427	0	0	0	0	0	60418	64869	62769
B	0	0	0	0	0	0	0	0	0	60463	66306	63083
C	0	0	0	0	0	0	0	0	0	61512	66126	63671
D	0	0	0	0	0	0	0	0	0	61265	66500	62832
E	0	0	0	0	0	0	0	0	0	61238	65743	63554
F	0	0	0	0	0	692	0	0	0	60791	66871	62802
G	0	22	0	0	0	0	0	0	0	61925	65756	63257
H	0	0	0	0	0	0	0	0	0	60938	66490	62060

	1	2	3	4	5	6	7	8	9	10	11	12
A	7	6	8	9	13	10	11	17	9	31333	31355	31083
B	9	9	8	6	15	7	12	11	20	31064	31950	30443
C	11	9	5	5	13	12	13	12	25	31562	31443	30671
D	10	7	6	8	15	10	14	10	25	31368	31586	30567

E	11	7	6	11	10	7	9	6	26	30994	31245	29826
F	10	7	5	9	14	13	9	10	25	31098	32268	29792
G	9	7	6	2	12	6	9	8	25	31402	31277	30094
H	7	7	5	8	10	5	9	9	30	30474	31727	29797

	1	2	3	4	5	6	7	8	9	10	11	12
A	8.82	8.69	7.39	15.48	7.97	8.67	7.75	8.54	8.48	69.87	63.17	65.33
B	7.5	8.54	7.86	5.1	8.23	6.71	7.62	6.49	7.47	68.88	62.9	63.03
C	8.82	13.92	7.59	5.09	8.05	7.4	7.88	13.42	8.39	68.75	61.77	62.87
D	8.37	7.23	6.12	5.13	9.34	7.64	10.81	4.31	9.83	68.53	61.68	63.73
E	8.42	7.76	6.23	8.32	7.06	8.43	7.5	5.4	8.35	67.4	61.73	60.69
F	8.47	6.58	6.42	5.16	7.85	15.25	6.58	6.56	9.31	68.45	63.02	61.57
G	6.48	37.38	10.85	4.09	9.57	9.57	7.67	7.67	8.45	67.58	61.8	61.81
H	5.39	8.79	7.49	5.12	5.75	6.36	6.51	6.36	9.3	66.25	62.07	62.59
