# Supplementary material for: Characterization of the Mel1c melatoninergic receptor in platypus (Ornithorhynchus anatinus)
Source: PLoS One. 2018 Mar 12;13(3):e0191904. doi: 10.1371/journal.pone.0191904 (PMC5846726; doi:10.1371/journal.pone.0191904)
Supplement: S1 Data — They are counter listings, formatted to give the individual numbers used to calculate all the saturation curves and affinities reported in the present paper. The plates are all arranged the same ways: Saturation: The 3 first columns are used for increasing low concentrations (nM): A: 0.01; B: 0.02; C: 0.04; D: 0.05; E: 0.08; F: 0.1; G: 0.2; in triplicate. The 3 next columns (4 to 6) were used for the nonspecific binding. The 3 next columns (7 to 9) were used for higher concentrations: A: 0.3; B: 0.4; C: 0.5; D: 0.8; E: 1; F: 1.5 and G: 2. The last 3 columns, same concentrations, nonspecific binding. Nonspecific binding was done in the presence of 10 μM of cold melatonin. The H line was not used. R: 11 concentrations of each product. The concentrations of the products were from 10-14M to 10-4M (from column 1 to 11). Colum 12 is for unspecific binding. Two lines (A&B; C&D, etc.) were used per compounds. For DR in COS7 cell membranes, only 8 compounds were tested in that order from top to bottom: melatonin, 2-iodomelatonin, S 70254, 4P-P-DOT, S 20098/agomelatonin, S 22153, FLN68/ramelteon and Luzindole. For DR in CHO cell membranes in that order from top to bottom: melatonin, 2-iodomelatonin, 6-chlmromeltonin, Luzindole, 4PPDOT, S 20098/agomelatin, FLN68/ramelteon, D600, S20928, S21278, S22153, S70254, S73893, S75436, S27128, DIV880, SD6, SD1881, SD1882 and SD1918. If needed, more information can be obtained from the corresponding author upon request. Table A. Raw data for calculation of COS7 Xenopus Mel1c (n = 1 & 2) saturations. Table B. Raw data for calculation of COS7 Platypus Mel1c (n = 1) saturation. Table C. Raw data for calculation of COS7 Platypus (n = 2) & Xenopus (n = 3) Mel1c saturations. Table D. Raw data for calculation of COS7 Mel1c Platypus (n = 3) and naïve cells saturation. Table E. Raw data for calculation of CO7 Mel1c Chicken (n = 1 & 2) and naïve cells saturations. Table F. Raw data for calculation of CHO Mel1c Xenopus (n = 1) saturation. Table G. [file pone.0191904.s002.zip › Table M.pdf]

Raw data CHO Mel1c Plat Xeno

Ornithorynque Mel1C- Plaque 1

	1	2	3	4	5	6	7	8	9	10	11	12
A	6444	5411	5542	6016	5746	4668	1780	421	151	181	219	138
B	5502	5700	5462	6353	5526	4305	1851	417	206	125	299	220
C	5421	5488	5930	5911	3996	1471	409	216	113	106	129	177
D	5431	5197	5787	5840	3734	1240	518	223	250	108	154	151
E	5693	5232	5900	6039	5896	5636	3372	979	274	105	201	5998
F	5486	5466	5578	6386	5908	5721	3808	1045	353	190	152	6047
G	5396	5396	5729	6175	5989	6205	6196	5996	5274	2284	580	5833
H	5504	5355	5120	5737	5721	5739	6146	5867	4872	2381	521	5501

CPM	1	2	3	4	5	6	7	8	9	10	11	12
A	3283	2928	2903	3262	3181	2598	924	209	64	50	58	75
B	2703	2851	2420	3307	3032	2331	931	219	80	46	50	80
C	2684	2832	3180	3100	2232	780	200	92	53	46	52	69
D	2705	2785	3022	3070	2109	617	223	100	84	43	52	70
E	2826	2802	2985	3040	3288	2893	1675	499	130	51	59	3083
F	2673	2817	2908	3199	3289	3065	2000	539	164	72	64	3275
G	2640	2748	3040	3301	3362	3241	3337	3179	2881	1249	272	3117
H	2771	2663	2770	2958	3189	3133	3239	3151	2613	1228	239	3071

	1	2	3	4	5	6	7	8	9	10	11	12
A	68.03	74.7	70.94	74.96	77.58	78.3	69.91	65.78	52.82	34.41	33.22	74.44
B	64.59	66.26	56.08	70.25	76.43	74.79	66.85	70.97	47.17	44.08	26.25	44.19
C	65.31	69.34	73.63	71.08	78.73	72.32	64.27	52.99	60.58	53.55	49.67	47.33
D	65.87	73.57	70.63	71.35	80.3	65.67	53.77	57.5	40.86	48.22	40.78	59.86
E	65.57	73.47	67.34	66.88	78.53	68.81	65.61	68.19	61.48	62.86	36.71	68.92
F	63.86	69.18	70.43	66.4	78.33	73.51	71.24	69.36	59.95	45.89	52.32	74.81
G	64.22	68.03	72.38	73.26	79.43	70.64	74.13	72.29	75.89	75.99	60.67	73.21
H	66.89	65.73	74.69	69.26	78.49	75.79	71.63	73.79	73.66	69.27	59.04	78.69

Ornithorynque Mel1C- Plaque 2

	1	2	3	4	5	6	7	8	9	10	11	12
A	5829	5793	5962	5885	5780	6258	6315	6022	2926	696	282	162
B	6173	6086	6246	6719	6228	6391	6421	6343	3461	786	330	136
C	5956	5916	6170	6448	5549	2998	1027	398	234	167	136	167
D	5992	6236	6330	6434	5543	3080	902	273	190	124	156	164
E	6026	5898	6203	6383	4407	1496	388	185	187	153	171	6289
F	6005	5967	6419	6230	4811	1435	473	178	223	138	179	6240
G	5862	5733	6077	6425	5921	6383	6369	6162	5272	2249	458	6029
H	5378	5383	5853	6003	5982	5952	6067	5916	4750	1810	448	5516

CPM	1	2	3	4	5	6	7	8	9	10	11	12
A	2881	3134	3167	3225	3314	3537	3434	3315	1587	355	129	61
B	2923	3067	3086	3392	3534	3376	3386	3445	1912	422	137	68
C	3056	3211	3267	3400	3100	1651	501	186	111	59	48	62
D	2993	3322	3249	3457	3117	1662	467	129	79	61	61	69
E	2882	3206	3242	3342	2419	745	177	86	86	61	56	3401
F	3017	3189	3486	3326	2693	748	217	83	91	56	61	3299
G	2959	3093	3337	3415	3376	3384	3386	3273	2699	1207	210	3235
H	2687	2928	3078	3215	3348	3186	3228	3224	2524	982	212	3006

	1	2	3	4	5	6	7	8	9	10	11	12
A	65.16	74.67	72.52	76.25	82.45	80.39	75.3	76.86	75.02	68.12	59	45.79
B	61.41	66.98	65.12	67.16	80.95	71.88	71.7	75.17	77.31	73.66	51.62	66.02
C	68.76	75.08	72.14	71.69	78.76	76.93	63.9	60.42	61.66	42.87	42.68	44.53
D	66.13	72.85	68.8	73.85	79.67	74.4	69.69	61.14	51.66	64.59	47.78	51.72
E	62.24	75.23	70.72	70.92	76.48	65.9	58.29	60.46	58.96	48.58	39.86	74.63
F	66.68	73.21	75.14	73.1	79.02	70.48	59.05	59.9	50.28	50.17	41.15	71.98
G	67.14	74.33	76.54	72.59	81.64	72.3	72.61	72.52	68.53	73.71	58.87	73.71
H	66.15	75.32	71.38	73.47	79.02	73.41	72.73	75.57	72.57	75	61.36	75.58

Ornithorynque Mel1C- Plaque 3

	1	2	3	4	5	6	7	8	9	10	11	12
A	6045	6214	6461	6849	6155	6335	6781	6555	4010	1612	284	149
B	6496	6324	6519	7119	6623	7107	6988	6672	4338	1419	317	166

C	6122	6142	7073	6668	6787	6464	6821	6790	5323	2152	535	131
D	6595	6469	6853	7152	6756	6643	6917	6838	5580	2086	469	165
E	6504	6569	7409	6902	6618	6730	6576	3729	1034	354	189	6824
F	6430	6675	7147	6913	6857	6843	6569	3859	1166	332	230	6906
G	6187	6070	6822	6858	6496	6799	6750	6605	4597	394	924	6352
H	6154	5900	6325	6955	6395	6726	7181	6477	4622	447	911	5772

CPM	1	2	3	4	5	6	7	8	9	10	11	12
A	2983	3398	3543	3678	3525	3517	3642	3583	2229	852	129	64
B	3130	3310	3257	3576	3616	3563	3691	3593	2366	772	153	75
C	3155	3400	3684	3442	3723	3567	3547	3719	2871	1160	256	59
D	3329	3553	3462	3795	3803	3628	3593	3723	2959	1116	228	72
E	3257	3609	3745	3559	3637	3557	3383	1966	552	179	77	3693
F	3088	3625	3735	3634	3802	3695	3449	2110	627	170	89	3704
G	3133	3283	3647	3601	3617	3552	3599	3589	2518	204	470	3451
H	2997	3246	3416	3715	3568	3584	3784	3509	2576	225	456	3228

	1	2	3	4	5	6	7	8	9	10	11	12
A	65	75.99	76.34	73.8	82.29	77.94	73.8	75.95	78.11	71.98	58.26	53.61
B	62.88	70.87	66.17	66.67	75.81	66.48	71.89	74.14	75.67	75.35	62.82	57.53
C	69.19	77.55	70.34	69.37	76.39	77.15	70.15	76.2	74.32	74.21	62.19	58.22
D	67.14	76.55	67.21	72.39	79.8	75.82	70.04	75.46	72.33	73.32	63.75	54.98
E	66.38	76.61	67.28	69.27	76.61	71.96	69.02	71.68	73.09	67.29	49.96	74.72
F	62.63	75.16	70.71	71.34	77.76	74.45	71.22	76.01	73.85	68.23	46.6	73.66
G	67.45	74.66	73.27	71.22	78.34	70.66	72.94	75.2	76.21	69.96	67.79	75.2
H	63.83	76.76	74.47	73.17	78.58	72.89	71.61	74.84	78.43	66.86	66.27	78.93

Ornithorynque Mel1C- Plaque 4

	1	2	3	4	5	6	7	8	9	10	11	12
A	6142	5622	6273	6639	6463	6389	5331	2301	478	157	157	150
B	6035	6195	6455	6998	6248	6749	5698	2421	569	179	181	135
C	5980	6168	6757	7047	6701	7142	4210	1559	505	183	164	185
D	6310	6046	6336	6744	6665	6250	4133	1372	411	171	158	152
E	6277	6037	6490	6597	6366	4567	1738	595	189	140	1121	7051
F	6147	6050	6678	6730	6517	4393	1853	470	231	134	182	6366
G	5976	5928	6235	6852	6307	6705	7035	6436	2540	419	240	6462
H	5628	5763	6624	6884	6398	6827	6677	6692	2220	298	274	6352

CPM	1	2	3	4	5	6	7	8	9	10	11	12
A	2893	3088	3368	3540	3693	3506	2661	1238	247	73	58	58
B	2883	3141	3168	3475	3334	3383	2966	1280	299	77	67	61
C	2953	3384	3611	3770	3655	3775	2209	821	228	88	55	68
D	3060	3265	3218	3559	3722	3415	2103	725	215	82	59	63
E	3106	3218	3327	3427	3404	2291	910	284	86	60	509	3791
F	2899	3102	3566	3486	3272	2323	950	236	99	59	64	3532
G	2862	3142	3340	3603	3513	3461	3686	3357	1338	198	99	3581
H	2722	3113	3307	3572	3523	3461	3560	3599	1243	142	104	3320

	1	2	3	4	5	6	7	8	9	10	11	12
A	60.97	76.56	73.77	72.96	81.93	76.44	66.06	74.03	69.29	60.16	44.41	46.94
B	62.15	67.56	64.51	65.61	73.05	66.47	70.26	71.99	71.32	53.7	44.96	57.09
C	65.06	76.4	73.22	73.32	75.67	71.95	71.15	71.53	57.56	63.22	40.56	44.44
D	63.46	74.45	67.72	71.79	78.71	75.91	67.89	71.92	70.84	61.88	45.14	50.84
E	65.27	72.91	68.65	70.05	73.29	66.54	70.92	62.24	58.64	53.95	58.14	73.94
F	61.08	68.69	73.15	69.74	66.61	72.01	68.64	66.62	53.65	55.6	42.87	77.87
G	62.38	72.27	73.5	71.37	78.39	69.37	70.98	70.51	71.62	61.29	50.79	77.71
H	63.23	74.48	66.1	69.93	76.86	67.55	72.97	73.97	79.09	62.17	46.05	70.69

Ornithorynque Mel1C- Plaque 5

	1	2	3	4	5	6	7	8	9	10	11	12
A	4810	5209	5624	5939	5068	3652	876	229	176	95	148	127
B	5580	5781	5846	6222	5195	3275	751	235	225	145	198	88
C	5177	5227	5432	6424	5510	5293	3271	889	550	263	271	154
D	4623	3781	3323	3784	4159	2775	2622	725	570	312	141	124
E	5492	5251	5393	5702	5495	5502	5596	3974	1369	378	162	5539
F	5533	5532	5612	5608	5443	5607	5878	3853	1373	354	218	5723
G	4920	5062	5347	5582	5359	5326	5069	2330	678	190	212	5164
H	5037	4736	5174	5383	5036	5126	4962	2390	552	131	212	5424

CPM	1	2	3	4	5	6	7	8	9	10	11	12
A	2312	2839	2933	3148	2895	1933	409	107	62	40	45	42
B	2600	2795	2745	3133	2732	1625	366	112	90	54	63	41
C	2563	2796	2777	3165	2865	2917	1643	440	250	119	41	51
D	2184	2033	1712	1903	2259	1459	1246	354	261	138	41	53
E	2700	2745	2767	2932	2965	2699	2811	2062	722	173	72	2897
F	2580	2828	2980	2798	3010	2997	2992	2004	721	178	71	2955
G	2459	2680	2817	2849	2984	2656	2636	1209	345	81	63	2780
H	2415	2522	2749	2741	2759	2692	2584	1248	279	63	88	2833

	1	2	3	4	5	6	7	8	9	10	11	12
A	62.69	75.59	70.48	72.26	81.93	72.12	60.24	60.3	42.46	51.69	37.31	40.62
B	60.1	63.19	60.73	66.91	71.39	65.49	63.96	62.1	48.9	45.16	38.86	59.45
C	65.31	73.31	68.37	64.88	70.16	77	66.67	65.29	58.29	58.07	25.02	40.51
D	61.21	73.94	69.15	66.79	75.19	71.36	61.75	64	58.88	56.22	35.87	53.88
E	64.67	70.74	68.75	68.98	74.37	64.49	66.69	69.94	71.76	58.71	56.17	70.79
F	60.16	68.36	72.48	66.04	77.45	73.24	67.95	70.2	71.25	67.01	39.65	69.4
G	66.18	72.14	71.58	68.24	78.32	65.98	70.15	69.89	68.01	53.13	37.05	74.09
H	62.46	72.82	72.56	67.98	76.22	71.24	70.32	70.59	67.14	63.23	51.2	70.66

Xénope Laevis Mel1C- Plaque 1

	1	2	3	4	5	6	7	8	9	10	11	12
A	1949	1297	1394	1377	1395	960	421	139	87	136	191	102
B	1333	1398	1410	1558	1455	1102	448	190	183	239	202	129
C	1396	1408	1689	1897	1116	421	201	163	200	125	237	142
D	1489	1549	1589	1542	1090	453	211	150	134	161	146	90
E	1383	1392	1500	1554	1475	1479	906	327	215	137	133	1256
F	1420	1376	1456	1621	1431	1516	872	361	216	114	191	1392
G	1302	1274	1473	1609	1512	1466	1683	1423	1135	459	250	1193
H	1220	1160	1294	1292	1289	1331	1456	1319	1180	444	237	1229

CPM	1	2	3	4	5	6	7	8	9	10	11	12
A	920	614	695	702	765	513	202	59	39	42	50	42
B	614	710	659	657	730	529	183	90	55	48	84	52
C	652	744	711	807	562	203	77	76	69	50	43	37
D	618	723	769	690	570	194	73	43	48	36	36	35
E	574	610	695	678	691	666	394	145	92	51	41	644
F	630	657	703	825	753	744	417	166	79	42	58	646
G	585	611	709	781	765	704	810	691	590	230	73	593
H	587	578	539	596	567	661	743	632	519	196	66	608

	1	2	3	4	5	6	7	8	9	10	11	12
A	61.15	61.41	66	68.12	76.33	73.3	62.46	52.53	57.6	38.03	32.96	51.57
B	59.24	67.7	60.38	52.36	66.52	62.55	50.11	61.71	36.92	28.4	50.99	49.46
C	60.26	71.94	52.29	53.03	66.87	63.06	46.31	60.4	42.15	49.4	27.23	32.98
D	51.32	60.3	63.34	56.93	70.82	53.43	42.14	36.08	43.13	30.3	31.78	47.78
E	51.31	55.27	59.65	54.87	60.6	57.46	54.65	56.26	53.12	45	37.98	68.63
F	56.2	62.14	63.11	67.92	71.43	64.49	62.17	59.23	44.47	44.61	37.55	59.83
G	57.25	62.53	62.77	63.51	67.28	62.58	62.75	63.65	70.12	66.54	36.18	65.77
H	62.82	65.88	51.51	59.36	55.52	65.67	68.25	62.36	55.45	56.01	34.64	65.29

Xénope Laevis Mel1C- Plaque 2

	1	2	3	4	5	6	7	8	9	10	11	12
A	3075	1447	1535	1675	1673	1700	1724	1428	766	234	178	155
B	1703	1539	1637	1686	1600	1931	1692	1591	792	274	265	164
C	1505	1518	1686	1890	1701	939	386	262	210	190	104	152
D	1563	1565	1832	1739	1750	1060	407	163	141	144	145	97
E	1524	1412	1786	1544	1102	432	252	138	192	137	174	1449
F	1488	1631	1665	1655	1155	501	232	199	199	149	159	1462
G	1505	1544	1685	1759	1617	1777	1804	1693	1469	534	199	1484
H	1466	1260	1471	1555	1521	1507	1702	1667	1121	433	219	1572

CPM	1	2	3	4	5	6	7	8	9	10	11	12
A	1506	776	785	863	794	887	883	699	347	108	72	55
B	749	752	779	874	873	966	865	833	413	129	107	60
C	736	771	853	906	887	477	187	114	71	81	41	65
D	748	812	872	879	948	535	163	68	61	52	55	48
E	740	764	849	764	512	209	108	45	83	58	48	739

F	687	835	858	833	626	238	109	78	77	58	59	749
G	718	738	845	862	869	837	920	842	746	247	78	695
H	705	666	755	729	774	780	837	859	566	215	79	732

	1	2	3	4	5	6	7	8	9	10	11	12
A	64.31	73.67	68.41	69.21	61.62	70.47	68.6	64.31	57.95	59.8	49.86	42.88
B	55.53	64.14	61.81	69.81	75.7	66.25	68.31	70.88	70.55	61.31	49.55	44.59
C	64.25	67.74	67.41	62.44	70.5	67.7	63.34	54.91	41.18	52.76	48.16	53.55
D	62.25	69.86	61.86	67.34	74.8	67.21	48.94	51.45	53.85	43.99	45.7	65.68
E	63.53	74.61	61.78	65.25	59.9	63.31	53.73	39.7	54.61	52.95	34.35	68.06
F	59.36	68.58	69.24	66.87	74.78	61.65	60.7	47.92	47.28	47.2	44.85	68.61
G	62.03	62.26	66.57	64.42	73.81	60.98	68.1	65.79	67.74	59.47	47.4	60.57
H	62.72	72.01	68.77	60.66	67.89	69.7	64.68	69.16	67.24	65.62	43.78	60.05

Xénope Laevis Mel1C- Plaque 3

	1	2	3	4	5	6	7	8	9	10	11	12
A	1978	1216	1495	1440	1447	1535	1480	1487	1104	317	162	100
B	1495	1552	1535	1611	1472	1650	1627	1609	1273	420	148	259
C	1318	1429	1713	1779	1606	1638	1626	1538	1066	450	171	90
D	1525	1469	1720	1587	1506	1676	1425	1703	964	508	188	67
E	1507	1374	1497	1546	1448	1635	1513	1042	333	135	129	1398
F	1444	1513	1513	1527	1545	1471	1586	945	373	89	111	1388
G	1583	1358	1583	1635	1606	1784	1803	1426	629	418	618	1442
H	1211	1282	1426	1582	1479	1530	1426	1469	622	428	635	1402

CPM	1	2	3	4	5	6	7	8	9	10	11	12
A	919	569	687	646	707	784	619	700	472	145	45	34
B	667	749	618	754	652	789	694	725	563	184	57	32
C	557	614	742	789	789	752	710	638	467	175	45	33
D	707	630	701	665	698	693	590	844	476	219	80	30
E	621	590	610	626	642	729	666	474	158	52	34	623
F	639	710	628	647	703	621	706	469	168	45	27	660
G	670	548	652	679	815	748	828	618	334	198	249	586
H	508	633	639	744	674	690	667	624	300	197	256	587

	1	2	3	4	5	6	7	8	9	10	11	12
A	59.84	60.45	58.99	57.1	64.08	68.3	51.78	60.91	53.34	58.62	34.92	41.5
B	56.61	63.09	49.33	60.54	56.08	62.22	53.18	57.52	55.99	55.35	47.07	22.92
C	52.53	53.77	54.34	56.18	64.58	58.9	54.99	51.22	55.26	47.35	33.25	44.91
D	59.71	53.55	50.1	52.01	59.73	51.04	51.18	65.42	65.03	53.93	53.11	56.7
E	50.76	53.68	50.05	49.71	56.16	56.59	55.6	58.3	61.62	46.31	33.45	56.49
F	55.97	60.72	51.31	52.67	58.3	52.43	56.41	65.5	57.55	68.01	31.33	61.81
G	52.68	49.5	50.74	51.28	67.72	52.01	58.96	54.36	72.4	61.57	49.35	49.96
H	52.03	65.11	56.99	60.85	58.36	57.58	60.45	52.92	63.1	59.14	49.56	51.89

Xénope Laevis Mel1C- Plaque 4

	1	2	3	4	5	6	7	8	9	10	11	12
A	3741	1407	1620	1623	1551	1528	1247	664	183	88	112	117
B	1589	1529	1587	1652	1506	1535	1286	672	181	86	85	120
C	1512	1591	1546	1623	1519	1569	1229	472	201	109	93	87
D	1550	1594	1532	1707	1702	1557	1107	422	180	110	94	148
E	1414	1566	1662	1597	1487	1361	592	237	145	135	124	1528
F	1477	1453	1549	1679	1631	1408	541	210	132	135	1804	1405
G	1480	1574	1628	1774	1592	1775	1633	1559	840	136	149	1437
H	1528	1524	1618	1674	1636	1813	1809	1574	863	128	153	1272

CPM	1	2	3	4	5	6	7	8	9	10	11	12
A	1596	738	838	860	839	788	629	348	90	30	36	31
B	675	730	755	785	803	777	650	326	73	39	35	44
C	718	792	765	807	812	797	595	223	82	46	35	35
D	751	838	783	854	893	814	530	200	83	42	40	33
E	684	813	744	784	762	667	268	96	55	37	38	745
F	667	758	793	806	839	682	258	101	45	40	748	709
G	632	827	809	835	833	853	808	783	435	64	59	726
H	703	807	834	823	848	904	888	804	425	50	52	643

	1	2	3	4	5	6	7	8	9	10	11	12
A	53.24	71.09	69.66	72.23	74.65	69.28	67.16	71	64.73	41.52	39.18	33.69

B	52.9	62.07	61.83	61.66	72.87	67.48	67.32	63.45	49.04	56.83	49.93	43.86
C	61.66	65.82	65.26	65.72	73.22	67.77	63.38	61.17	50.59	51.76	45.65	49.58
D	63.39	71.31	68.42	66.28	71.11	70.73	62.38	61.63	59.43	46.41	52.9	29.86
E	63.21	69.96	56.88	64.59	68.55	64.32	57.83	49.61	45.6	33.88	37.36	63.96
F	57.6	70.59	68.49	62.54	68.99	63.36	61.91	62.86	41.18	36.4	51.23	67.12
G	53.25	71.23	65.73	60.9	70.8	62.67	65.26	66.73	69.84	61.45	48.04	67.15
H	59.1	72.2	69.24	64.7	69.82	66	64.51	68.37	64.92	46.98	41.53	67.2

Xénope Laevis Mel1C- Plaque 5

	1	2	3	4	5	6	7	8	9	10	11	12
A	2729	1193	1209	1183	930	604	201	90	147	74	154	679
B	1188	1181	1227	1249	951	556	225	112	203	119	131	357
C	1199	1663	1295	1194	1849	1201	728	279	137	83	124	167
D	1400	1113	1389	1240	1532	1326	753	252	152	62	172	92
E	1367	1385	1417	1473	1409	1368	1514	1078	557	195	150	1216
F	1140	2475	2230	1304	1308	1250	1379	1001	489	189	143	1114
G	1138	1357	2538	1282	1184	1265	1109	566	236	95	108	1138
H	1129	1145	1741	1231	1241	1202	1105	555	240	85	175	1036

CPM	1	2	3	4	5	6	7	8	9	10	11	12
A	1187	605	623	573	501	286	90	42	31	24	38	33
B	519	562	544	613	485	257	88	35	39	31	38	30
C	590	839	656	601	940	603	349	117	53	32	34	36
D	620	586	665	628	814	667	377	119	50	26	32	36
E	626	681	654	733	731	695	726	518	259	83	41	604
F	523	1209	1071	628	689	624	668	494	228	93	48	535
G	549	684	1225	625	645	625	520	276	105	37	42	536
H	492	562	787	593	643	608	544	264	102	33	33	515

	1	2	3	4	5	6	7	8	9	10	11	12
A	54.65	67.61	69.22	63.26	74.32	61.37	56.7	59.87	29.06	39.07	31.82	17.47
B	54.94	61.81	56.16	64.56	68.11	59.53	47.49	38.57	27.81	32.62	35.71	20.16
C	64.72	67.16	67.45	66.84	67.82	66.58	62.48	51.89	46.52	46.13	34.01	29.49
D	56.09	71.45	62.39	67.47	72.56	66.9	66.48	61	39.82	52.76	27.68	48.49
E	58.72	64.7	59.33	65.8	69.94	67.82	62.51	62.71	59.9	53.38	33.84	65.59
F	58.83	64.11	62.59	62.84	71.61	66.17	63.32	65.03	60.23	64.54	40.65	62.53
G	62.93	66.98	63.06	63.9	75.55	65.13	60.64	63.79	56.05	47.13	47.38	60.9
H	54.84	64.58	57.79	62.84	69.74	67.31	64.83	61.87	53.36	46.97	27.92	65.66

Totaux

	1	2	3	4	5	6	7	8	9	10	11	12
A	0	0	0	0	0	0	0	0	0	80164	82744	78991
B	0	0	0	0	0	0	0	0	0	80036	86764	76706
C	0	0	0	0	0	0	0	0	0	80460	83244	79240
D	1230	0	0	0	0	0	0	0	0	81581	84325	78094
E	0	0	0	0	0	0	0	0	0	79268	83039	78399
F	0	0	0	0	0	0	57	0	0	79784	83877	77385
G	0	0	0	0	0	0	0	0	0	80184	82593	77422
H	0	0	0	0	0	0	0	0	0	79000	82451	75550

	1	2	3	4	5	6	7	8	9	10	11	12
A	8	12	7	13	16	15	10	27	18	37437	37210	35898
B	6	9	5	5	18	12	11	9	39	37963	38475	34414
C	10	9	9	6	11	12	10	11	36	37617	36322	34055
D	6	5	6	6	9	12	14	10	38	37112	35643	33488
E	7	4	4	5	11	9	9	10	42	37598	37800	34699
F	8	5	5	3	9	7	14	10	40	37808	37685	34583
G	7	7	7	4	6	6	7	7	27	36463	36610	34183
H	9	6	3	5	12	6	12	8	25	36397	36509	32896

	1	2	3	4	5	6	7	8	9	10	11	12
A	9.56	9.13	8.24	7.19	6.91	5.34	7.6	7.56	8.4	60.29	57.3	58.16
B	5.39	11.23	7.33	5.06	10.37	7.48	8.77	5.4	10.52	61.56	56.15	57.1
C	6.52	7.52	8.03	5.17	7.6	8.4	8.5	7.56	10.35	60.38	54.89	53.75
D	14.28	9.14	7.87	6.15	9.05	11.82	9.54	6.57	10.9	58.24	52.56	53.59
E	10.44	5.3	6.13	11.12	6.91	8.48	6.76	7.27	10.67	61.56	58.29	56
F	7.38	11.23	-1	6.12	5.89	6.11	31.13	6.47	10.6	61.49	57.22	56.78
G	7.49	7.49	7.39	6.35	10.12	7.3	6.76	12.3	9.34	58.21	56.12	55.81
H	5.52	12.61	6.23	7.24	7.96	11.77	8.69	7.46	10.19	59.22	56.04	54.73
